# TCAF2 in Pericytes Promotes Colorectal Cancer Liver Metastasis via Inhibiting Cold‐Sensing TRPM8 Channel

**DOI:** 10.1002/advs.202302717

**Published:** 2023-08-27

**Authors:** Xiaobo Li, Qi Qi, Yong Li, Qun Miao, Wenqian Yin, Jinghua Pan, Zhan Zhao, Xiaoying Chen, Fan Yang, Xiaofeng Zhou, Maohua Huang, Chenran Wang, Lijuan Deng, Dandan Huang, Ming Qi, Shuran Fan, Yiran Zhang, Shenghui Qiu, Weiqing Deng, Tongzheng Liu, Minfeng Chen, Wencai Ye, Dongmei Zhang

**Affiliations:** ^1^ State Key Laboratory of Bioactive Molecules and Druggability Assessment Jinan University Guangzhou 510632 China; ^2^ College of Pharmacy Jinan University Guangzhou 510632 China; ^3^ MOE Key Laboratory of Tumor Molecular Biology Clinical Translational Center for Targeted Drug Department of Pharmacology School of Medicine Jinan University Guangzhou 510632 China; ^4^ School of Pharmacy North Sichuan Medical College Nanchong 637100 China; ^5^ Department of General Surgery The First Affiliated Hospital of Jinan University Guangzhou 510632 China; ^6^ Department of Biophysics Kidney Disease Center of First Affiliated Hospital Zhejiang University School of Medicine Hangzhou 310058 China; ^7^ Formula‐Pattern Research Center School of Traditional Chinese Medicine Jinan University Guangzhou 510632 China; ^8^ The Sixth Affiliated Hospital of Sun Yet‐Sen University Guangzhou 510655 China

**Keywords:** isolation, metastasis, microdissection, TCAF2, tumor pericytes

## Abstract

Hematogenous metastasis is the main approach for colorectal cancer liver metastasis (CRCLM). However, as the gatekeepers in the tumor vessels, the role of TPCs in hematogenous metastasis remains largely unknown, which may be attributed to the lack of specific biomarkers for TPC isolation. Here, microdissection combined with a pericyte medium‐based approach is developed to obtain TPCs from CRC patients. Proteomic analysis reveals that TRP channel‐associated factor 2 (TCAF2), a partner protein of the transient receptor potential cation channel subfamily M member 8 (TRPM8), is overexpressed in TPCs from patients with CRCLM. TCAF2 in TPCs is correlated with liver metastasis, short overall survival, and disease‐free survival in CRC patients. Gain‐ and loss‐of‐function experiments validate that TCAF2 in TPCs promotes tumor cell motility, epithelial‐mesenchymal transition (EMT), and CRCLM, which is attenuated in pericyte‐conditional *Tcaf2*‐knockout mice. Mechanistically, TCAF2 inhibits the expression and activity of TRPM8, leading to Wnt5a secretion in TPCs, which facilitates EMT via the activation of the STAT3 signaling pathway in tumor cells. Menthol, a TRPM8 agonist, significantly suppresses Wnt5a secretion in TPCs and CRCLM. This study reveals the previously unidentified pro‐metastatic effects of TPCs from the perspective of cold‐sensory receptors, providing a promising diagnostic biomarker and therapeutic target for CRCLM.

## Graphical abstract

TCAF2 is highly expressed in tumor pericytes (TPCs) derived from patients with colorectal cancer liver metastasis (CRCLM), which induces the secretion of Wnt5a through inhibiting TRPM8 channel and activates the STAT3 phosphorylation in tumor cells, thus facilitating CRCLM. Activating TRPM8 with menthol suppresses CRCLM. This study provides a diagnostic marker and effective treatment for CRCLM.

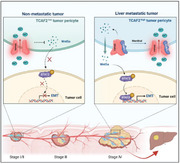

## 1 Introduction

Liver metastasis is the leading cause of disease‐related death among patients with colorectal cancer (CRC),^[^ ^1^, ^2^ ^]^ and identification of potential biomarkers is important for the diagnosis and treatment of colorectal cancer liver metastasis (CRCLM). Hematogenous spread is the primary metastatic event of CRCLM, during which tumor cell intravasation from the primary tumor is characterized by tumor cells penetrating the endothelial wall of blood vessels to enter the vasculature, initiating the metastatic cascade.^[^ ^3^ ^]^ Therefore, identifying the mechanisms underlying tumor cell intravasation may facilitate the development of effective strategies for the diagnosis and treatment of tumor metastasis. Current investigations on tumor intravasation mainly focus on the interaction between tumor cells and endothelial cells.^[^ ^4^ ^]^ Tumor pericytes (TPCs) are important vascular components embedded outside the vessel lumen and serve as gatekeepers of tumor vessels.^[^ ^5^ ^]^ However, the regulatory effect of TPCs on tumor cell intravasation remains largely unclear, and no TPC‐related molecular markers have been reported as biomarkers for CRCLM.

Multi‐omics is an effective approach for discovering novel biomarkers,^[^ ^6^ ^]^ and obtaining pure TPCs is a basic premise for multi‐omic analysis. However, owing to their low proportion, high heterogeneity,^[^ ^7^ ^]^ and lack of unique molecular markers,^[^ ^5^ ^]^ isolation of TPCs with high purity from tumor tissue remains challenging. Traditional methods for TPC isolation utilize enzyme digestion combined with fluorescence‐activated cell sorting (FACS) or magnetic bead cell sorting (MACS) by antibodies against surface antigens, including CD146, CD248, platelet‐derived growth factor receptor beta (PDGFRβ), and NG2.^[^ ^8^, ^9^, ^10^, ^11^ ^]^ However, the above antigens, as well as other recognized TPC markers, such as Desmin, fibroblast activation protein alpha (FAPα), and alpha‐smooth muscle actin (αSMA), are not specific but are also expressed in cancer‐associated fibroblasts (CAFs).^[^ ^12^ ^]^ Given that CAFs in the tumor microenvironment are much more than TPCs, TPCs isolated from tumor tissues by FACS or MACS are frequently contaminated with CAFs, which cannot meet the requirement for further multi‐omic analysis and functional studies. In addition, TPCs are heterogeneous and exhibit distinct features at different stages of cancer.^[^ ^13^ ^]^ Therefore, FACS‐ or MACS‐obtained TPCs are unable to reflect their heterogeneity, which hinders TPC‐related research. Therefore, it is imperative to develop a novel isolation and culture method to obtain pure TPCs.

Currently, most studies focus on the expression and function of cell surface receptors, such as CD248,^[^ ^14^ ^]^ PDGFRβ,^[^ ^8^ ^]^ integrins,^[^ ^15^ ^]^ secreted cytokines,^[^ ^16^ ^]^ and metabolic enzymes^[^ ^17^ ^]^ in TPCs. Nevertheless, the expression and regulatory effects of ion channel proteins and their partner proteins in TPCs have not yet been elucidated. TRP channel‐associated factor 2 (TCAF2) is a partner protein of the transient receptor potential cation channel subfamily M member 8 (TRPM8), which is a primary cold sensor that regulates the response to cold adaptation in human tissues.^[^ ^18^, ^19^, ^20^ ^]^ To date, few studies have evaluated the role of TCAF2 in cancer. *TCAF2* is a hypoxia‐related gene with unknown function in lung and breast cancers.^[^ ^21^, ^22^ ^]^ TCAF2 binds to TRPM8 to promote its trafficking to the cell surface and to inhibit its ion channel activity, thereby increasing the migration of prostate cancer cells in vitro.^[^ ^20^ ^]^ However, the role of TCAF2 in TPCs and its regulatory effects on cancer development remain largely unknown.

Here, we developed a new method called microdissection combined with pericyte medium‐based approach (MPMA) to successfully obtain TPCs from primary tumor tissues surgically removed from CRC patients. Proteomic analysis revealed that TCAF2 was overexpressed in TPCs derived from patients with CRCLM, and its expression in TPCs was negatively correlated with prolonged overall survival (OS) and disease‐free survival (DFS). Pericyte‐specific deletion of *Tcaf2* suppressed CRC cell epithelial‐mesenchymal transition (EMT) and inhibited tumor metastasis. TCAF2 in TPCs inhibited the expression and ion channel activity of TRPM8, promoting tumor cell EMT and metastasis via activation of the Wnt5a/STAT3 signaling pathway. Menthol, an agonist of the TRPM8 channel, significantly suppressed CRCLM by inhibiting Wnt5a secretion. Our data indicate that TCAF2 and TRPM8 are promising predictive biomarkers and therapeutic targets for CRCLM.

## 2 Results

### 2.1 Isolation, Culture, and Characterization of TPCs

**Figure 1:**
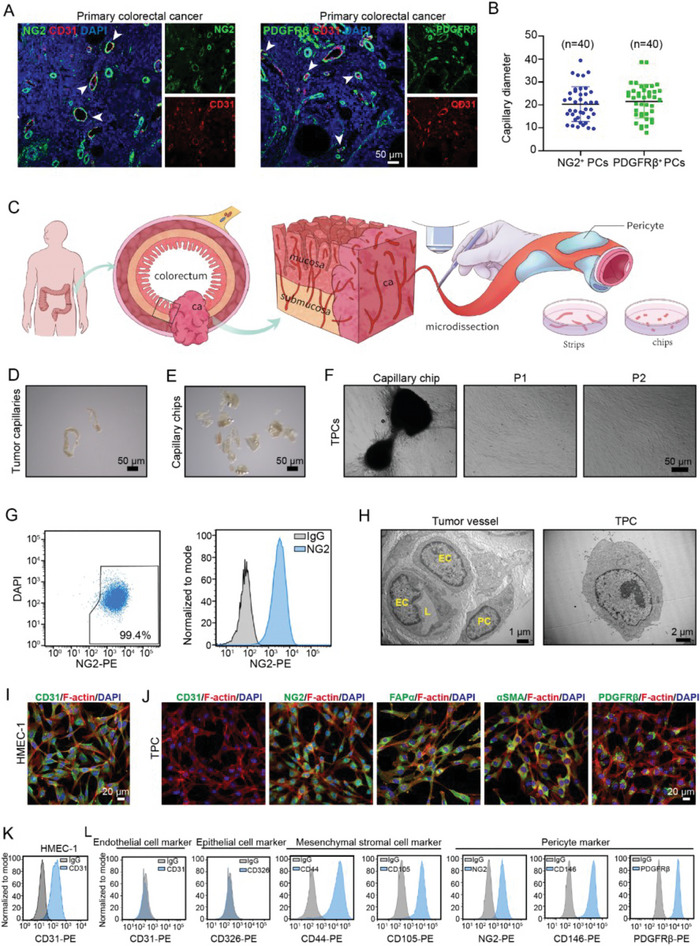
Isolation and validation of TPCs. A) Immunofluorescence staining for PC markers including NG2 or PDGFRβ (green) in tumor blood vessels (CD31, red) with various diameters (n = 40). The white arrowhead indicates the PCs‐containing capillaries. Scale bar, 50 µm. B) Plot diagram of the diameters in PCs‐containing capillaries (n = 40). Data are presented as mean ± SEM. C) Diagram depicting the isolation of tumor blood vessels from CRC patients. D) Representative images of blood vessels dissected from tumor tissues under the stereomicroscope (n = 6). Scale bar, 50 µm. E) Representative images for the capillary chips (n = 6). Scale bar, 50 µm. F) Representative images for TPC migration and expansion from the cultured capillary chips (n = 6). Scale bar, 50 µm. G) Flow cytometry analysis of NG2 expression in TPCs at passage 2 (n = 6). H) Representative TEM images of the tumor vessels and TPCs (n = 6). Scale bar, 1 µm (left); 2 µm (right). I) Immunofluorescence analysis of CD31 expression in HMEC‐1 cells. Phalloidin‐rhodamine was used to identify F‐actin. Scale bar, 20 µm. J) Immunofluorescence analysis of indicated markers in TPCs (n = 6). Phalloidin‐rhodamine was used to identify F‐actin. Scale bar, 20 µm. K) Flow cytometry analysis of CD31 in HMEC‐1 cells (n = 6). L) Flow cytometry analysis for the expression of endothelial cells, epithelial cells, mesenchymal stromal cells, and pericyte markers in cultured TPCs (n = 6).

Tumor vessels are mainly neoplastic blood capillaries that contain endothelial cells (ECs) and pericytes (PCs).^[^ ^23^ ^]^ To provide a criterion for the isolation of TPCs, we first identified TPC‐harboring capillaries in tumor tissues using the recognized PC antigens NG2 and PGDFRβ.^[^ ^24^ ^]^ Our results showed that tumor capillaries with diameters between 10 and 40 µm were mainly covered with NG2^+^ and PDGFRβ^+^ TPCs (**Figure** **1**A,B). With the above criteria, the capillaries containing TPCs and ECs were resected from freshly surgical tumor specimens using microsurgical scissors under an anatomic microscope under sterile condition (Figure 1C). The attached adipose tissues were removed to obtain capillaries with thin and translucent walls (Figure 1D), which were then cut into “mini‐chips” for cell isolation (Figure 1E). The “mini‐chips” were then seeded in six‐well plates and cultured with PM, which is suitable for the proliferation of PCs, but not ECs (Figure S1, Supporting Information). Following seven‐day culture, multiple cell clusters crawled out from the “mini‐chips” and were collected as the first passage (P1) cells, which were characterized as TPCs by single cell‐RNA sequencing (scRNA‐seq) (Figure S2, Supporting Information).^[^ ^25^ ^]^ By passage 2 (P2), ≈ 2 × 10^6^ cells were obtained (Figure 1F), which were determined by flow cytometry. More than 99% of the P2 cells were positive for NG2 (Figure 1G). Hence, the above TPC isolation method was termed microdissection combined with pericyte medium‐based approach (MPMA).

Next, we determined the purity of the cells isolated by MPMA. Short tandem repeat (STR) analysis showed that these cells were of one type without contamination (Table S1, Supporting Information). Transmission electron microscopy was performed to further assess the morphological features of the MPMA‐obtained cells. Consistent with accepted features,^[^ ^26^ ^]^ TPCs were embedded outside the lumen lined with ECs characterized by Weibel–Palade bodies in tumor vessels,^[^ ^27^ ^]^ which showed no fibers at the cell membrane margin and a high nuclear/cytoplasmic ratio with rare organelles in the cytoplasm. The MPMA‐isolated cells exhibited characteristics similar to TPCs in tumor sections (Figure 1H). Moreover, the MPMA‐obtained TPCs were spindle‐like or finger‐like in shape and positive for NG2, FAPα, αSMA, and PDGFRβ, but negative for the EC marker CD31 (Figure 1I,J). Flow cytometry analysis confirmed that the MPMA‐obtained TPCs did not express CD31 or epithelial marker CD326, but highly expressed pericyte makers, including NG2, PDGFRβ, and CD146, as well as mesenchymal stromal cell antigens CD44 and CD105^[^ ^28^, ^29^ ^]^ (Figure 1K,L). Moreover, the MPMA‐obtained TPCs were double positive for PDGFRβ/NG2 or PDGFRβ/CD146 (Figure S3, Supporting Information). Taken together, these data indicate that the cells isolated by MPMA are TPCs.

To determine the advantages of MPMA in the isolation of TPCs, the cells obtained by MPMA were compared with those obtained using the MACS method. Flow cytometric analysis confirmed that > 98% of the cells sorted by MACS were positive for NG2 (Figure S4A,B, Supporting Information). Similar to the MPMA‐obtained TPCs, NG2‐sorting cells did not express CD31 and CD326; however, the expression of mesenchymal stromal cell markers CD44 and CD105, as well as the pericyte markers CD146 and PDGFRβ in the MPMA‐obtained TPCs was higher than those in NG2‐sorting cells (Figure S4C, Supporting Information). The STR report indicated that NG2^+^ cells sorted by MACS were contaminated with other types of human cells (Table S2, Supporting Information). In addition, compared with the MPMA‐obtained TPCs, a large number of NG2^+^ cells sorted by MACS failed to attach to the lumen formed by HMEC‐1 cells (Figure S4D, Supporting Information), further indicating that these sorted cells were mixed with cells other than TPCs. Given that NG2 is also highly expressed in certain CAF populations and has been used to identify CAFs,^[^ ^30^ ^]^ the NG2‐sorting cells may contain CAFs. Therefore, the expression of CAF markers^[^ ^31^ ^]^ including PDGFRα, S100A4, COL1A2, COL3A1, and decorin was examined in these two cell types. Our results showed that the expression of CAF markers was significantly increased in NG2‐sorting cells compared to MPMA‐obtained TPCs (Figure S4E, Supporting Information), indicating that NG2‐sorting cells were contaminated with CAFs. Taken together, these data indicate that MPMA may be an alternative method to obtain pure TPCs.

### 2.2 TCAF2 in TPCs is Associated with Liver Metastasis of CRC

**Figure 2:**
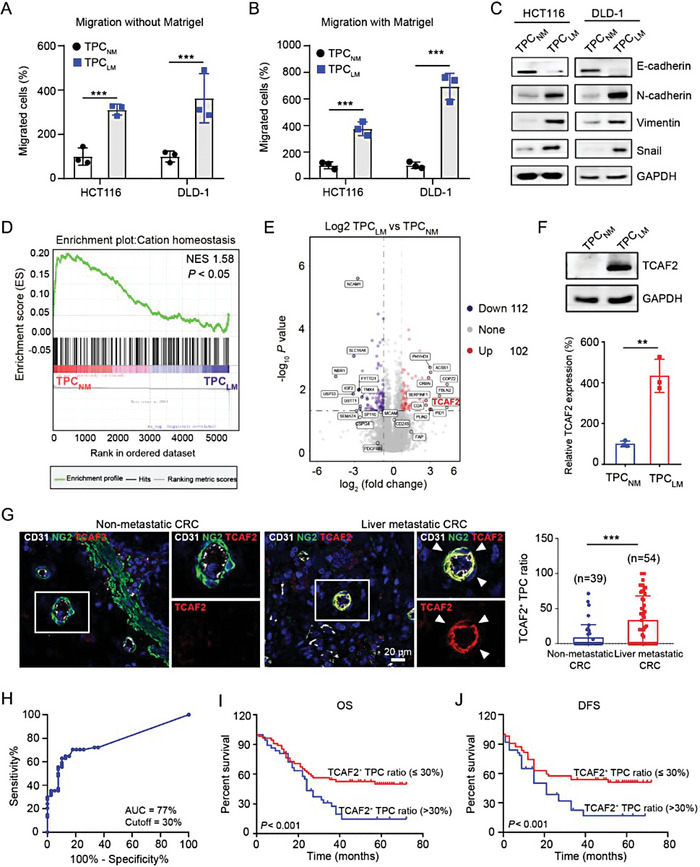
TCAF2 is highly expressed in TPCs and associated with CRCLM. A,B) Transwell assay for the migration without (A) or with (B) Matrigel of CRC cells primed with the conditioned medium from TPC_NM_ and TPC_LM_, respectively (n = 3). Scale bar, 100 µm. C) Western blot analysis of E‐cadherin, N‐cadherin, Vimentin, and Snail in HCT116 and DLD‐1 cells primed with conditioned medium from TPC_NM_ and TPC_LM_, respectively. D) GSEA plots displaying the gene set of cation homeostasis were negatively enriched in TPC_LM_. E) The volcanic plot of proteins with upregulated (red) and downregulated (purple) expression in TPC_NM_ and TPC_LM_ (log_2_ (fold change) > 1.5, *p*‐value < 0.05; n = 3). F) Western blot analysis of TCAF2 in TPC_NM_ and TPC_LM_ (n = 3). G) Representative immunofluorescence images of TCAF2 (red), NG2 (green), and CD31 (grey) in primary tumor sections derived from CRC patients. The white arrowhead indicates the TCAF2 expression in TPCs. Scale bar, 20 µm. Quantification of TCAF2^+^ TPC ratio is shown. H) ROC curve analysis for the TCAF2^+^ TPC ratio in CRCLM (n = 93). I) Kaplan–Meier OS curves for patients with a high or low ratio of TCAF2^+^ TPCs (based on the 30% cutoff, n = 93). J) DFS curves for patients with a high or low ratio of TCAF2^+^ TPCs (based on the 30% cutoff, n = 93). Data are presented as mean ± SEM. **p < 0.01, ***p < 0.001 by two‐tailed unpaired *t*‐test in (A,B,F); by Mann–Whitney *U* test in G; *p* < 0.001 versus indicated groups by Log‐rank (Mantel–Cox) test in (I,J).

To determine the role of TPCs in liver metastasis of CRC, TPCs isolated from CRC patients with or without liver metastasis were named TPC_LM_ and TPC_NM,_ respectively. We found that TPC_NM_ and TPC_LM_ expressed the same levels of the PC markers (Figure S5A, Supporting Information) and exhibited similar proliferation (Figure S5B, Supporting Information), and migration (Figure S5C, Supporting Information) abilities. However, compared to TPC_NM_, the conditioned medium of TPC_LM_ significantly promoted the migration (**Figure** **2**A,B; Figure S5D–G, Supporting Information), and EMT (Figure 2C; Figure S5H, Supporting Information) of various CRC cells including HCT116, DLD‐1, SW480, and SW620 cells, indicating that TPC_LM_ exerted profound pro‐metastatic effects on CRCLM. To further investigate the mechanism underlying the pro‐metastatic effect of TPC_LM_, TPC_NM_, and TPC_LM_ were subjected to tandem mass tag (TMT)‐based quantitative proteomic analysis. Approximately 214 differentially expressed proteins were identified. Among them, 102 proteins were upregulated in TPC_LM_. Gene set enrichment analysis (GSEA) revealed that genes associated with cation homeostasis were markedly enriched in TPC_NM_ compared to TPC_LM_ (Figure 2D). However, the role of cation homeostasis in TPCs remains unclear. Strikingly, TCAF2, as the binding protein of the TRPM8 ion channel, was the second most highly expressed protein in TPC_LM_ compared to that in TPC_NM_ (Figure 2E), which was confirmed by quantitative real‐time PCR (Figure S6, Supporting Information), and Western blot (Figure 2F). Furthermore, we found that the TCAF2^+^ TPC ratio was increased in tumor tissues derived from CRC patients with liver metastasis (Figure 2G). However, TCAF2 was undetectable in the TPCs of hepatic metastatic nodules derived from CRC patients (Figure S7, Supporting Information). ROC curve analysis was performed to obtain the optimal cut‐off value of the TCAF2^+^ TPC ratio, which was determined to be 30% for predicting CRCLM with relatively high sensitivity and specificity (Figure 2H). Patients with a high TCAF2^+^ TPC ratio (> 30%) showed a worse OS (Figure 2I) and DFS (Figure 2J). Similar results were observed in primary tumor sections from breast cancer patients with pulmonary metastases (Figure S8, Supporting Information). These data demonstrate that TCAF2 in TPCs can serve as a predictive biomarker of tumor metastasis.

We further investigated the underlying mechanisms by which TCAF2 was upregulated in TPC_LM_. First, we examined whether the cancer driver mutations or any other cancer markers correlated with TCAF2 expression in TPCs using the clinical data of CRC patients with or without liver metastasis. Data showed that the TCAF2^+^ TPC ratio was associated with liver metastasis and TNM stage, but not with *KRAS* or *BRAF* mutations (Table S3, Supporting Information). In addition, TPC_NM_ were primed with the conditional medium from CRC cells with *KRAS* mutation (DLD‐1, HCT116, LoVo, SW480, SW620) and those with *BRAF* mutation (HT‐29, WiDr). The results showed that only the conditional medium from LoVo cells, a cell line derived from metastatic nodules resected from a male patient with grade IV Dukes C CRC, specifically induced TCAF2 upregulation in TPC_NM_ (Figure S9, Supporting Information), indicating the TCAF2 expression in TPCs was not correlated with *KRAS‐* or *BRAF‐*mutations in CRC cells, which might be associated with the high‐metastatic property of tumor cells. Furthermore, with scRNA‐seq data from TPC_LM_ and TPC_NM_, GO analysis revealed that the upregulated genes in TPC_LM_ were associated with “response to hypoxia” and “response to decreased oxygen levels” (Figure S10A, Supporting Information). Hypoxia is a common feature of the tumor microenvironment caused by abnormal vascular structure and tumor cell metabolism,^[^ ^32^ ^]^ which is more pronounced in high‐metastatic tumors than that in low‐metastatic tumors, and the tumor blood vessels and surrounding area are also subject to hypoxia condition in high‐metastatic tumors.^[^ ^33^, ^34^, ^35^, ^36^ ^]^ Hypoxia has been reported to upregulate TCAF2 level in cancer cells.^[^ ^21^ ^]^ Therefore, we proposed that the upregulation of TCAF2 in TPC_LM_ might be associated with tumor hypoxia. To test this hypothesis, hypoxia condition in TPCs was first examined in primary tumor tissues derived from patients with CRC. Our results showed that the hypoxia condition was more pronounced in primary tumor of CRCLM compared with that in CRC without metastasis. Moreover, CAIX, a marker of hypoxia, was highly expressed in TPCs in the primary tumor of CRCLM (Figure S10B,C, Supporting Information), indicating that TPCs were under hypoxia condition during CRCLM. Subsequently, the expression of TCAF2 was examined in TPCs treated with or without hypoxia. Compared to the normoxic condition (5% O_2_), the hypoxic condition (1% O_2_) significantly increased the expression of HIF‐1α and TCAF2 in TPC_NM_ (Figure S10D, Supporting Information). In addition, overexpression of HIF‐1α markedly increased the expression of TCAF2 in TPC_NM_ under normoxic condition, whereas HIF‐1α knockdown decreased the expression of TCAF2 in TPC_NM_ under hypoxic condition (Figure S10E, Supporting Information). Furthermore, the ChIP‐qPCR assay indicated that hypoxic condition enhanced the binding of HIF‐1α to the *TCAF2* promoter (Figure S10F, Supporting Information). Taken together, our results demonstrate that the expression of TCAF2 in TPCs is regulated by high‐metastatic potential of tumor cells and the hypoxic condition.

### 2.3 TCAF2 in TPCs facilitates Cell Motility and Liver Metastasis of CRC

To determine the effect of TCAF2^+^ TPCs on tumor metastasis, TPC_NM_ with an intrinsically low level of TCAF2 were transfected with a TCAF2 overexpressing plasmid (TPC_NM_^TCAF2^) (Figure S11A,B, Supporting Information), and TPC_LM_ with naturally higher TCAF2 expression were transfected with short hairpin RNA (shRNA) to construct TCAF2‐knockdown TPCs (TPC_LM_^shTCAF2^) (Figure S11C,D, Supporting Information). Although TCAF2 had negligible effects on the proliferation and motility of TPCs (Figure S12A,B, Supporting Information), and neither the overexpression nor depletion of TCAF2 in TPCs altered the proliferation, migration, and tube formation of endothelial cells in the co‐culture models (Figure S13, Supporting Information), the conditioned medium of TPC_NM_^TCAF2^ promoted the migration of HCT116 and DLD‐1 cells compared to those induced by TPC_NM_^Vector^ medium (Figure S14A,B, Supporting Information), whereas the conditioned medium of TPC_LM_^shTCAF2^ suppressed the migration of HCT116 and DLD‐1 cells compared to those induced by TPC_LM_^shNC^ medium (Figure S14C,D, Supporting Information). Consistently, compared with TPC_NM_^Vector^, the conditioned medium of TPC_NM_^TCAF2^ enhanced the expression of mesenchymal markers, including Vimentin and Snail, accompanied by decreased epithelial marker E‐cadherin in HCT116 and DLD‐1 cells, whereas the conditioned medium of TPC_LM_^shTCAF2^ suppressed the expression of Vimentin and Snail, accompanied by increased E‐cadherin in HCT116 and DLD‐1 cells compared with TPC_LM_^shNC^ medium (Figure S14E,F, Supporting Information).

**Figure 3:**
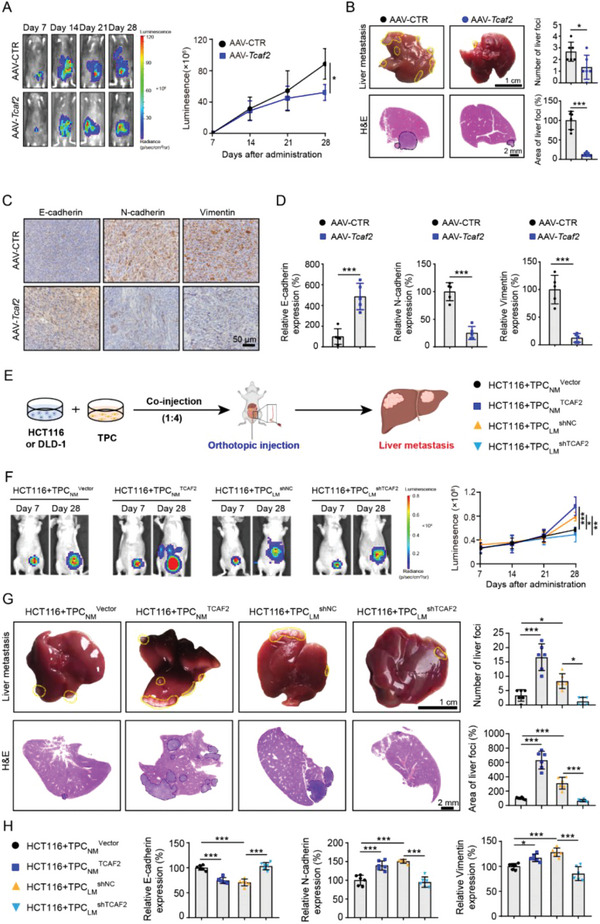
TCAF2^+^ TPCs promote colorectal cancer cell motility and liver metastasis by induction of EMT. A) Representative images and quantification of bioluminescence signals in mice orthotopically injected with MC38‐luc cells on various days (n = 6). B) Representative images and H&E analysis of liver metastases derived from MC38‐luc allografts (n = 6). Yellow and black dotted lines indicate the liver metastatic foci. Scale bar, 1 cm (up); 2 mm (down). C,D) Immunohistochemical staining and quantification of EMT markers in primary tumor sections derived from MC38‐luc allografts (n = 5). Scale bar, 50 µm. E) A schematic diagram describing the animal experiment. HCT116 or DLD‐1 cells mixed with the indicated TPCs at a ratio of 1:4 were co‐injected into the cecum wall of mice to construct the CRCLM xenografts. F) Representative images and quantification of bioluminescence signals in mice co‐injected with TPCs and HCT116‐luc cells (n = 6). G) Representative images and quantification of liver metastases derived from CRCLM xenografts (n = 6). Yellow and black dotted lines indicate the liver metastatic foci. Scale bar, 1 cm (up); 2 mm (down). H) Immunohistochemical analysis of EMT markers in primary tumor sections derived from CRCLM xenografts (n = 6). Data are presented as mean ± SEM. *p < 0.05, ***p < 0.001 by two‐tailed unpaired *t*‐test in (A,B,D); by one‐way ANOVA followed by Tukey's post hoc test in (F,G,H).

To evaluate whether endogenous TCAF2 in TPCs is essential for CRC metastasis in vivo, pericyte‐*Tcaf2* conditional knockout mice were generated using *Cspg4*‐CreERT2 mice intravenously injected with adeno‐associated virus (AAV)‐*Tcaf2* (Figure S15A, Supporting Information). Immunofluorescence assay showed that AAV‐*Tcaf2* injection depleted the expression of TCAF2 in TPCs compared with AAV‐CTR (Figure S15B, Supporting Information). To determine the effect of TCAF2 in TPCs on CRC metastasis, MC38‐luc cells were injected into the cecum walls of mice administered AAV‐CTR or AAV‐TCAF2. The metastatic behavior of the MC38‐luc xenografts was monitored using a bioluminescence imaging system (**Figure** **3**A). Our results showed that deletion of TCAF2 in TPCs suppressed CRC metastasis, as indicated by the decreased number of circulating tumor cells (CTCs) (Figure S15C, Supporting Information), and liver metastases (Figure 3B). Moreover, the expression of N‐cadherin and Vimentin was decreased, whereas the expression of E‐cadherin was increased in the AAV‐*Tcaf2* group (Figure 3C,D). However, pericyte‐specific deletion of *Tcaf2* had negligible effects on tumor microvessel density (MVD), vascular size (Figure S15D,E, Supporting Information), pericyte coverage (Figure S15F, Supporting Information), basement membrane integrity (Figure S15G, Supporting Information), vessel permeability (Figure S15H, Supporting Information), and tumor hypoxia (Figure S15I, Supporting Information).

To further determine the role of TCAF2^+^ TPCs in tumor metastasis in vivo, HCT116 or DLD‐1 cells pre‐mixed with TPC_NM_^Vector^, TPC_NM_^TCAF2^, TPC_LM_^shNC^ or TPC_LM_^shTCAF2^ were orthotopically transplanted into the cecum wall of nude mice to construct liver metastatic xenografts (Figure 3E). The co‐injected TPCs were located in the periphery of tumor vessels and played a dominant role at the endpoint (Figure S16, Supporting Information), and TCAF2 in TPCs had negligible effects on MVD, vascular size (Figure S17A, Supporting Information), pericyte coverage (Figure S17B, Supporting Information), the integrity of vascular basement membrane (Figure S17C, Supporting Information), and tumor hypoxia (Figure S18, Supporting Information) in orthotopic xenografts. Nevertheless, compared with TPC_NM_^Vector^, co‐injection with TPC_NM_^TCAF2^ enhanced tumor metastasis, as indicated by the increased number of CTCs (Figure S19A–C, Supporting Information) and liver nodules (Figure 3F,G; Figure S19D,E, Supporting Information), whereas co‐injection with TPC_LM_^shTCAF2^ showed the opposite effects compared to TPC_LM_^shNC^ (Figure 3F,G; Figure S19, Supporting Information). Moreover, the expression of N‐cadherin and Vimentin was increased, and the level of E‐cadherin was decreased in the TPC_NM_^TCAF2^ co‐injection group, whereas knockdown of TCAF2 in TPC_LM_ suppressed the expression of N‐cadherin and Vimentin, accompanied by an increased level of E‐cadherin (Figure 3H; Figure S20, Supporting Information).

To investigate the effects of TCAF2 in TPCs on metastatic behavior in patient‐derived xenograft models, primary tumor cells were isolated from patients with non‐metastatic CRC (CRC_NM_) and liver metastatic CRC (CRC_LM_). CRC_NM_ cells were then co‐injected with TCAF2‐overexpressing TPC_NM_, whereas CRC_LM_ cells were co‐injected with TCAF2‐knockdown TPC_LM_ into the cecum of nude mice. Our results showed that the co‐injection of TPC_NM_^TCAF2^ facilitated liver metastasis (Figure S21A, Supporting Information), and EMT (Figure S21B, Supporting Information) of CRC_NM_ cells compared with those in the TPC_NM_^Vector^ co‐injection group, whereas the co‐injection of TPC_LM_^shTCAF2^ suppressed liver metastasis (Figure S21C, Supporting Information) and EMT (Figure S21D, Supporting Information) of CRC_LM_ cells compared with those in the TPC_LM_^shNC^ co‐injection group. These data demonstrate that TPCs promote tumor cell motility and EMT, facilitating liver metastasis of CRC in a TCAF2‐dependent manner.

### 2.4 TCAF2 in TPCs Facilitates CRCLM via the Paracrine Wnt5a Signaling Pathway

**Figure 4:**
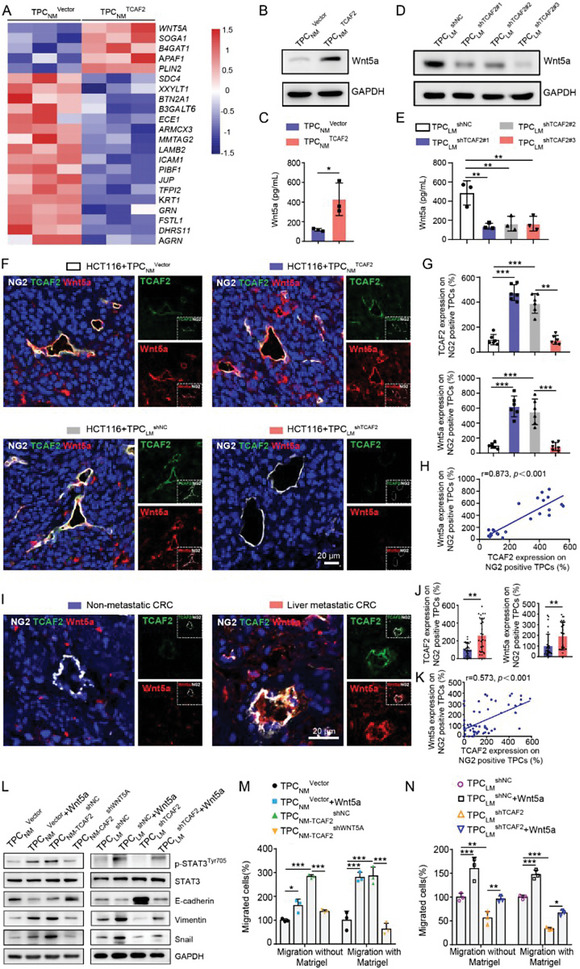
TCAF2^+^ TPCs promote tumor cell motility and EMT via the Wnt5a/STAT3 axis. A) Heatmap of the top 22 cytokines in TPC_NM_^Vector^ and TPC_NM_^TCAF2^ identified by proteomics (log_2_ (fold change) > 1.5, *p*‐value < 0.05; n = 3). B) Western blot analysis of Wnt5a in TCAF2‐overexpressing TPCs (n = 3). C) ELISA assay for Wnt5a secretion in the culture medium of TCAF2‐overexpressing TPCs (n = 3). D) Western blot analysis of Wnt5a in TCAF2‐knockdown TPCs (n = 3). E) ELISA assay for Wnt5a secretion in the culture medium of TCAF2‐knockdown TPCs (n = 3). F) Immunofluorescence analysis for the colocalization of TCAF2 (green) and Wnt5a (red) in TPC (NG2, gray) in tumor sections derived from CRCLM xenografts (n = 6). Scale bar, 20 µm. G) Quantification of TCAF2 and Wnt5a expression in NG2^+^ TPCs. H) Pearson's correlation analysis for Wnt5a and TCAF2 expression in NG2^+^ TPCs. *p* < 0.001. I) Immunofluorescence analysis showing the colocalization of Wnt5a (red) and TCAF2 (green) in TPCs (NG2, gray) in primary tumor sections derived from CRC patients with or without liver metastasis (n = 30). Scale bar, 20 µm. J) Quantification of TCAF2 and Wnt5a expression in NG2^+^ TPCs. K) Pearson's correlation analysis for Wnt5a and TCAF2 expression in NG2^+^ TPCs. *p* < 0.001. L) Western blot analysis of p‐STAT3^Tyr705^, STAT3, and EMT markers in HCT116 cells primed with the conditioned medium from TPC_NM_^Vector^, TPC_NM_^Vector^+Wnt5a (500 ng mL^−1^), TPC_NM‐TCAF2_^shNC^, TPC_NM‐TCAF2_^shWNT5A^, TPC_LM_^shNC^, TPC_LM_^shNC^+Wnt5a (500 ng mL^−1^), TPC_LM_^shTCAF2^, or TPC_LM_^shTCAF2^+Wnt5a (500 ng mL^−1^). M,N) Transwell assay for the migration (with or without Matrigel) of HCT116 cells primed with the conditional medium from the indicated TPCs (n = 3). Data are presented as mean ± SEM. *p < 0.05, **p < 0.01, and ***p < 0.001 by two‐tailed unpaired *t*‐test in (C); by Mann–Whitney *U* test in (J); by one‐way ANOVA followed by Tukey's post hoc test in (E,G,M,N).

Next, the tandem mass tag (TMT)‐based quantitative proteomic analysis was employed to determine the mechanism by which TCAF2^+^ TPCs in the regulation of tumor metastasis. Our results showed that five cytokines were highly upregulated in TPC_NM_^TCAF2^, of which Wnt5a was the most remarkable one (**Figure** **4**A). Consistently, the expression and conditional secretion of Wnt5a were increased in TPC_NM_^TCAF2^ compared with those in TPC_NM_^Vector^ (Figure 4B,C). In contrast, TCAF2 knockdown in TPCs suppressed the secretion of Wnt5a (Figure 4D,E). Similar results were obtained in tumor xenografts. The expression of TCAF2 and Wnt5a in NG2^+^ TPCs were increased in the group co‐injected with HCT116 cells and TPC_NM_^TCAF2^ compared to those with TPC_NM_^Vector^. In contrast, co‐injection of HCT116 cells with TPC_LM_^shTCAF2^ resulted in lower expression of TCAF2 and Wnt5a in NG2^+^ TPCs than in the TPC_LM_^shNC^ group (Figure 4F,G). Pearson's correlation analysis indicated that the expression of Wnt5a was positively correlated with TCAF2 in NG2^+^ TPCs of CRCLM xenografts (Figure 4H). Additionally, Wnt5a expression was significantly increased in TPC_LM_ compared to that in TPC_NM_ (Figure S22A, Supporting Information). Immunofluorescence staining also showed that the expression of TCAF2 and Wnt5a in NG2^+^ TPCs was significantly increased in primary tumor tissues derived from CRC patients with liver metastasis compared with those without liver metastasis (Figure 4I,J). Furthermore, Pearson's correlation analysis indicated that Wnt5a was positively correlated with the expression of TCAF2 in NG2^+^ TPCs from CRC clinical specimens (Figure 4K). Collectively, these results indicate that TCAF2 induces Wnt5a production in TPCs.

Given that Wnt5a can promote tumor cell EMT and metastasis by activating the STAT3 signaling pathway,^[^ ^37^ ^]^ we next investigated whether TCAF2 in TPCs enhanced CRC cell motility and EMT through the Wnt5a/STAT3 signaling pathway. Consistent with Wnt5a treatment alone (Figure S22B–D, Supporting Information), the conditional medium of TPC_NM‐TCAF2_^shNC^ (TPC_NM_^TCAF2^ transfected with shNC) induced EMT and activation of STAT3 signal transduction in HCT116 and DLD‐1 cells. In contrast, shWNT5A reversed the effect of TPC_NM‐TCAF2_, as indicated by attenuated EMT and decreased expression of p‐STAT3^Tyr705^ in HCT116 and DLD‐1 cells (Figure 4L; Figure S23A, Supporting Information). As a result, the conditional medium of TPC_NM‐TCAF2_^shWNT5A^ (TPC_NM_^TCAF2^ transfected with shWNT5A) significantly suppressed cell migration compared to TPC_NM‐TCAF2_^shNC^ (Figure 4M; Figure S23B,C, Supporting Information). In contrast, the conditional medium of TPC_LM_^shTCAF2^ suppressed EMT and the Wnt5a/STAT3 signaling axis, leading to attenuated cell migration compared to those regulated by TPC_LM_^shNC^ medium, while the addition of recombinant Wnt5a reversed the effects of TPC_LM_^shTCAF2^ in CRC cells (Figure 4L,N; Figure S23D,E, Supporting Information). Taken together, these data indicate that TCAF2 in TPCs induces Wnt5a secretion and activates STAT3, thus promoting tumor cell EMT and facilitating CRCLM.

### 2.5 TCAF2 Inhibits the Expression and Activity of TRPM8 in TPCs

**Figure 5:**
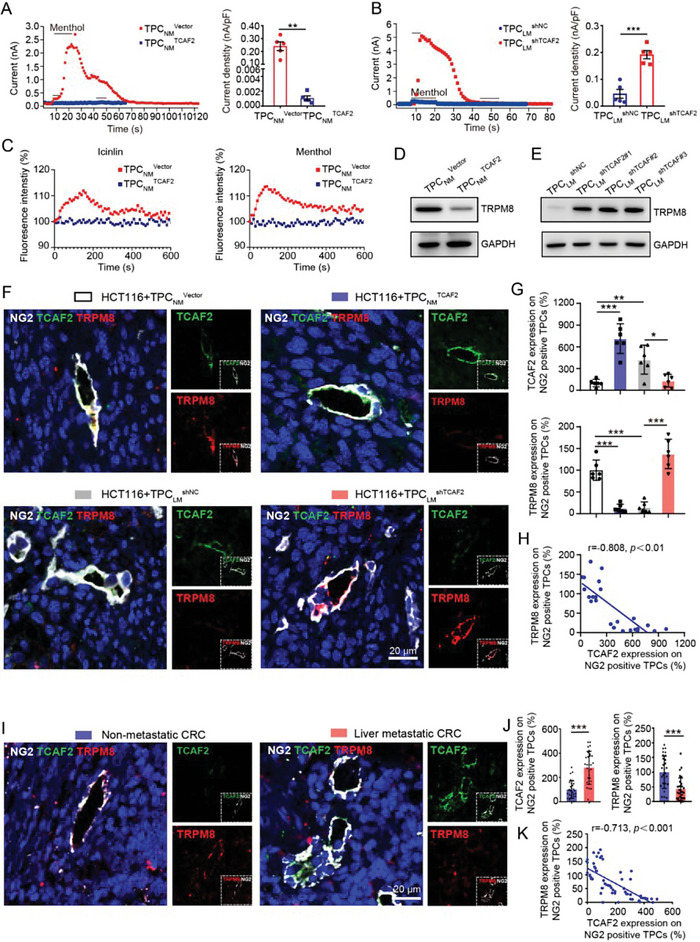
TCAF2 inhibits the expression and ion channel activity of TRPM8 in TPCs. A,B) Mean time course of menthol‐activated I_TRPM8_ in TCAF2‐overexpressing or ‐knockdown TPCs (n = 3). Quantification of the maximal value of I_TRPM8_ is shown. C) Intracellular Ca^2+^ change in response to menthol (500 µm) or icilin (100 µm) treatment of TPCs (n = 3). D,E) Western blot analysis of TRPM8 in TCAF2‐overexpressing (D) or ‐knockdown (E) TPCs. F) Immunofluorescence staining of TCAF2 (green) and TRPM8 (red) expression in TPCs (NG2, gray) in primary tumor sections derived from CRCLM xenografts (n = 6). Scale bar, 20 µm. G) Quantification of TCAF2 and TRPM8 expression in NG2^+^ TPCs. H) Pearson's correlation analysis for TRPM8 and TCAF2 in NG2^+^ TPCs. I) Representative images of TCAF2 (green) and TRPM8 (red) colocalization in TPCs (NG2, gray) in primary tumor sections derived from CRC patients with or without liver metastasis (n = 30). Scale bar, 20 µm. J) Quantification of TCAF2 and TRPM8 expression in NG2^+^ TPCs. K) Pearson's correlation analysis for TRPM8 and TCAF2 in NG2^+^ TPCs. Data are presented as mean ± SEM. *p < 0.05, **p < 0.01, ***p < 0.001 by two‐tailed unpaired *t*‐test in (A,B); by one‐way ANOVA followed by Tukey's post hoc test in (G); by Mann–Whitney *U* test in (J).

Given that TCAF2 is a binding factor for TRPM8 and can inhibit TRPM8 channel activation in tumor cells,^[^ ^20^ ^]^ we further evaluated whether TCAF2 exerted similar effects in TPCs. Icilin and menthol, two TRPM8‐specific agonists,^[^ ^38^ ^]^ were used to suppress the effect of TCAF2 on the ion channel activity of TRPM8 in TPCs. The menthol‐evoked TRPM8 ion channel activity in TPC_NM_^TCAF2^ was markedly lower than that in TPC_NM_^Vector^ (**Figure** **5**A). In contrast, TCFA2‐knockdown in TPC_LM_ significantly enhanced the menthol‐evoked I_TRPM8_ currents (Figure 5B). Consistently, Fluo‐4‐AM assay showed that overexpression of TCAF2 in TPC_NM_ significantly inhibited icilin‐ or menthol‐induced TRPM8‐associated extracellular calcium influx (Figure 5C). Moreover, TCAF2‐overexpression in TPCs inhibited the expression of TRPM8, whereas TCAF2‐knockdown in TPCs showed the opposite effects (Figure 5D,E). Immunofluorescence analysis validated that the expression of TRPM8 in NG2^+^ TPCs was reduced in the group co‐injected with HCT116 cells and TPC_NM_^TCAF2^ compared to that in the group co‐injected with TPC_NM_^Vector^, whereas co‐injection with TPC_LM_^shTCAF2^ increased the expression of TRPM8 in NG2^+^ TPCs compared to that in the group co‐injected with TPC_LM_^shNC^ (Figure 5F,G). Pearson's correlation analysis indicated that the expression of TRPM8 was negatively correlated with the expression of TCAF2 in NG2^+^ TPCs of tumor xenografts (Figure 5H). Further investigation showed that TRPM8 expression was lower in TPCs from primary tumor tissues derived from CRC patients with liver metastasis than that in TPCs from patients without metastasis (Figure 5I,J). Moreover, the expression of TRPM8 was negatively correlated with the level of TCAF2 in NG2^+^ TPCs (Figure 5K), indicating that TCAF2 could suppress the expression of TRPM8 in TPCs.

### 2.6 Activation of TRPM8 Suppresses CRCLM through Inhibiting Wnt5a Secretion

**Figure 6:**
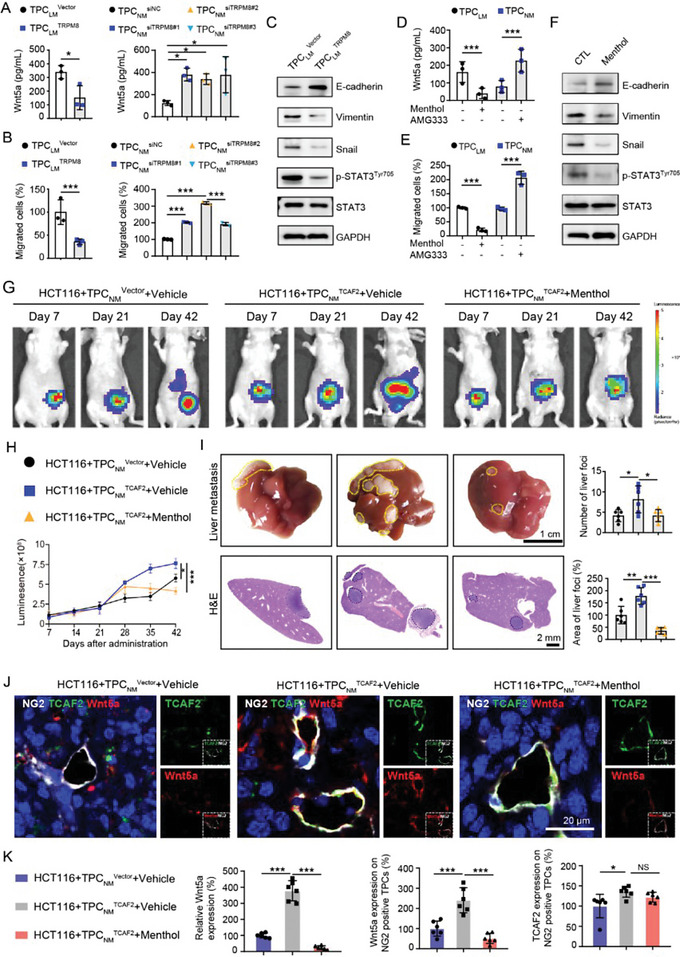
Activation of TRPM8 in TPCs suppresses Wnt5a secretion and CRCLM. A) ELISA analysis of Wnt5a secretion in the culture medium of TRPM8‐overexpressing or ‐knockdown TPCs (n = 3). B) Transwell assay for the migration of HCT116 cells primed with the conditioned medium from TRPM8‐overexpressing or ‐knockdown TPCs (n = 3). C) Western blot analysis for p‐STAT3^Tyr705^, STAT3, and EMT markers in HCT116 cells primed with the conditioned medium of TRPM8‐overexpressing TPC_LM_. D) ELISA assay for Wnt5a secretion in the culture medium of TPCs treated with menthol (100 µm) or AMG333 (40 µm) (n = 3). E) Transwell assay for the migration of HCT116 cells primed with the conditioned medium from TPCs pre‐treated with or without menthol (100 µm) or AMG333 (40 µm) (n = 3). F) Western blot analysis for EMT markers, STAT3, and p‐STAT3^Tyr705^ in HCT116 cells primed with the conditioned medium from TPCs pre‐treated with menthol (100 µm). G,H) Representative images and quantification of bioluminescence signals in primary tumor sections (n = 6). HCT116 cells were co‐injected with either TPC_NM_^Vector^ or TPC_NM_^TCAF2^ into the cecum wall of nude mice. Three weeks later (Day 21), mice injected with HCT116 cells and TPC_NM_^TCAF2^ were randomly divided into vehicle and menthol (80 mg kg^−1^, ip) groups. I) Representative images and quantification of liver metastases derived from CRCLM xenografts treated with or without menthol (n = 6). Yellow and black dotted lines indicate the liver metastatic foci. Scale bar, 1 cm (up); 2 mm (down). J) Immunofluorescence analysis for the colocalization of TCAF2 (green) and Wnt5a (red) in TPCs (NG2, gray) in primary tumor sections derived from CRCLM xenografts treated with or without menthol (n = 6). Scale bar, 20 µm. (K) Quantification for TCAF2 and Wnt5a expression in NG2^+^ TPCs and tumors. Data are presented as mean ± SEM. NS, *p < 0.05, **p < 0.01, ***p < 0.001 by two‐tailed unpaired *t*‐test in (A) (left), (B) (left), (D,E); by one‐way ANOVA followed by Tukey's post hoc test in (A) (right), (B) (right), (H,K).

Since TCAF2 inhibited ion channel activity and expression of TRPM8, we further investigated whether TCAF2 induced Wnt5a expression and tumor metastasis via TRPM8. We found that Wnt5a expression and secretion were negatively regulated by TRPM8. TPC_LM_ with TRPM8‐overexpression inhibited Wnt5a production, whereas knockdown of TRPM8 in TPC_NM_ inhibited Wnt5a expression and secretion (**Figure** **6**A; Figure S24A,B, Supporting Information). The conditional medium of TPC_NM_^siTRPM8^ facilitated the migration of HCT116 and DLD‐1 cells compared with those in the group treated with TPC_NM_^siNC^ medium, and the conditioned medium of TPC_LM_^TRPM8^ significantly suppressed the migration of HCT116 and DLD‐1 cells compared with those treated with TPC_LM_^Vector^ medium (Figure 6B; Figure S24C,D, Supporting Information). Additionally, the medium of TPC_NM_^siTRPM8^ induced EMT in HCT116 and DLD‐1 cells, as indicated by the increased expression of Vimentin, Snail, and p‐STAT3^Tyr705^, and decreased expression of E‐cadherin compared to TPC_NM_^siNC^, whereas the conditional medium of TPC_LM_^TRPM8^ showed opposite effects compared to TPC_LM_^Vector^ (Figure 6C; Figure S24E,F, Supporting Information).

Next, we investigated the effect of TRPM8 ion channel activity on tumor cell motility. Following treatment with TPC_LM_ and TPC_NM_ by the agonists (menthol and icilin) or antagonist (AMG‐333), the conditional medium of TPCs was used to prime HCT116 and DLD‐1 cells. Our results showed that menthol and icilin suppressed the expression and secretion of Wnt5a, whereas AMG‐333 exerted the opposite effects (Figure 6D; Figure S24G,H, Supporting Information). Similarly, activation of TRPM8 ion channel activity by menthol and icilin significantly inhibited the migration of HCT116 and DLD‐1 cells (Figure 6E; Figure S24I,J, Supporting Information), accompanied by decreased expression of Vimentin, Snail, and p‐STAT3^Tyr705^, and increased level of E‐cadherin (Figure 6F; Figure S24K,L, Supporting Information). However, AMG333 treatment promoted the migration, EMT, and activation of the Wnt5a/STAT3 axis in CRC cells (Figure 6E; Figure S24J,M, Supporting Information).

To verify the effects of TRPM8 ion channel activity in TPCs on CRCLM in vivo, HCT116 cells were co‐injected with either TPC_NM_^Vector^ or TPC_NM_^TCAF2^ in the cecum wall of nude mice. Menthol significantly suppressed TPC_NM_^TCAF2^‐induced CRCLM and EMT, as indicated by the decreased liver metastatic foci (Figure 6G–I), downregulated expression of N‐cadherin and Vimentin, and increased E‐cadherin level in primary tumors (Figure S25, Supporting Information). Additionally, menthol suppressed the expression of Wnt5a and phosphorylation of STAT3 in primary tumors compared with those in the vehicle group (Figure 6J,K; Figure S25, Supporting Information). Interestingly, menthol had a negligible effect on TCAF2 expression in NG2^+^ TPCs in primary tumors (Figure 6J,K). Collectively, these data indicate that TCAF2‐induced Wnt5a secretion through inhibiting TRPM8 in TPCs, which activates the STAT3 signaling pathway in tumor cells, thus facilitating CRCLM.

## 3 Discussion

As gatekeepers of tumor vessels, TPCs play an important role in monitoring tumor hematogenous metastasis. Current approaches, including MACS and FACS, cannot obtain TPCs with high purity, and the sorted cells are subjected to mechanical stress during dissociation, which does not meet the requirements for multi‐omics and functional studies. In this study, we developed an MPMA for the isolation of TPCs. Compared to previously reported methods, MPMA had the following advantages. First, this non‐enzymatic and non‐mechanical method caused negligible damage to cells, thus avoiding the influence of mechanical force on the cell transcriptome.^[^ ^39^ ^]^ Second, this method maximally preserves the heterogeneity of TPCs, which was conducive to scRNA‐seq.^[^ ^25^, ^40^ ^]^ However, once the MPMA‐obtained TPCs were isolated from tissues, they may lose their in vivo features, such as interaction with ECs, thus exhibiting mesenchymal profiles and acquiring fibroblast‐like phenotypic properties (Figure S26, Supporting Information), which did not fully reflect the features in vivo. Nevertheless, the MPMA‐obtained TPCs retained part of the primary pericyte characteristics similar to those in vivo (Figure S27, Supporting Information), indicating that MPMA may be an alternative method for obtaining TPCs used for functional experiments.

The role of Wnt5a in cancer progression remains controversial and may be dependent on the cell type.^[^ ^41^, ^42^ ^]^ Wnt5a is elevated in metastatic liver, lung, colon, and breast cancer cell lines and its expression is correlated with tumor cell EMT.^[^ ^37^ ^]^ Consistent with a previous study,^[^ ^43^ ^]^ we found that Wnt5a secretion from TPCs induced the migration and EMT of CRC cells, thus promoting CRCLM. Multiple processes, including tumor cell EMT, intravasation, formation of CTCs, extravasation, homing, seeding, and colonization, are involved in distant tumor metastasis.^[^ ^44^ ^]^ Among these, EMT in tumor cells has been shown to initiate tumor metastasis.^[^ ^45^ ^]^ Pericytes attached to the perivascular wall play an important role in tumor metastasis during tumor cell intravasation and extravasation.^[^ ^14^, ^46^ ^]^ However, the regulatory effects of TPCs on tumor metastasis remain controversial. TPCs act as a physiological barrier to limit tumor cell intravasation,^[^ ^47^ ^]^ whereas several TPC subsets, such as CD45^−^VLA‐1^bri^, CD248^+^, and TCF21^high^ TPCs, exert pro‐metastatic effects by promoting tumor cell intravasation.^[^ ^14^, ^25^, ^48^ ^]^ These contradictory effects of TPCs on tumor metastasis may be associated with their heterogeneity.^[^ ^49^ ^]^ Here, we found that TCAF2 was highly expressed in TPCs from primary tumors, but not in TPCs derived from liver metastatic tumors, which directly facilitated tumor cell EMT, migration, and subsequent intravasation at the initiation of tumor metastasis. Nevertheless, the indirect effects of pericyte‐TCAF2 on tumor cell extravasation, seeding, and colonization may have resulted from an increased number of CTCs. Additionally, the level of TCAF2 in TCF21^high^ TPCs was evaluated by scRNA‐seq analysis.^[^ ^25^ ^]^ The results showed that TCAF2 was highly expressed in multiple TPC subpopulations derived from CRC patients, including TCF21^high^ TPCs (Figure S28A, Supporting Information). However, genetic manipulation of TCF21 had negligible effects on TCAF2 expression in TPCs (Figure S28B, Supporting Information), indicating the expression of TCAF2 in TPCs was not regulated by TCF21, which is reasonable cause TCAF2 in TPCs exerts a distinct function compared to the TCF21^high^ TPCs. Taken together, this study provides a novel metastasis‐associated TPC subpopulation, expanding the understanding of the heterogeneity of TPCs.

The discovery of valid biomarkers is of significance for the diagnosis and treatment of CRCLM. Multiple biomarkers have been established to predict or diagnose CRCLM, such as carcinoembryonic antigens in the serum,^[^ ^50^ ^]^ CTCs,^[^ ^51^ ^]^ and circulating tumor DNA (ctDNA).^[^ ^52^ ^]^ However, these methods do not fully meet the requirements for the diagnosis of CRC metastasis at an early stage. CRC cells remodel tumor vessels in primary tumors before tumor cell intravasation, resulting in the formation of CTCs and metastatic seeds.^[^ ^53^ ^]^ Given that intravasation is an early step in hematogenous tumor metastasis, changes in the molecular characteristics of TPCs should occur before the detection of CTCs or ctDNA. Therefore, it is of vital clinical significance to identify early diagnostic and predictive markers for CRCLM in TPCs. The tumor vessels tended to destabilize with increased MVD and dilated vascular size (Figure S29A, Supporting Information) and decreased pericyte coverage (Figure S29B, Supporting Information) and an incomplete basement membrane (Figure S29C, Supporting Information) in tumor metastasis, which may be resulted from the detachment of pericytes due to pericyte‐fibroblast transition.^[^ ^8^ ^]^ However, TCAF2 in TPCs had negligible effects on the morphology and function of tumor vessels, indicating that TCAF2 could serve as an early diagnostic marker for hematogenous tumor metastasis.

The function of ion channel activity in PCs has been widely studied in cardiovascular, cerebrovascular, and retinal vascular diseases.^[^ ^54^ ^]^ However, the expression and key regulatory effects of ion channel proteins and their partner proteins on TPCs and tumor development remain unknown. TRPM8 is the primary cold sensor that can be activated by innocuous cooling (<28 °C) and cooling agents such as menthol and icilin,^[^ ^18^ ^]^ which is highly expressed in multiple malignant tumors, including prostate, lung, and colorectal cancers, and is involved in tumor proliferation, survival, and invasion.^[^ ^19^ ^]^ TCAF2 is a partner protein of TRPM8, which binds to the N‐terminal tail of TRPM8^[^ ^20^ ^]^ and promotes TRPM8 trafficking to the cell surface to gate the ion channel.^[^ ^55^ ^]^ However, the exact binding site between TCAF2 and TRPM8 is currently unknown. Exploring the binding site of TCAF2 and TRPM8 would contribute to the development of inhibitors blocking the binding of TCAF2 with TRPM8, as well as to the TPC‐targeting strategy that suppresses tumor hematogenous metastasis through hindering the interaction of TCAF2 and TRPM8 in TPCs. Our study revealed the pro‐metastatic effects of TCAF2 and TRPM8 in TPCs from a new perspective on ion channels, providing a potential target for inhibiting tumor metastasis. Furthermore, the protein structures of human TCAF2 and TRPM8, as well as the binding sites between human TCAF2 and TRPM8 should be further investigated with X‐ray crystallography and cryoelectron microscopy.

## 4 Conclusion

In conclusion, this study provides an alternative protocol to isolate TPCs from solid tumors. More importantly, this study first reveals the function and mechanisms of cold‐sensing receptors and their associated factor in TPCs during hematogenous metastasis, which uncovers a potential TPC‐related diagnostic and therapeutic target for hematogenous tumor metastasis.

## 5 Experimental Section

### Cell Lines and Cell Culture

The human CRC cell lines HCT116 (Cat. CCL‐247), DLD‐1 (Cat. CCL‐221), LoVo (Cat. CCL‐229), SW480 (Cat. CCL‐228), SW620 (Cat. CCL‐227), HT‐29 (Cat. HTB‐38), WiDr (Cat. CCL‐218), and human dermal microvascular endothelial cells (HMEC‐1, Cat. CRL‐3243) were purchased from American Type Culture Collection (Manassas, Virginia, USA). The mouse CRC cell line (MC38) was purchased from BeNa Culture Collection (Beijing, China). HCT116, DLD‐1, LoVo, SW480, SW620, HT‐29, WiDr, and MC38 cell lines were cultured in DMEM (Cat. 11965092; Gibco, Waltham, MA, USA) supplemented with 10% FBS (Cat. FCS500, Excell Bio, Shanghai, China) and 1% penicillin–streptomycin (PS, Cat. 15140122, Gibco). HMEC‐1 cells were cultured in endothelial cell medium (ECM) supplemented with 5% FBS, 1% endothelial cell growth supplement (ECGS), and 1% PS (Cat. 1001, ScienCell, Corte Del Cedro Carlsbad, CA). The TPCs and NG2^+^ cells were cultured in pericyte medium (PM) supplemented with 2% FBS, 1% PGS, and 1% PS (Cat. 1201, ScienCell). All the cells were maintained in a humidified incubator at 37 °C with 5% CO_2_. MC38, HCT116, and DLD‐1 cells were infected with lentivirus‐harboring luciferase (Genechem, Shanghai, China) to generate luciferase‐labeled cells (MC38‐luc, HCT116‐luc, and DLD‐1‐luc), which were then selected using puromycin (2 µg mL^−1^) for 2 days. All cell lines were authenticated using the Short Tandem Repeat (STR) Multi‐amplification Kit (Microreader 21 ID System) and tested negative for mycoplasma using the Mycoplasma Detection Set (M&C Gene Technology, Beijing, China). For hypoxia studies, TPCs at 60% confluency were cultured in a sealed hypoxia chamber filled with 5% CO_2_, 1% O_2_, and 94% N_2_ at 37 °C for 24 h. The treated cells were then collected for further experiments.

### Animals

Male BALB/c nude mice (6–8 weeks, weight 20–22 g), male C57BL/6JGpt mice (6–8 weeks, weight 24–26 g), male NOD/SCID mice (6–8 weeks, weight 24–26 g), Cspg4‐CreERT2 mice (B6/JGpt‐Cspg4em1Cin(CreERT2‐P2A)/Gpt; T006187) were obtained from GemPharmatech Co., Ltd. (Nanjing, China). Adeno‐Associated virus (AAV) was administered as previously described.^[^ ^56^ ^]^ Briefly, 5 × 10^10^ AAV particles were dissolved in 200 µL PBS. 6‐week old Cspg4‐CreERT2 mice were intravenously (i.v.) injected with AAV‐CTR or AAV‐TCAF2 (Genechem, Shanghai, China) to construct pericyte‐*Tcaf2* conditional knockout mice. All the mice were maintained in a specific pathogen‐free (SPF) facility. All animal experiments were approved by the Experimental Animal Ethics Committee of Jinan University (Approval number: 00287194) and complied with ARRIVE guidelines, which were carried out in accordance with the National Institutes of Health Guide for the Care and Use of Laboratory Animals (NIH Publication No. 8023, revised 1978).

### Human Specimens

Surgically resected tumor samples for TPC isolation (12 cases, patients’ information is listed in Tables S4 and S5, Supporting Information), for patient‐derived tumor xenograft (PDX) model (two cases, patients’ information is listed in Table S6, Supporting Information), and specimens derived from primary tumors of CRC patients with or without liver metastases (93 cases, patients’ information is listed in Table S7, Supporting Information) were obtained from the First Affiliated Hospital of Jinan University. The primary tumor specimens derived from breast cancer patients with or without pulmonary metastasis were obtained from the First Affiliated Hospital of Jinan University (20 cases, patients’ information is listed in Table S8, Supporting Information). The human tissues and specimens used in this study were approved by the Clinical Ethics Committee of the First Affiliated Hospital of Jinan University, and written informed consent was received from participants prior to inclusion in the study (Approval number: JNUKY‐2023‐0067).

### Isolation and Culture of TPCs from Human CRC Tissues by MPMA

Freshly resected human primary tumor tissues (12 cases) were placed in ice‐cold PS contained DMEM and transported to the laboratory within 1 h. Following washing with pre‐cooled washing buffer (PBS with 0.1% gentamicin, 0.1% ciprofloxacin, and 0.1% kanamycin) in a sterile dissection hood, fibrous, and adipose tissues around the muscular layers of tumor tissues were carefully removed. The remaining tissues were cut into small pieces along the colon mucosa into the muscular layer, followed by fastening in a sylgard‐coated petri dish using ice‐cold PBS and a surgical needle. For TPC collection, ascending capillaries (diameter ≈ 20 µm) derived from arterioles were gently separated along the mucosa into the submucosa from perivascular adipose tissues using eye scissors (Geuder, G‐19745) under a stereomicroscope (SZX7; Olympus). The attached adipose tissues were then removed, and the dissected capillaries were transferred to fresh dishes containing PM. The TPCs migrated from the vascular samples to the culture plate within 14 days. When the cells reached 80% confluence, they were dissociated using trypsin, collected, and transferred to a 60 mm‐diameter culture dish. The culture medium was gently replaced every two days. The purity of the isolated TPCs was confirmed using FACSort and CellQuest software (BD Bioscience), authenticated using STR Multi‐amplification Kit, and tested negative for mycoplasma using the Mycoplasma Detection Set. TPCs isolated from CRC patients with and without liver metastases were termed TPC_LM_ and TPC_NM_, respectively.

### Statistics

All experiments were independently repeated three times. Data were presented as the mean ± SEM, and statistical analysis was performed using GraphPad Prism 8.0 (GraphPad Software, Inc., San Diego, CA, USA). Differences between the two groups were evaluated using a two‐tailed unpaired *t*‐test or Mann–Whitney *U* test, and differences among the three groups were evaluated using one‐way ANOVA followed by Tukey's post hoc test. Survival curves were plotted using the Kaplan–Meier method and compared using the log‐rank test. To evaluate diagnostic accuracy, ROC curves and areas under the curve (AUC) were generated and calculated using a logistic regression model. Comparisons of variables were performed using Fisher's exact test or the chi‐squared test based on categorical data. *p* < 0.05 was considered significantly different.

## Conflict of Interest

The authors declare no conflict of interest.

## Author Contributions

X.L., and Q.Q. contributed equally to this work. D.Z., W.Y., and M.C. designed and supervised the experiments, and revised the manuscript. Q.Q., T.L., and L.D. revised the manuscript critically. M.C., X.L., and Y.L. wrote the manuscript and analyzed the data. X.L., M.C., Y.L., Q.M., M.Q., W.Y., F.Y., X.C., M.H., C.W., S.F., Z.Z., W.D., and X.Z. performed experiments. J.P., Z.Z., D.H., S.Q, and Y.Z. collected human CRC tissues, reviewed the pathological sections, and assessed preclinical and clinical samples. The order of the co‐first authors was determined based on their contributions to the manuscript. All authors have reviewed and approved the final version of the manuscript.

## Supporting information

Supporting Information

## Data Availability

The raw mass spectrometry proteomics data were deposited in the Proteome Xchange Consortium via the PRIDE partner repository with the dataset identifiers PXD032935 and PXD033011. Raw RNA‐seq data were deposited in the NCBIs Gene Expression Omnibus and are accessible through the GEO Series accession number GSE216780. Single cell‐RNA sequencing (scRNA‐seq) data of TPCs directly from CRC patients acquired using the 10× Chromium protocol were downloaded from GEO accession GSE178341. scRNA‐seq data of the MPMA‐obtained TPCs acquired using the 10× Chromium protocol were downloaded from GEO accession GSE199726, and the sequencing reads were realigned and analyzed as previously described.
