# Supplementary material for: TCAF2 in Pericytes Promotes Colorectal Cancer Liver Metastasis via Inhibiting Cold‐Sensing TRPM8 Channel
Source: Adv Sci (Weinh). 2023 Aug 27;10(30):2302717. doi: 10.1002/advs.202302717 (PMC10602580; doi:10.1002/advs.202302717)

ADVANCED SCIENCE
Open Access

Supporting Information

for *Adv. Sci.*, DOI 10.1002/advs.202302717

TCAF2 in Pericytes Promotes Colorectal Cancer Liver Metastasis via Inhibiting Cold-Sensing TRPM8 Channel

Xiaobo Li, Qi Qi, Yong Li, Qun Miao, Wenqian Yin, Jinghua Pan, Zhan Zhao, Xiaoying Chen, Fan Yang, Xiaofeng Zhou, Maohua Huang, Chenran Wang, Lijuan Deng, Dandan Huang, Ming Qi, Shuran Fan, Yiran Zhang, Shenghui Qiu, Weiqing Deng, Tongzheng Liu, Minfeng Chen, Wencai Ye* and Dongmei Zhang**

Supporting Information

TCAF2 in pericytes promotes colorectal cancer liver metastasis via inhibiting cold-sensing TRPM8 channel

Xiaobo Li, Qi Qi, Yong Li, Qun Miao, Wenqian Yin, Jinghua Pan, Zhan Zhao, Xiaoying Chen, Fan Yang, Xiaofeng Zhou, Maohua Huang, Chenran Wang, Lijuan Deng, Dandan Huang, Ming Qi, Shuran Fan, Yiran Zhang, Shenghui Qiu, Weiqing Deng, Tongzheng Liu, Minfeng Chen, Wencai Ye*, Dongmei Zhang**

Supplementary Methods

Isolation and culture of NG2^{+} cells from human CRC tissues by MACS: Fresh tumor tissues were resected from patients with CRC and immediately placed in ice-cold DMEM containing PS. Following washing with pre-cooled washing buffer (PBS with 0.1% gentamicin, 0.1% ciprofloxacin, and 0.1% kanamycin) in a sterile dissection hood, fibrous and adipose tissues around the muscular layers of the tumor tissues were carefully removed. The remaining tissues were cut into small pieces and incubated with type I collagenase (Cat. A004194; Sangon Biotech, Shanghai, China), and type II collagenase (Cat. A004174, Sangon Biotech) at 37 °C for 60 min. Filtered single-cell suspensions were collected by centrifugation at 700 × *g* for 10 min. The resulting cells were collected using anti-NG2 microbeads (Cat. 130-097-170, Miltenyi Biotec, Shanghai, China). The purity of the isolated NG2^{+} cells was determined using FACSort and CellQuest software, and authenticated using the STR Multi-amplification Kit.

In vivo experiments: For pericyte-*Tcaf2* deletion experiments, *Cspg4*-Cre mice pre-administered AAV-CTR or AAV-*Tcaf2* were anesthetized with isoflurane, and then MC38-luc cells (1×10^5 cells suspended in 100 µL of Matrigel) were injected into the cecum wall of AAV-loaded mice. Cre activity was induced via oral gavage of tamoxifen (10 mg kg^{-1} of tamoxifen in peanut oil) every other day for 3 times. Tumor metastasis was monitored using an *in vivo* cell tracking system with Xenogen IVIS 200 (Alameda, CA, USA). At the end of experiment, mice were euthanized, and tumor and liver tissues were collected and subjected to immunohistochemistry and immune-fluorescence analyses.

For the co-injection assay, HCT116-luc, DLD-1-luc, TPC_{NM}^{Vector}, TPC_{NM}^{TCAF2}, TPC_{LM}^{shNC} or $TPC_{LM}^{shTCAF2}$ were harvested and counted. HCT116-luc cells (4×10^5) or DLD-1-luc cells (4×10^5) were mixed with TPCs (1.6×10^6) and supplemented with 200 µL PBS. The mixed cells were injected into the cecum serosa of nude mice. Mouse liver metastases were detected using an *in vivo* cell-tracking system. Five weeks later, the mice were euthanized, and primary tumor tissues and liver metastatic foci were obtained and analyzed by hematoxylin and eosin (H&E) staining, immunohistochemical staining, and immunofluorescence analysis.

For menthol treatment experiments, HCT116-luc cells were co-injected with either TPC_{NM}^{Vector} or TPC_{NM}^{TCAF2} into the cecum wall of nude mice. Three weeks later, mice injected with HCT116-luc cells and TPC_{NM}^{TCAF2} were randomly divided into the vehicle and menthol groups. Mice in the vehicle or menthol group were intraperitoneally injected with 1% DMSO-containing saline or 80 mg kg^{-1} menthol every other day for three weeks. Metastatic progression was monitored and evaluated by detecting bioluminescence signals. At the end of experiments, tumor and liver tissues were collected for further analysis.

For the patient-derived tumor xenograft (PDX) model, fresh surgically resected primary tumor tissues were obtained and placed in ice-cold DMEM supplemented with 1% penicillin-streptomycin (PS). The tissues were then minced into 3- to 5-mm^3 fragments, followed by treatment with a Tumor Dissociation Kit (Cat. 130-095-929, Miltenyi Biotec). The resulting cells were counted, and $4×10^5$ cells were mixed with either TPC_{NM} or TPC_{LM} ($1.6×10^6$) and co-injected into the cecum of NOD/SCID mice. Five weeks later, the mice were euthanized, and primary tumor tissues and liver metastatic foci were acquired and analyzed by H&E and immunohistochemical staining.

All animal experiments were approved by the Experimental Animal Ethics Committee of Jinan University (Approval number: 00287194) and complied with ARRIVE guidelines, which were carried out in accordance with the National Institutes of Health Guide for the Care and Use of Laboratory Animals (NIH Publication No. 8023, revised 1978).

Transmission electron microscopy: CRC tissues were fixed with 2.5% glutaraldehyde for 4 h at 4 °C followed by osmication with 1% osmium tetroxide for 2 h at room temperature. The fixed samples were stained with 3% uranyl acetate, dehydrated using graded concentrations of ethanol, and embedded in low-viscosity resin. The embedded samples were sectioned using a UCT ultramicrotome and diamond knife. For TPCs, the samples were fixed with 2.5% glutaraldehyde for 1 h, collected, and centrifuged for 10 min at $1×10^4$ rpm at room temperature. Cell pellets were embedded in agarose and sectioned. Sections of either tissues or cells were mounted on homemade EM grids with a plastic carbon support film and stained with saturated uranyl acetate and lead citrate. The sections were imaged using a JEM1200EX

transmission electron microscope at 80 kV equipped with a BioScan600W digital camera (JEOL, Tokyo, Japan).

Flow cytometry: Cells were collected, re-suspended in flow cytometry staining buffer, and distributed into 1.5 mL EP tubes. Following fixation with 4% paraformaldehyde on ice and permeabilization with 0.1% Triton X-100 in PBS for 5 min, the cells were incubated with the indicated Alexa Fluor-conjugated antibodies for 1 h on ice and analyzed using flow cytometry (BD Biosciences, San Jose, CA). The antibodies used are listed in **Table S9**.

Histology, immunohistochemistry, and immunofluorescence: Formalin-fixed tissue samples were embedded in paraffin and sectioned at 5 μm thickness. H&E staining was performed according to standard procedures. For immunohistochemical staining, the sections were deparaffinized, rehydrated, antigen retrieved by boiling with sodium citrate antigenic repair solution (pH 9.0), and blocked with 3% BSA solution. This was followed by incubation with primary antibodies overnight at 4 °C. The slides were then incubated with horseradish peroxidase (HRP)-conjugated secondary antibodies and visualized using a DAB kit (Cat. G1212, Servicebio, Wuhan, China). Images were analyzed using an Olympus BX53 inverted epifluorescence microscope (Olympus, Japan). For tissue immunofluorescence staining, the slides incubated with primary antibodies were washed and incubated with HRP-conjugated secondary antibodies for 1 h at room temperature. The slides were then washed and visualized with iF488-Tyramide, iF555-Tyramide, or iF647-Tyramide using TSAPLus Fluorescence Kits (Cat. G1236, Servicebio), and mounted with a DAPI-containing anti-fluorescent quenching sealant. Images were obtained and analyzed using a Zeiss LSM 800 confocal

microscope (Zeiss, Germany). Five areas from each section were blindly selected to count the percentage of positively stained cells, and the mean extent of staining was calculated using Image-Pro Plus 6.0. For cell immunofluorescence staining, the cells were seeded on confocal Petri dishes, fixed with 4% paraformaldehyde for 20 min, and permeabilized in 0.2% TritonX-100 for 5 min. The confocal Petri dishes were then subjected to blocking and staining, and examined using a Zeiss LSM 800 confocal microscope. Positive cells were counted at 400-fold magnification. The antibodies used for immunohistochemistry and immunofluorescence are listed in **Table S10**.

TCAF2$^+$ TPC ratio (%) = counts of TCAF2$^+$ TPCs /counts of total TPCs per vessel × 100%. TPCs were identified by NG2 staining around CD31$^+$ endothelial cells in the tumor tissue, and TCAF2$^+$ TPCs were TCAF2$^+$/NG2$^+$ cells around CD31$^+$ endothelial cells. Five vessels were randomly selected from each CRC specimen. TCAF2$^+$ TPC ratio for each patient (%) = sum of TCAF2$^+$ TPC ratio/5. In this study, clinical specimens were obtained from 93 CRC patients and the pericyte ratio was evaluated by immunofluorescence analysis.

Vessel permeability assay: Mice bearing MC38-luc orthotopic xenografts were intravenously (i.v.) injected with 1 mg of FITC-labeled Dextran-40 kDa (Cat. D1844, Thermo Scientific) for 10 min. The mice were then perfused with 4% PFA, and the tumors were acquired and frozen. Tumor tissues were sectioned at a thickness of 5 μm, and tumor vessels were stained for CD31, followed by incubation with donkey anti-goat IgG (H+L) Cross-Adsorbed Secondary Antibody, Alexa Fluor™ 647. Double staining of CD31$^+$ vessels with FITC-labeled dextran indicated vessel permeability.

Western blot assay: Cells were lysed with radioimmunoprecipitation assay (RIPA) buffer supplemented with protease and phosphatase inhibitors for 30 min on ice. Cell lysates were centrifuged for 15 min at 1.2×10^4 rpm at 4 °C. The total protein concentration was determined using the Pierce BCA Protein Assay Kit (Cat. 23225, Thermo Scientific, Waltham). Equivalent amounts of protein were separated on 10% SDS polyacrylamide gels and transferred to PVDF membranes. The membranes were blocked with 5% BSA dissolved in TBST for 1 h and then incubated with primary antibodies overnight at 4 °C. After washing, membranes were incubated with HRP-conjugated secondary antibodies for 1 h at room temperature (The information of antibodies is listed in **Table S11**). Immunoreactive bands were visualized using the Clarity^{TM} Western ECL substrate (Cat. 1705060, Bio-Rad, Hercules, CA). Gray values for each band were measured using ImageJ software.

Quantitative polymerase chain reaction (qPCR) assay: Total RNA was extracted using E.Z.N.A.® Total RNA Kit I (Cat. R6834-02, Omega Bio-Tek, Norcross, GA). Briefly, the cells were harvested and lysed in TRK lysis buffer on ice. Cell lysis under denaturing conditions can inactivate RNases and prevent RNA degradation. The cell lysates were loaded into an RNA homogenizer spin column and centrifuged at 1.2×10^4 g for 2 min to collect the homogenized lysate. The collected cell lysates were mixed with 70% ethanol at a volume ratio of 1:1 and loaded into a Hibind RNA mini column. The column was centrifuged at 1.0×10^4 g for 1 min, and the filtrate was discarded. Then, the column was washed twice with RNA wash buffer I and RNA wash buffer II and centrifuged at 1.2×10^4 g for 2 min. After washing, the column was transferred to a clean 1.5 mL microcentrifuge tube, and 30 μL of nuclease-free water was loaded for elution. The purity and concentration of the RNA were examined using a Nanodrop Lite micro

spectrophotometer. Equal amounts of total RNA (2 μg) were reversely transcribed into complementary DNA (cDNA) using the SweScript RT I First Strand cDNA Synthesis Kit (Cat. G3330, Servicebio). Quantitative PCR analysis was conducted using 2× SYBR Green qPCR Master Mix (Cat. B21202, Selleck, Houston, TX). The relative mRNA levels were normalized to endogenous *ACTB* mRNA levels, and the results are presented as fold-changes compared to the control TPCs. The results were further analyzed using the $2^{-\Delta\Delta CT}$ method [$\Delta Ct = Ct_{target} - Ct_{ACTB}$ and Δ (ΔCt) = $\Delta Ct_{sample} - \Delta Ct_{control}$]. Primers used are listed in **Table S12**.

Tandem mass tag (TMT) relative quantitative proteomic analysis: TMT relative quantitative proteomic analysis was performed as previously described, with some modifications [1]. Briefly, TPCs (n = 3 each) were homogenized in SDT buffer (4%SDS, 100 mM Tris-HCl, pH 7.6, 0.1 M DTT) and the protein was quantified using the BCA protein assay kit. Then, 20 μg of protein from each sample was separated using SDS-PAGE and digested according to the FASP (filter-aided sample preparation) procedure. The digested peptides were labeled according to the instructions of the TMT labeling kit (Cat. A43073, Thermo Scientific), and TMT-labeled peptides from each sample were mixed in equal amounts and fractionated using high-pH reversed-phase liquid chromatography. For proteomic analysis, the fractionated peptides were loaded onto a loading column in 95% buffer A and separated using a nano-flow HPLC Easy-nLC system (Thermo Scientific,10 cm, ID 75 μm, 3 μm, C18-A2) at a flow rate of 300 nL min^{-1} [buffer A, 0.1% v/v formic acid in water; buffer B, 0.1% (v/v) formic acid in acetonitrile]. The samples were analyzed using a Q-Exactive mass spectrometer (Thermo Scientific). The mass spectrometry data were analyzed using Mascot version 2.2 and Proteome Discoverer version1.4, and searched against the UniProt database

(downloaded on December 12, 2014, including 76417 sequences). For quantitative analysis, the relevant parameters and descriptions were as follows: enzyme = trypsin; max missed cleavage = 2; fixed modification = carbamidomethyl (C); variable modifications = oxidation (M); peptide false discovery rate (FDR) ≤ 0.01. Protein ratios were calculated based only on the median of the unique peptides of the protein. Proteins with expression showing log2(fold change) > 1.5 and *P*-values < 0.05 were considered differentially expressed. Gene set enrichment analysis (GSEA) were performed using ClusterProfiler [2].

Collection of conditional medium: The cells were seeded in 6-well plates (1×10^5 cells/well) and cultured overnight. The cells were then starved in serum-free medium for 24 h, and the conditional medium was collected and filtered through a 0.2 μm syringe filter.

Cell migration assays: Cell migration with or without Matrigel assays were conducted in 24-well Boyden chambers (Corning, NY, USA) containing polycarbonate membrane inserts with 8 μm pores. For the cell migration without Matrigel assay, cells (2×10^4) in 100 μL serum-free medium were seeded in the upper chamber, and 600 μL of medium was added to the lower chamber. After 48 h of incubation, the upper chamber was washed twice with PBS, the cells were fixed with 4% paraformaldehyde at room temperature for 30 min, and then stained with 0.1% crystal violet. Subsequently, the non-migrated cells were removed from the chamber using cotton swabs, and the migrated cells were imaged using an Olympus BX 53 microscope and analyzed using the Image Pro Plus 6 software. For cell migration with Matrigel assay, the upper chamber was coated with 30 μL of diluted Matrigel (Cat. 354248, Corning, NY) and

incubated for 30 min at 37 °C. The rest of the procedures were the same as those of the migration assay without Matrigel.

For endothelial cell migration assay, HMEC-1 cells (2×10^4) were seeded in the upper chamber coated with or without diluted Matrigel, and then TCAF2-overexpressing or -knockdown TPCs (2×10^4) supplemented with 600 μL of PM were plated in the bottom chamber. After 48 h of co-culturing, the upper chamber was washed, fixed and stained as described above. The migrated cells were imaged using an Olympus BX 53 microscope and analyzed using the Image Pro Plus 6 software.

Capillary-like tube formation assay: $NG2^+$ cells, TPCs, or HMEC-1 cells were collected and re-suspended in serum-free medium (ECM:PM, 5:1). TPCs (5×10^3 cells) or $NG2^+$ cells (5×10^3 cells) were labeled with PKH-67 (Cat. MINI67, Sigma-Aldrich, St. Louis, MO, USA), and HMEC-1 cells (2×10^4 cells) were labeled with PKH-26 (Cat. MINI26, Sigma-Aldrich) for 3-5 min at room temperature. PKH-26 labeled HMEC-1 (red) were re-suspended in 100 μL serum-free medium and seeded in Matrigel-pre-coated 96-well plates. After 4 h of incubation, PKH-67 labeled TPCs (green) or $NG2^+$ cells (green) were added to the endothelial tubes. After 2 h of incubation, capillary-like tubes were photographed under a Zeiss HAL100 fluorescence microscope. The number of TPCs or $NG2^+$ cells attached to HMEC-1 cells-formed tubes was analyzed by the Image Pro Plus 6 software.

The Endothelial tube formation assay was performed as previously described with some modifications. [3] HMEC-1 cells were labeled with PKH26 and resuspended in ECM at a concentration of 2×10^5 cell mL^{-1}. TCAF2-knockdown or -overexpressing TPCs were labeled with PKH-67, and then co-cultured with HMEC-1 cells at a ratio of

1:10 on Matrigel. A total of 2×10^4 HMEC-1 cells per well were seeded onto a 96-well plate. After 4 h of incubation, capillary-like tubes were photographed under a Zeiss HAL100 fluorescence microscope. The number of tubes formed by HMEC-1 cells was analyzed by the Image Pro Plus 6 software.

Cell proliferation assay: For EdU assay, the cells (5×10^3 cells) were incubated overnight in 96-well plates. Cell proliferation was measured using the BeyoClick™ EdU Cell Proliferation Kit with Alexa Fluor 555 (Cat. C0075S, Beyotime, Shanghai, China). The results were analyzed using the Image Pro Plus 6 software. For the MTT assay, cells (1×10^3) were seeded into 96-well plates, and a total of 14 plates were prepared (Day 0). Cell viability was examined by the MTT assay every day for 14 days (Day 1-Day 14).

For the endothelial cell proliferation assay. HMEC-1 cells were co-cultured with TPCs using a 24-well Boyden chambers with 3 μm membrane pores. TCAF-overexpressing or -knockdown TPCs (5×10^4) were seeded in the upper chamber, while HMEC-1 cells (2×10^4) were plated in the bottom compartment and co-cultured for 24 h. The proliferation of HMEC-1 cells was evaluated by EdU assay and analyzed using the Image Pro Plus 6 software.

Calcium fluorescence detection: The cells were plated in 96-well plates (8,000 cells/well). The next day, the cells were washed with PBS and 5 μM Fluo-4-AM (Cat. S1060, Beyotime) was added to each well and incubated for 1 h. Then, the cells were washed three times with PBS. Menthol (Cat. 63660, Sigma-Aldrich) and icilin (Cat.I9532, Sigma, Sigma-Aldrich) were used to activate the TRPM8 channel and calcium influx. Fluorescence was recorded every 10 s for a time lapse of 10 min using

a microplate reader (TECAN, Switzerland) and analyzed using GraphPad Prism (San Diego, CA, USA).

Electrophysiological assay: Electrophysiological experiments were carried out on TPCs with TCAF2-overexpression and -knockdown, and patch-clamp recordings were performed using a HEKA EPC10 amplifier equipped with PatchMaster software (HEKA, MA). Whole cell recordings were performed at 80 mV. Patch pipettes made of borosilicate glass were polished by fire to a resistance of 3-6 MΩ, which was compensated for by 60% of the serial resistance. The standard bath and pipette solutions included 130 mM NaCl, 0.2 mM EDTA, and 3 mM HEPES, pH 7.2 for TRPM8 channel recording. Menthol (500 μM) was infused into the membrane patch using a gravity drive system (RSC-200; BioLogic). Different solutions were transported through separate pipes to avoid mixing. During recording, the patch straw was placed in front of the perfusion tube outlet. All recordings were performed at room temperature, with a maximum change of 1 °C.

Enzyme-linked immunosorbent assay (ELISA): The levels of Wnt5a in the supernatant of cells were measured using a human Wnt5a ELISA kit (Cat. EK2610, SAB, MD) according to the manufacturer's instructions.

In vivo cell tracking: Each mouse was injected intraperitoneally (i.p.) with 3 mg D-luciferin (Cat. 40901ES01, Yeason Biotechnology, Shanghai, China) dissolved in 200

μL saline. 5 min later, the mice were anesthetized with isoflurane. Luminescence signals were collected with Xenogen IVIS 200 (Alameda, CA, USA) every week and analyzed using Xenogen Living Image software (Alameda, CA).

Cell transfection and infection: For TCAF2 overexpression, TPCs derived from non-metastatic CRC (TPC_{NM}) were infected with lentivirus-carrying vector (Ubi-MCS-3FLAG-SV40-EGFP-IRES-puromycin) or TCAF2-overexpressing plasmid (Genechem) to generate TPC_{NM}^{Vector} and TPC_{NM}^{TCAF2}, respectively. For TCAF2 knockdown, TPCs derived from liver metastatic CRC (TPC_{LM}) were infected with lentivirus-carrying shNC (hU6-MCS-CBh-gcGFP-IRES-puromycin) or shTCAF2 (Genechem) to generate TPC_{LM}^{shNC} or $TPC_{LM}^{shTCAF2}$, respectively. For Wnt5a knockdown, TCAF2 overexpressing TPCs ($TPC_{NM-TCAF2}$) were infected with lentivirus-carrying shNC (hU6-MCS-CBh-gcGFP-IRES-puromycin) or shWNT5A (Genechem) to generate $TPC_{NM-TCAF2}^{shNC}$ or $TPC_{NM-TCAF2}^{shWNT5A}$, respectively. The cells were selected using puromycin (2 μg mL^{-1}) for 2 days, and the surviving cells were harvested for further experiments. For TRPM8 overexpression, TPC_{LM} were transfected with a vector or a TRPM8-overexpressing plasmid for 48 h to generate TPC_{LM}^{Vector} or TPC_{LM}^{TRPM8}, respectively. The details of the vector or TRPM8 plasmid are as follows: TRPM8 (NM_024080) human-tagged ORF clone (Cat. RC220615, Origene), cloning vector pCMV6-Entry mammalian expression vector (Cat. PS100001, Origene). For TRPM8-knockdown, TPC_{NM} were transfected with TRPM8 siRNA for 48 h, and the cells were harvested for subsequent analysis. For the TCF21-overexpressing assay, TPC_{NM} were infected with lentivirus-harboring the vector (Cat. PS100001, Origene) or the *TCF21* overexpression plasmid (Cat. RC220002, Origene) for 48 h, and then selected with puromycin (2 μg mL^{-1}). For the TCF21-knockdown

assay, TPC_{LM} were infected with lentivirus-carrying shNC or shTCF21 (Genechem) to generate TPC_{LM}^{shNC} and $TPC_{LM}^{shTCF21}$. To overexpress HIF-1α, TPC_{NM} were transfected with pCMV6-*HIF1A (*Cat. RC202461, Origene*)* and the vector for 48 h, followed by subsequent analysis. For the HIF-1α knockdown assay, TPC_{NM} was transfected with siRNA targeting *HIF1A* or a negative control (NC) for 48 h. Transfection was performed using Lipofectamine™ 3000 (Cat. L3000015, Invitrogen, Carlsbad, CA, USA), according to the manufacturer's instructions. The siRNA sequences used are listed in **Table S13**.

Isolation and identification of circulating tumor cells (CTCs): CTCs were isolated and identified as previously described. [4, 5] Briefly, blood was collected from mice and immediately released into the tubes coated with heparin to prevent coagulation. The obtained blood was treated with red blood cell lysis buffer (Cat. C3702; Beyotime), and the remaining cells were cultured in DMEM supplemented with 20% FBS. The adherent tumor cells were identified and counted within 12 h. Adherent cells were co-stained with EpCAM (cancer cell-associated surface marker) and CD45 (leukocyte marker) and identified by confocal microscopy. Cells positive for EpCAM but negative for CD45 were considered as CTCs and were subsequently subjected to manual counting. The CTC counts are presented as CTCs per milliliter of whole blood.

ChIP-qPCR assay: The ChIP-qPCR assay was performed using the SimpleChIP™ Enzymatic Chromatin IP Kit (Cat. 9003, Cell Signaling Technology, MA, USA) according to the manufacturer's instructions. The primers of *HIF1A* promoter were: Forward primer (5'-3'): TAGGCAATGAGGACTCAATGGT; Reverse primer (5'-3'): TACGCAAGA-GAGGTCAGAAGGTG. The signal relative to the input was

calculated as follows: percent input = 2% $\times$ $2^{(C[T]2\%\text{input sample} - C[T]\text{IP sample})}$, where C[T] is the threshold cycle of the PCR reaction.

RNA-seq analysis: Total RNA was isolated from cells using TRIzol reagent (Cat. 15596018, Invitrogen, Carlsbad, CA, USA), according to the manufacturer's instructions. RNA concentration and integrity were determined using a NanoDrop ND-1000 (NanoDrop, Wilmington, DE) and Bioanalyzer 2100 (Agilent, CA), respectively. Poly (A) RNA was purified from 1μg of total RNA using Dynabeads Oligo (dT) 25 (Cat. 61005, Thermo Scientific) using two rounds of purification, which were then cut into pieces using the Magnesium RNA Fragmentation Module (Cat. e6150, NEB, NY) under 94 °C for 5-7 min. The fragmented RNA pieces were reversely transcribed into cDNA using SuperScript™ II Reverse Transcriptase (Cat.1896649, Invitrogen) and sequenced with an Illumina Novaseq 6000 (LC-Bio Technology CO., Ltd., Hangzhou, China). StringTie and edgeR were used to evaluate the levels of all the transcripts. The differentially expressed mRNAs and genes were selected with log2 (fold change) >1.5 and *P*-value < 0.05 using the R package edgeR. The heatmap revealed the differentially expressed genes.

Supplementary figures and figure legends

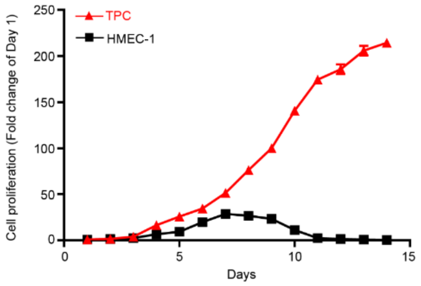

Figure S1. Growing curves of TPCs and HMEC-1 cells cultured in pericyte medium (PM). TPCs (1× 10^3) and HMEC-1 cells (1× 10^3) were respectively cultured by PM in 96-well plates (Day 0). Cell viability was examined by MTT assay every day for 14 days (Day 1-Day 14).

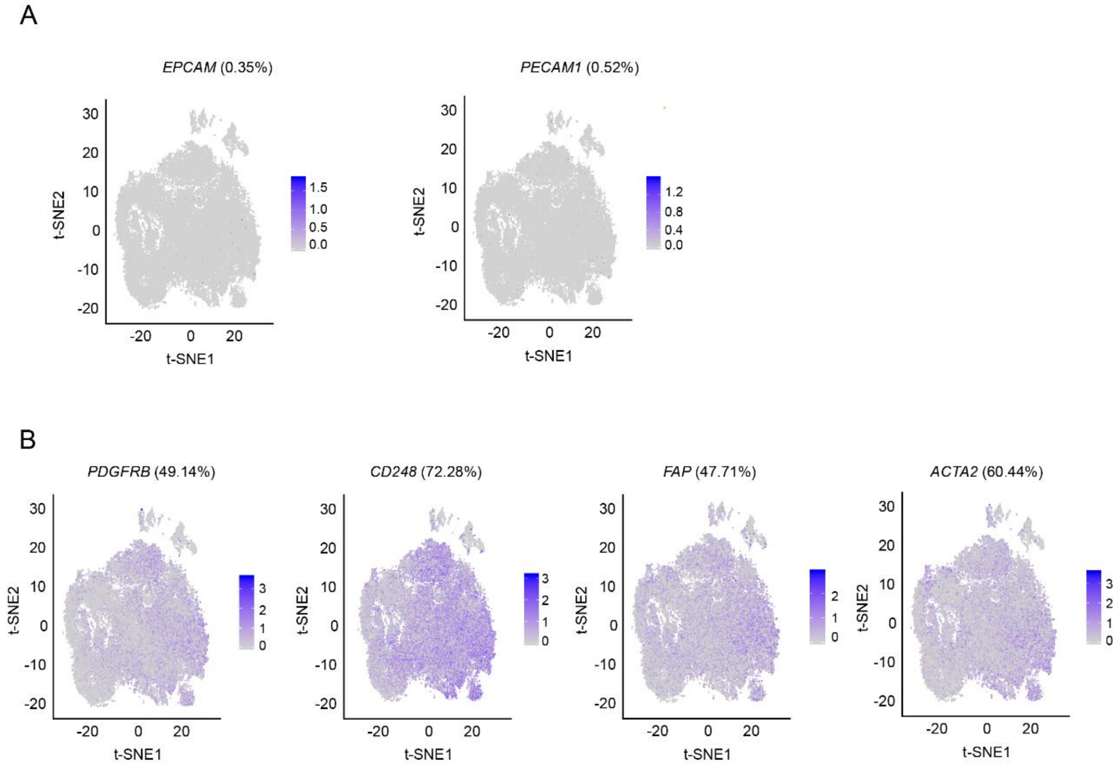

Figure S2. Transcriptomic characterization of TPCs. (A) Gene expression profiles of *PECAM1* and *EPCAM* in distinct subsets of TPCs. The percentage of *PECAM1*$^+$ and

EPCAM+ TPCs were shown. **(B)** Gene expression profiles of *PDGFRB*, *ACTA2*, *CD248* and *FAP* in distinct subsets of TPCs. The ratios of indicated TPCs were shown.

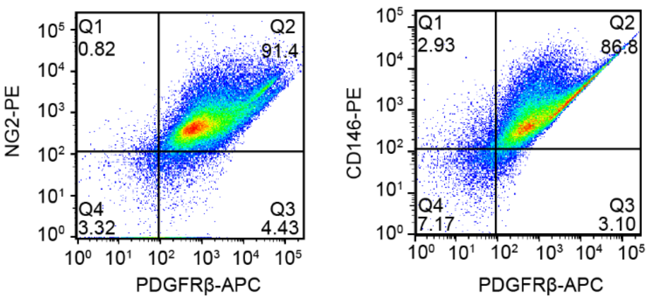

Figure S3. Identification of the MPMA-obtained TPCs. Flow cytometry analysis of NG2/ PDGFRβ and CD146/ PDGFRβ expression in TPCs (n = 3).

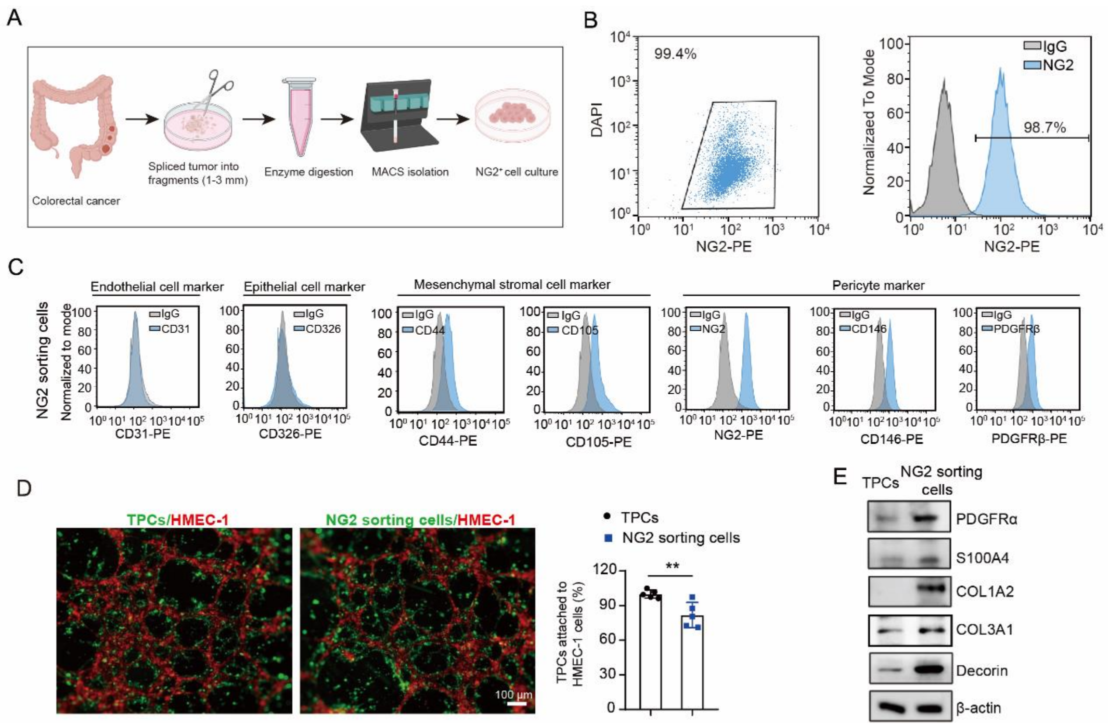

Figure S4. Isolation and characterization of the MACS-obtained NG2+ cells. **(A)** Diagram depicting the isolation of NG2+ cells from patients with colorectal cancer.

Primary colorectal cancer tissue was acquired and spliced into small fragments, followed by digestion with Type I and II collagenase. The resulting single cell suspensions were isolated using anti-NG2 magnetic beads. (**B**) Flow cytometry analysis for NG2 expression in sorted cells (n = 3). **(C)** Flow cytometry analysis for the expression of markers specific to endothelial cells, epithelial cells, mesenchymal stromal cells and pericytes in NG2-sortng cells (n = 3). **(D)** Representative images and quantification for TPCs or $NG2^+$ cells (green) attached to the tubes formed by HMEC-1 cells (red) (n = 5). Scale bar, 100 μm. (**E**) Western blot analysis for the expression of PDGFRα, S100A4, COL1A2, COL3A1, and decorin in MPMA-obtained TPCs and NG2-sortng cells. Data are presented as mean ± SEM. $^{**}P < 0.01$ by two-tailed unpaired *t*-test. HMEC-1, human microvascular endothelial cell-1; MACS, magnetic-activated cell sorting; TPC, tumor pericyte.

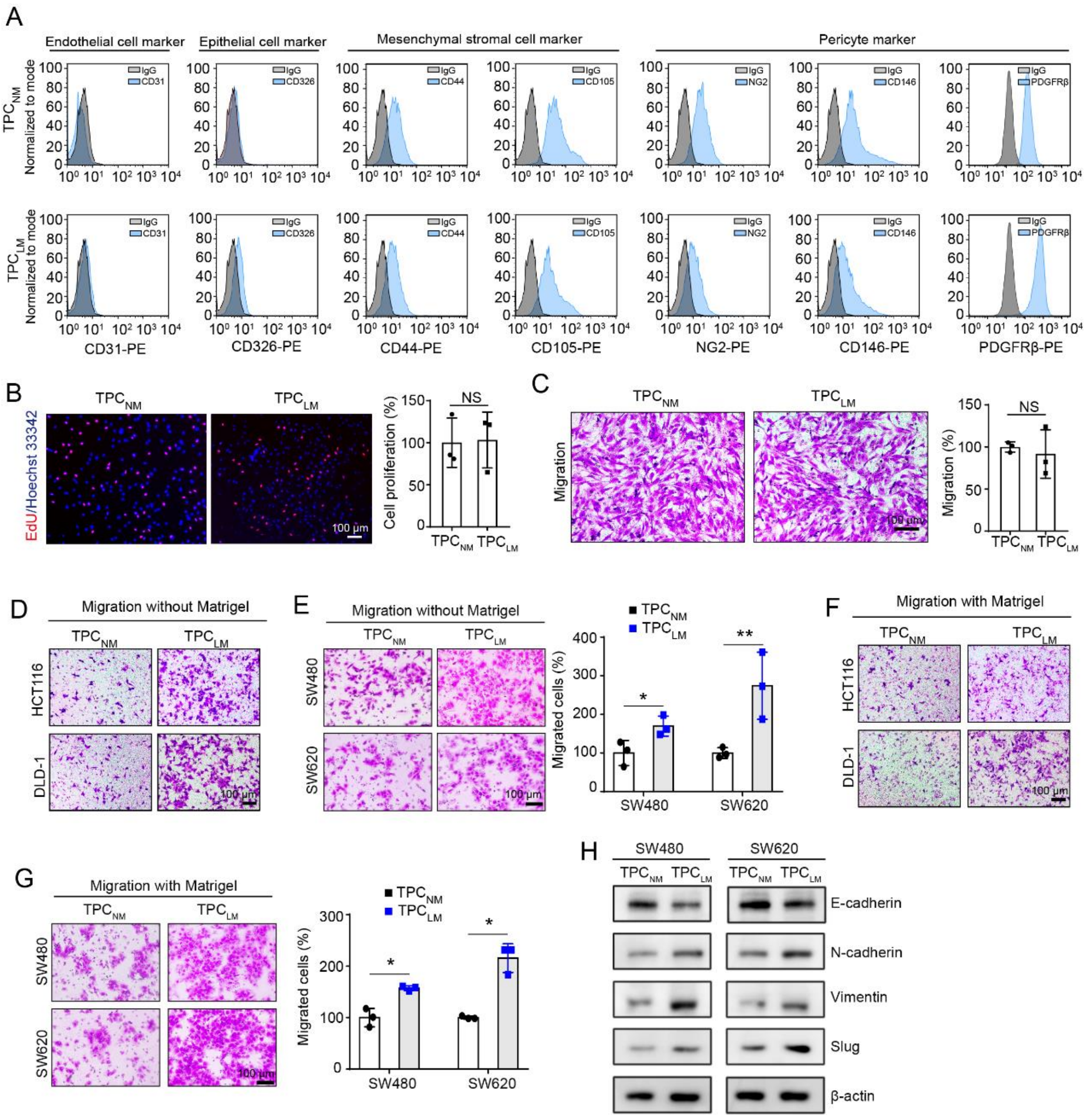

Figure S5. TPCs derived from liver-metastatic CRC patients facilitate tumor cell migration. (**A**) Flow cytometry analysis for the expression of markers specific to endothelial cells, epithelial cells, mesenchymal stromal cells and pericytes in TPC_{NM} and TPC_{LM} (n = 3). (**B**) EdU staining and quantification for cell proliferation of TPC_{NM} and TPC_{LM} (n = 3). Scale bar, 100 μm. (**C**) Transwell assay for the migration of TPC_{NM} and TPC_{LM} (n = 3). Scale bar, 100 μm. (**D-G**) Transwell assay for the migration without (**D, E**) and with Matrigel (**F, G**) of colorectal cancer cells primed with the conditioned medium from TPC_{NM} and TPC_{LM}, respectively (n = 3). Scale bar, 100 μm. (**H**) Western blot analysis of E-cadherin, N-cadherin, Vimentin, and Slug in SW480 and SW620

cells primed with conditioned medium from TPC_{NM} and TPC_{LM}, respectively. Data are presented as mean ± SEM. $^{*}P < 0.05$, $^{**}P < 0.01$, NS by two-tailed unpaired *t*-test. LM, liver metastasis; NS, not significant; NM, non-metastasis; TPC, tumor pericyte.

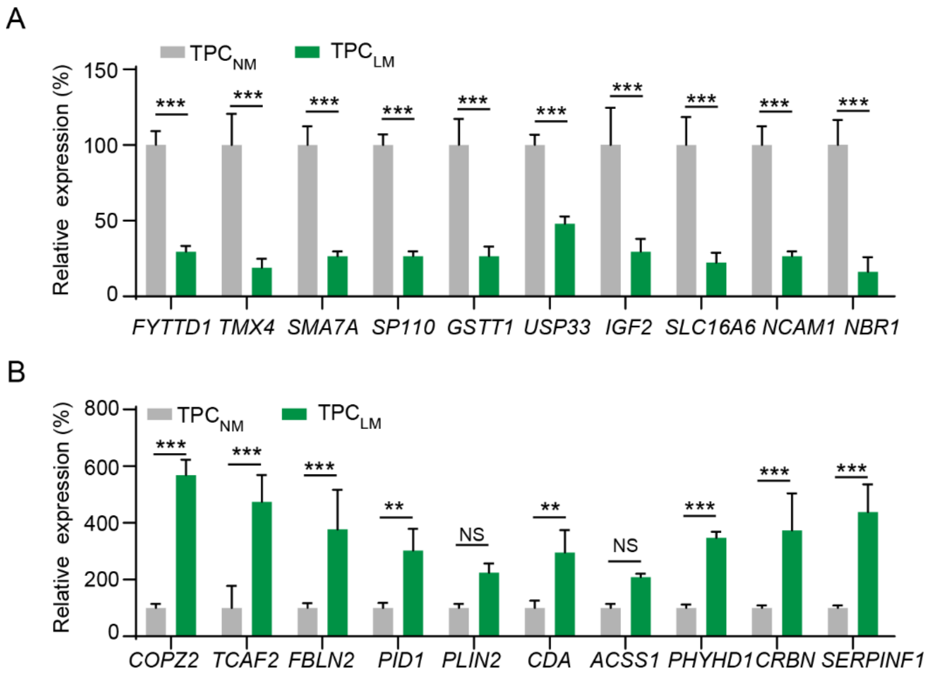

Figure S6. TCAF2 is highly expressed in TPC_{LM}. (A) RT-qPCR analysis of top ten down-regulated genes in TPC_{LM} versus TPC_{NM} derived from the proteomic data (n = 3). **(B)** RT-qPCR analysis of top ten up-regulated genes in TPC_{LM} versus TPC_{NM} derived from proteomic data (n = 3). Data are presented as mean ± SEM. NS, $^{**}P < 0.01$, $^{***}P < 0.001$ by two-tailed unpaired *t*-test. LM, liver metastasis; NM, non-metastasis; NS, not significant; TPC, tumor pericyte.

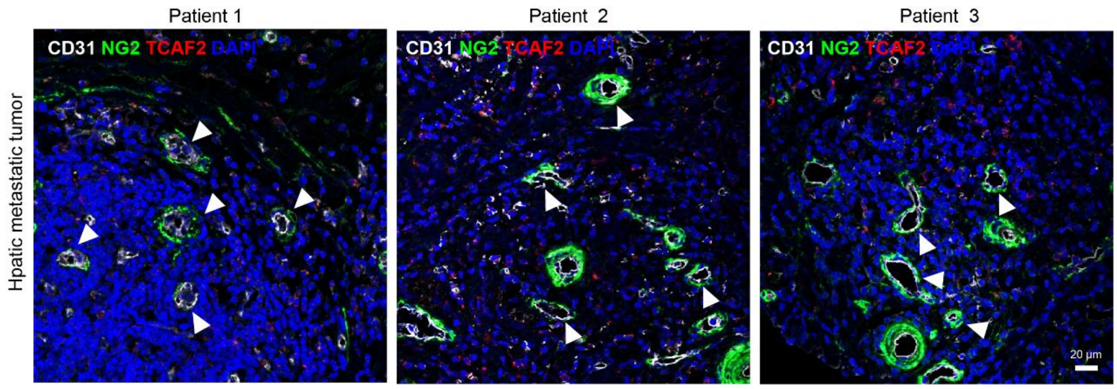

Figure S7. TCAF2 is undetected in TPCs in the hepatic metastatic tumors. Representative images of TCAF2 staining (red) in TPCs ($NG2^+$, green) in hepatic metastases derived from CRC patients (n = 20). White arrowheads indicate TCAF2 expression in TPCs. Scale bar, 20 μm. DAPI, 2-(4-Amidinophenyl)-6-indolecarbamidine dihydrochloride; TPC, tumor pericyte.

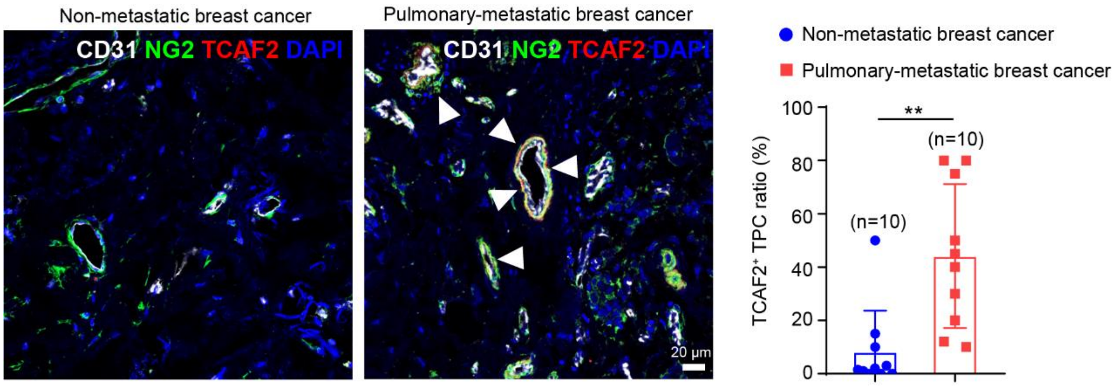

Figure S8. TCAF2 is highly-expressed in TPCs derived from pulmonary-metastatic breast cancer. Immunofluorescence analysis for the expression of TCAF2 in primary tumor sections derived from patients with breast cancer. White arrowheads indicate the $TCAF2^+$ TPCs. Scale bar, 20 μm. Data are presented as mean ± SEM (n = 10). $^{**}P < 0.01$ by two-tailed unpaired *t*-test. DAPI, 2-(4-Amidinophenyl)-6-indolecarbamidine dihydrochloride; TPC, tumor pericyte.

A

Cell line	*KRAS*	*BRAF*
DLD-1	mutant	wt
HCT116	mutant	wt
LoVo	mutant	wt
SW480	mutant	wt
SW620	mutant	wt
HT-29	wt	mutant
WiDr	wt	mutant

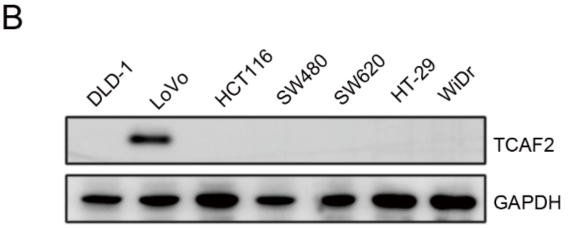

Figure S9. TCAF2 in TPCs has no correlation with KRAS or BRAF mutation in CRC cells. (A) Diagram of *KRAS* and *BRAF* mutations in CRC cell lines. **(B)** Western blot analysis of TCAF2 expression in TPC_{NM} primed with conditional medium of indicated CRC cells for 24 h.

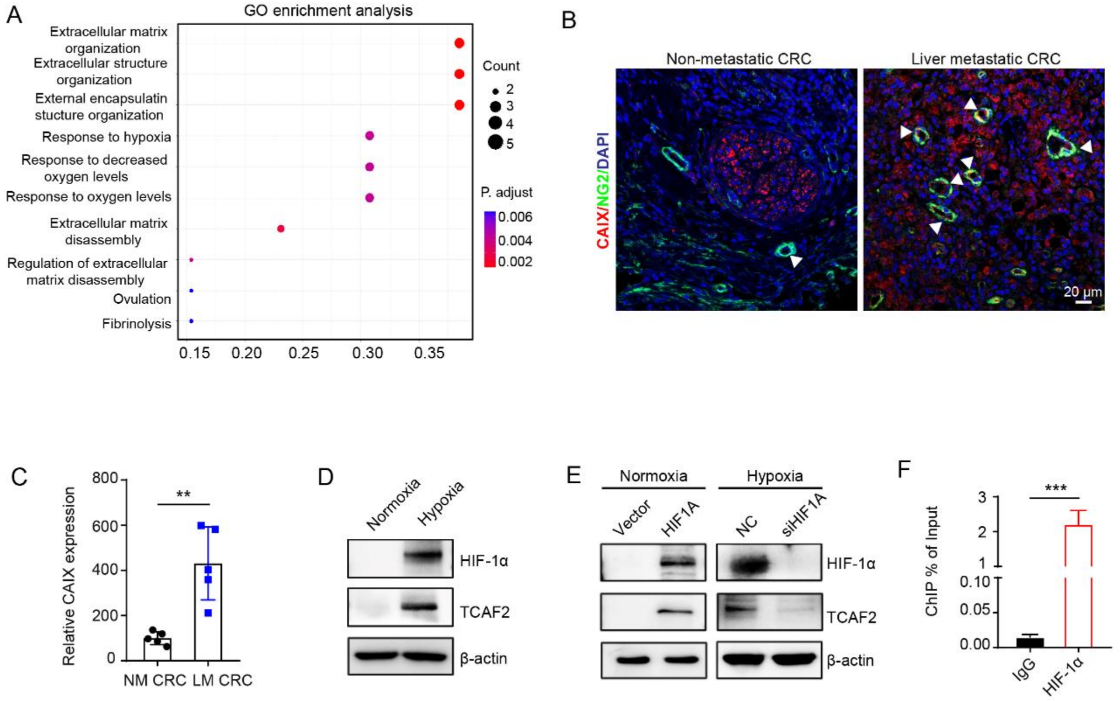

Figure S10. Hypoxia induces TCAF2 expression in TPCs. (A) GO analysis of the upregulated genes in TPC_{LM} compared to TPC_{NM}. **(B, C)** Representative images **(B)** and quantification **(C)** for CAIX expression in primary tumor tissues derived from patients with CRC (n = 5). White arrowheads indicate the perivascular region. Scale

bar, 20 μm. **(D)** Western blot analysis of HIF-1α and TCAF2 in normoxic or hypoxic TPCs. **(E)** Western blot analysis of HIF-1α and TCAF2 in normoxic or hypoxic TPCs with HIF-1α-overexpression or -knockdown. **(F)** ChIP-qPCR analysis of HIF-1α binding to the *TCAF2* promoter. Data are presented as mean ± SEM. $^{***}P < 0.001$ by two-tailed unpaired *t*-test. CAIX, Carbonic Anhydrase 9; GO, gene ontology; HIF-1α, hypoxia-inducible factor-1 alpha. DAPI, 2-(4-Amidinophenyl)-6-indolecarbamidine dihydrochloride; CRC, colorectal cancer.

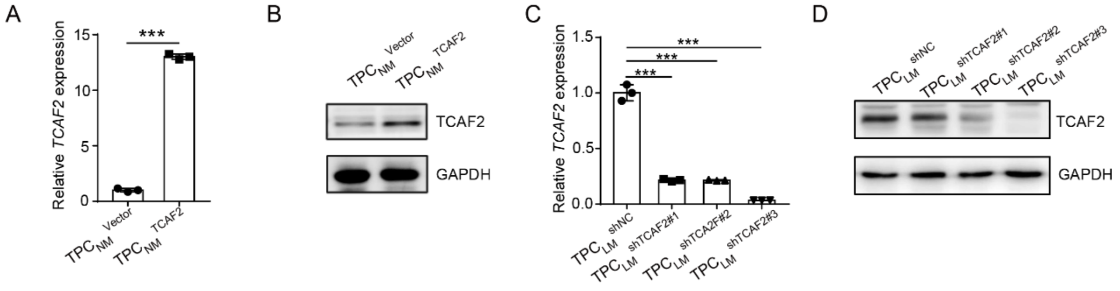

Figure S11. Characterization of TCAF2-overexpressing and -knockdown TPCs. (A, B) Non-metastatic TPCs with intrinsic low expression of TCAF2 were infected with lentivirus containing a TCAF2-overexpression plasmid (TPC_{NM}^{TCAF2}) or Vector (TPC_{NM}^{Vector}). The expression levels of TCAF2 were determined by RT-qPCR **(A)** and Western blot **(B)**, respectively (n = 3). **(C, D)** Liver-metastatic TPCs with intrinsic overexpression of TCAF2 were transfected with lentivirus containing shTCAF2 ($TPC_{LM}^{shTCAF2}$) or shNC (TPC_{LM}^{shNC}). The expression levels of TCAF2 were determined by RT-qPCR (**C**) and Western blot (**D**), respectively (n = 3). Data are presented as mean ± SEM. $^{***}P < 0.001$ by two-tailed unpaired *t*-test in **A**; by one-way

ANOVA followed by Tukey's post hoc test in **C**. LM, liver metastasis; NM, non-metastasis; TPC, tumor pericyte.

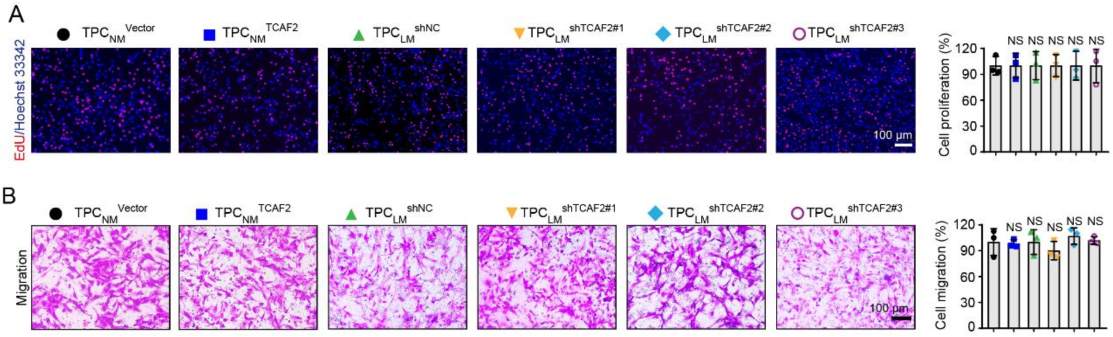

Figure S12. Effects of TCAF2 on the proliferation and migration of TPCs. (A) Representative images and quantification of proliferation in TCAF2- overexpressing or -knockdown TPCs (n = 3). Scale bar, 100 μm. **(B)** Transwell assay and quantification for the migration of TCAF2-overexpressing or -knockdown TPCs (n = 3). Scale bar, 100 μm. Data are presented as mean ± SEM. NS compared with TPC_{NM}^{Vector} by one-way ANOVA followed by Tukey's post hoc test. LM, liver metastasis; NM, non-metastasis; NS, not significant; TPC, tumor pericyte.

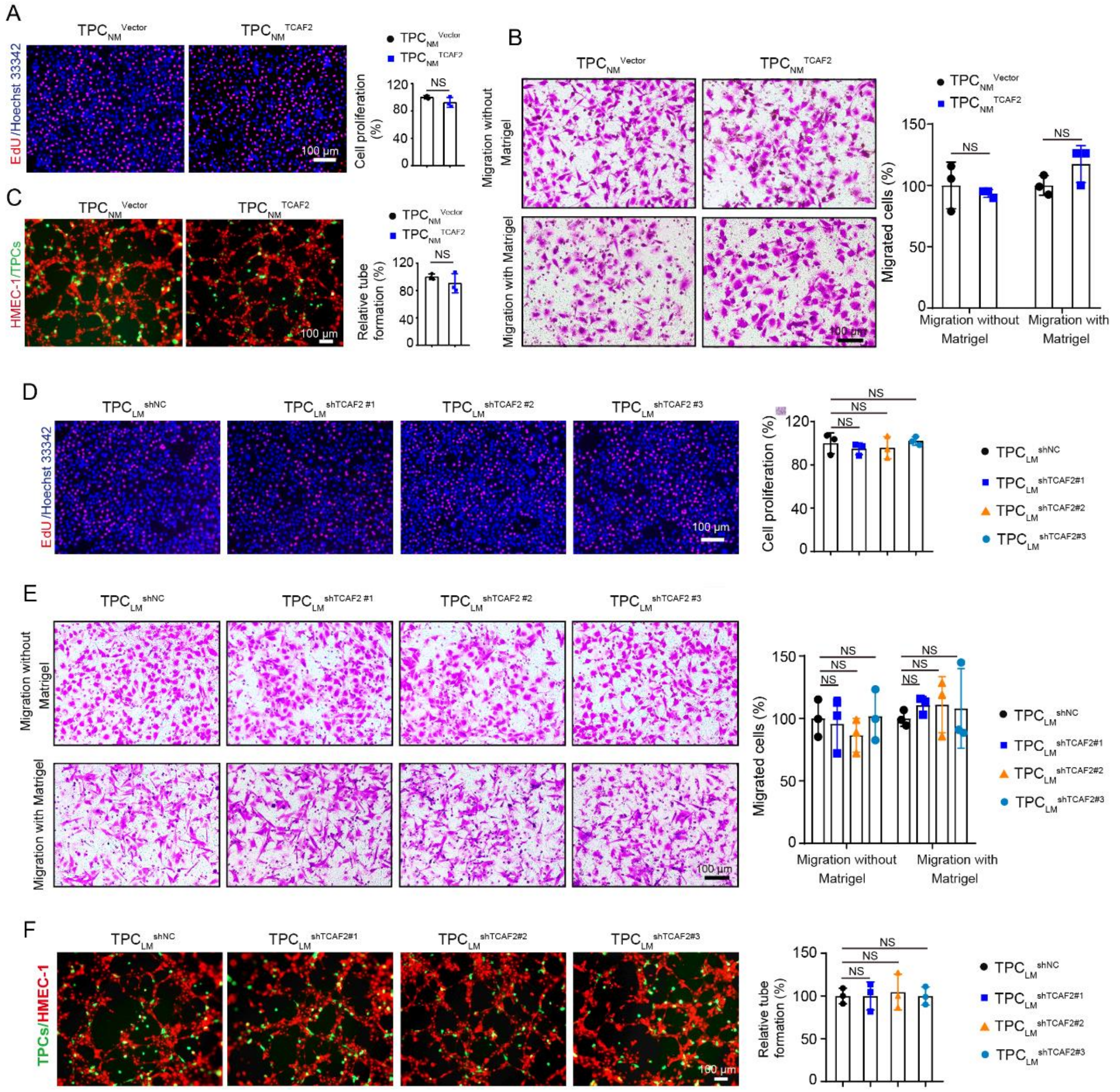

Figure S13. TCAF2 in TPCs has negligible effects on endothelial proliferation, migration and tube formation. (A) EdU assay for the proliferation of HMEC-1 cells co-cultured with TPC_{NM}^{Vector} and TPC_{NM}^{TCAF2} for 24 h (n = 3). Scale bar, 100 μm. **(B)** Transwell assay for the migration of HMEC-1 cells co-cultured with TPC_{NM}^{Vector} and TPC_{NM}^{TCAF2} (n = 3). HMEC-1 cells were seeded on the upper chamber of the transwell, while TPC_{NM}^{Vector} and TPC_{NM}^{TCAF2} were plated in the bottom compartments and co-cultured for 48 h. The migrated cells were imaged and counted. Scale bar, 100 μm. **(C)** Representative images and quantification of endothelial tubes formed by HMEC-1 cells.

PHK26-labeled HMEC-1 cells (red) were co-cultured with PKH67-labeled TPC_{NM}^{Vector} or TPC_{NM}^{TCAF2} (green) for 6 h (n = 3). Scale bar, 100 μm. **(D)** EdU assay for the proliferation of HMEC-1 cells co-cultured with TCAF2-knockdown TPCs for 24 h (n = 3). Scale bar, 100 μm. **(E)** Transwell assay for the migration of HMEC-1 cells co-cultured with TPC_{LM}^{shNC} and $TPC_{LM}^{shTCAF2}$ for 48 h (n = 3). **(F)** Tube formation assay for HMEC-1 cells co-cultured with TPC_{LM}^{shNC} and $TPC_{LM}^{shTCAF2}$ as indicated in **(C)** (n = 3). Scale bar, 100 μm. Data are presented as mean ± SEM, Two-tailed unpaired t-test **(A, B, C)**, one-way ANOVA followed by Tukey's post hoc test **(D, E, F).** NS, not significant; TPC, tumor pericyte; NM, non-metastasis; LM, liver metastasis; HMEC-1, human microvascular endothelial cell-1.

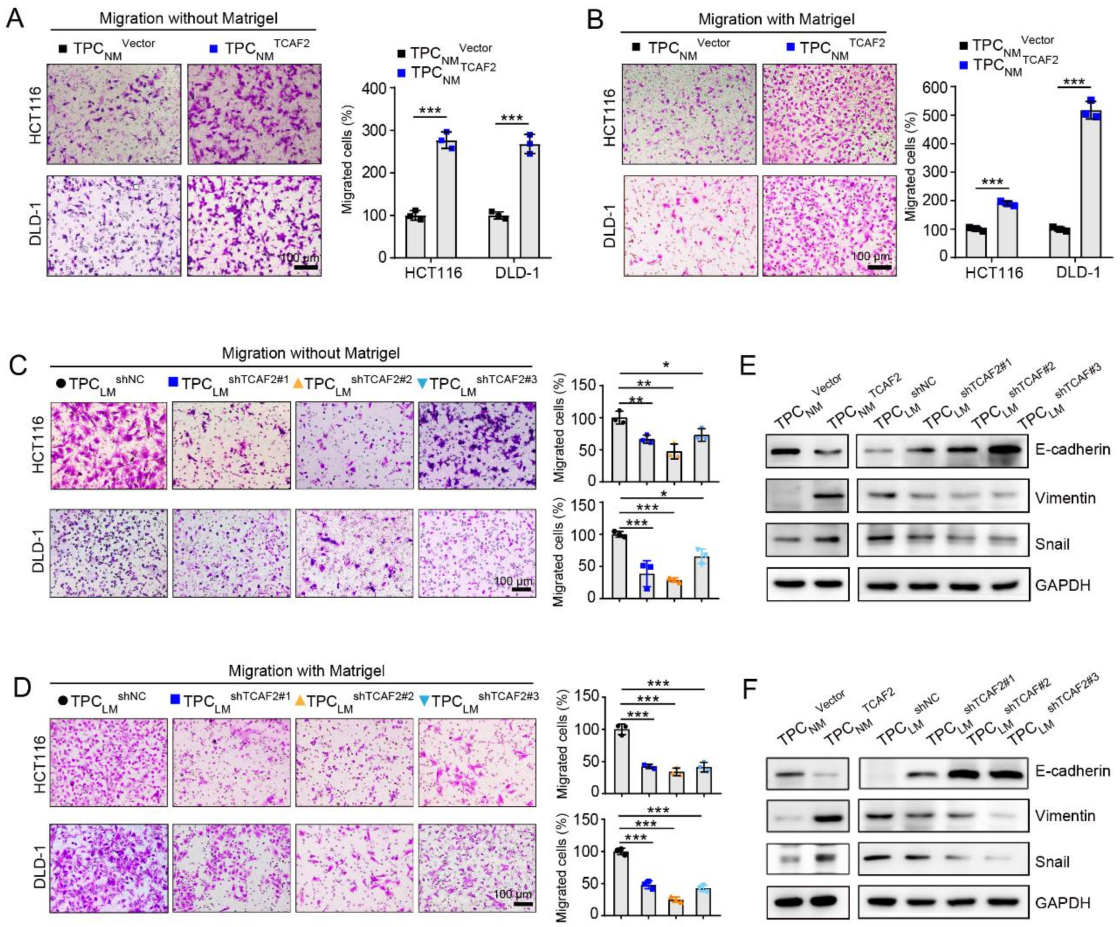

Figure S14. Effects of TCAF2 in TPCs on the motility and EMT in HCT116 and DLD-1 cells. (A, B) Transwell assays for the migration without **(A)** or with Matrigel (**B**) of CRC cells primed with the conditioned medium from TCAF2-overexpressing TPCs (n = 3). Scale bar, 100 μm. **(C, D)** Transwell assays for the migration without **(C)** or with Matrigel (**D**) of CRC cells primed with the conditioned medium from TCAF2-knockdown TPCs (n = 3). Scale bar, 100 μm. **(E, F)** Western blot analysis of EMT markers in HCT116 (**E**) and DLD-1 (**F**) cells primed with the conditioned medium from the indicated TPCs. Data are presented as mean ± SEM. $^{*}P < 0.05$, $^{**}P < 0.01$, $^{***}P < 0.001$ by two-tailed unpaired *t*-test in **A**, **B**; by one-way ANOVA followed by Tukey's post hoc test in **C**, **D**. LM, liver metastasis; NM, non-metastasis; TPC, tumor pericyte.

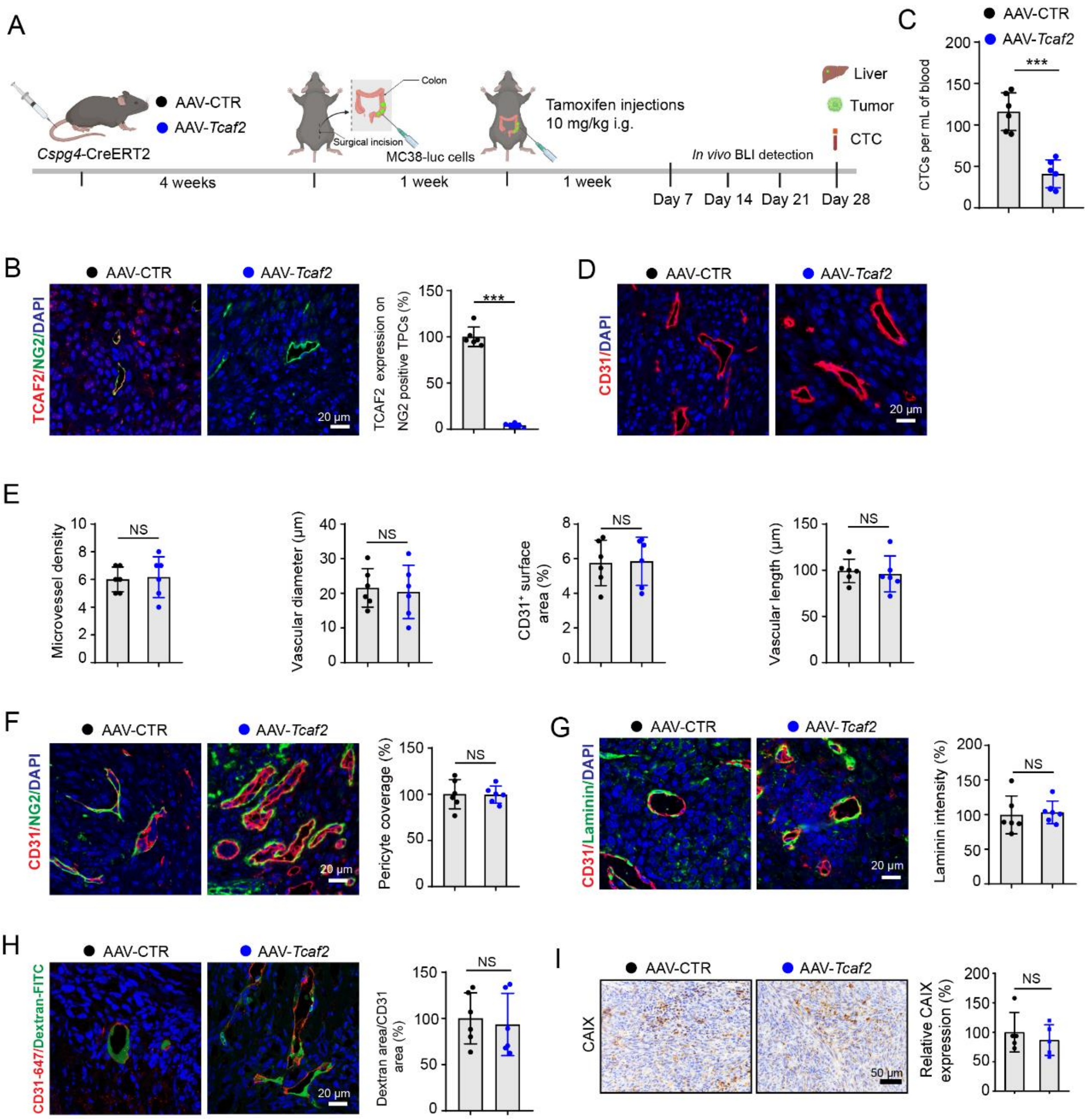

Figure S15. Pericyte specific deletion of *Tcaf2* has negligible effects on the morphology and function of tumor vessels. (**A**) Schematic diagram of animal experiments. **(B)** Immunofluorescence analysis for TCAF2 expression on NG2^{+} TPCs in primary tumor tissues derived from MC38-luc orthotopic allografts (n = 6). Scale bar, 20 μm. **(C)** Quantification of CTCs derived from mice bearing MC38-luc orthotopic allografts (n = 6). **(D, E)** Immunofluorescence and quantification of CD31^{+} surface area, microvessel density, vascular diameter and vascular length in primary tumor tissues derived from MC38-luc orthotopic allografts (n = 6). Scale bar, 20 μm. **(F)** Immunofluorescence staining for NG2^{+} (green) pericyte coverage in CD31^{+} (red)

blood vessels in in primary tumor tissues derived from MC38-luc allografts (n = 6). Scale bar, 20 μm. **(G)** Immunofluorescence staining and quantification of laminin (green) surrounding CD31$^+$ (red) blood vessels in primary tumor tissues derived from MC38-luc orthotopic allografts (n = 6). Scale bar, 20 μm. **(H)** Representative images and quantification of CD31$^+$ blood vessels (red) and overlaid with the i.v. injected FITC-dextran in primary tumor tissues derived from MC38-luc orthotopic allografts (n = 6). Scale bar, 20 μm. **(I)** Immunohistochemical staining and quantification of CAIX in primary tumor tissues derived from MC38-luc orthotopic allografts (n = 6). Scale bar, 50 μm. Data are presented as mean ± SEM. NS, $^{***}P < 0.001$ by two-tailed unpaired *t*-test. AAV, adeno-associated virus; CTR, control; CTC, circulating tumor cell; CAIX, Carbonic Anhydrase 9; NS, not significant.

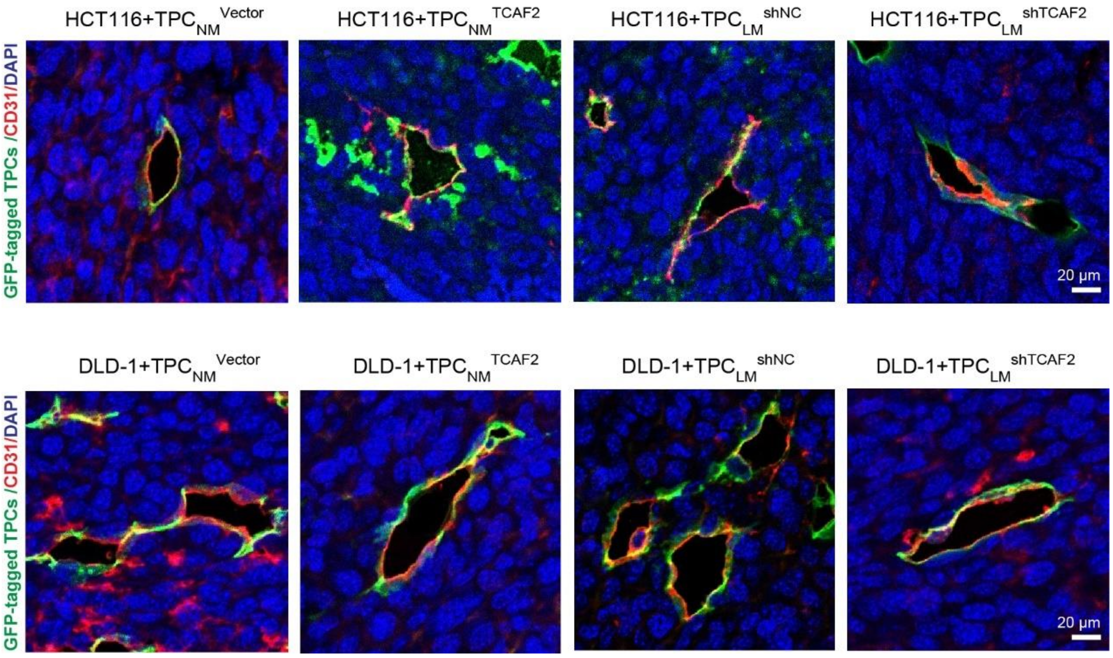

Figure S16. Determination of the GFP-tagged TPCs in primary tumor tissues. Immunofluorescence analysis of GFP-tagged TPCs in the primary tumor tissues derived from HCT116 or DLD-1 CRCLM xenografts at the end of experiments (n = 6).

Scale bar, 20 μm. GFP, green fluorescence protein; LM, liver metastasis; NM, non-metastasis; TPC, tumor pericyte.

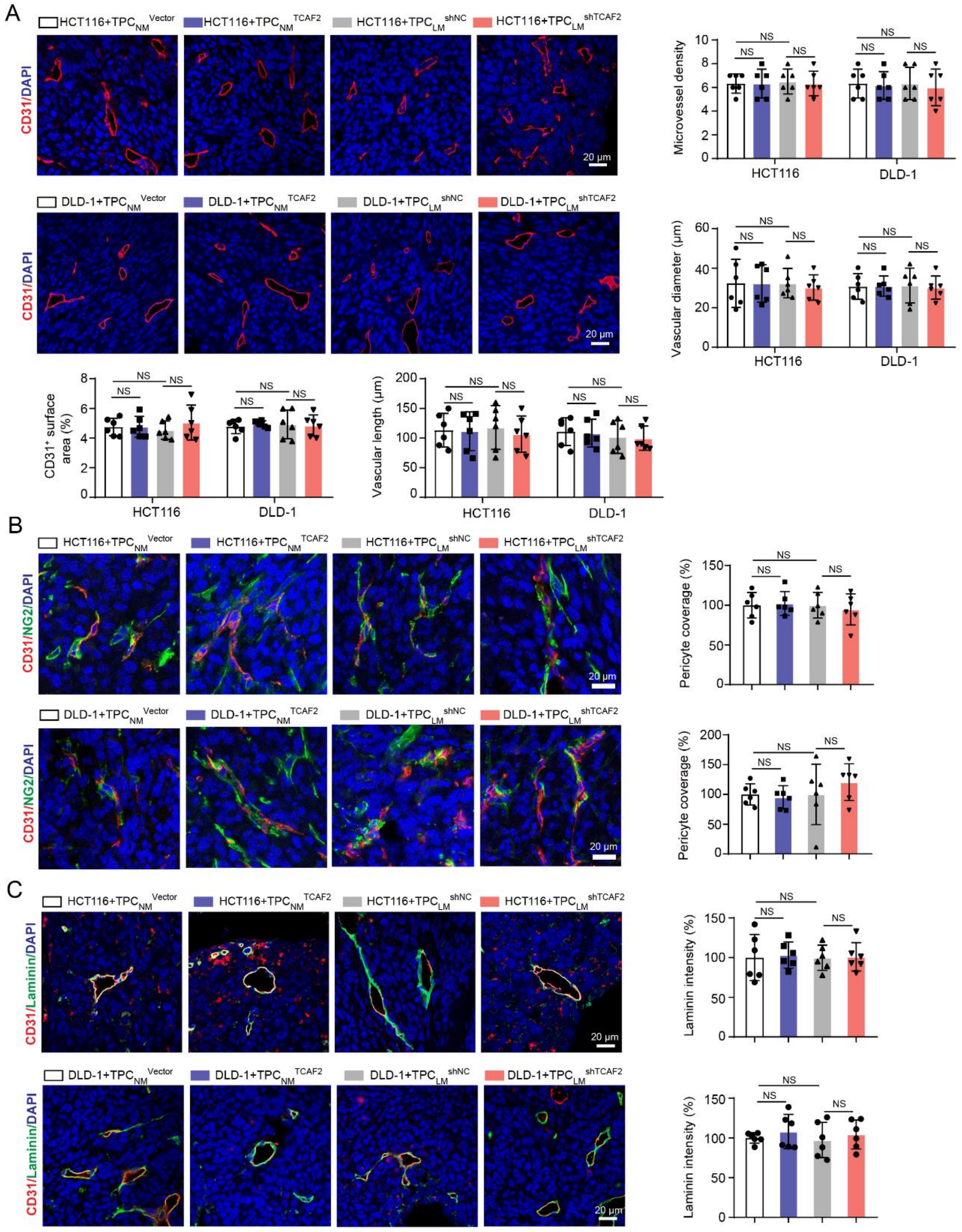

Figure S17. TCAF2 in TPCs has negligible effects on the morphology and

function of tumor vessels. (A) Immunofluorescence staining and quantification of CD31$^+$ surface area, microvessel density, vascular diameter and vascular length in primary tumor tissues derived from CRCLM xenografts (n = 6). Scale bar, 20 μm. Tumor cells pre-mixed with the indicated TPCs were co-injected into the cecum wall of mice to construct the CRCLM xenografts. **(B)** Immunofluorescence staining and quantification of NG2$^+$ (green) pericyte coverage of CD31$^+$ (red) blood vessels in primary tumor tissues derived from CRCLM xenografts (n = 6). Scale bar, 20 μm. **(C)** Immunofluorescence staining and quantification of laminin (green) surrounding CD31$^+$ (red) blood vessels in orthotopic tumor tissues derived from HCT116 and DLD-1 xenografts (n = 6). Scale bar, 20 μm. Data are presented as mean ± SEM. NS by one-way ANOVA followed by Tukey's post hoc test. LM, liver metastasis; NS, not significant; NM, non-metastasis; TPC, tumor pericyte.

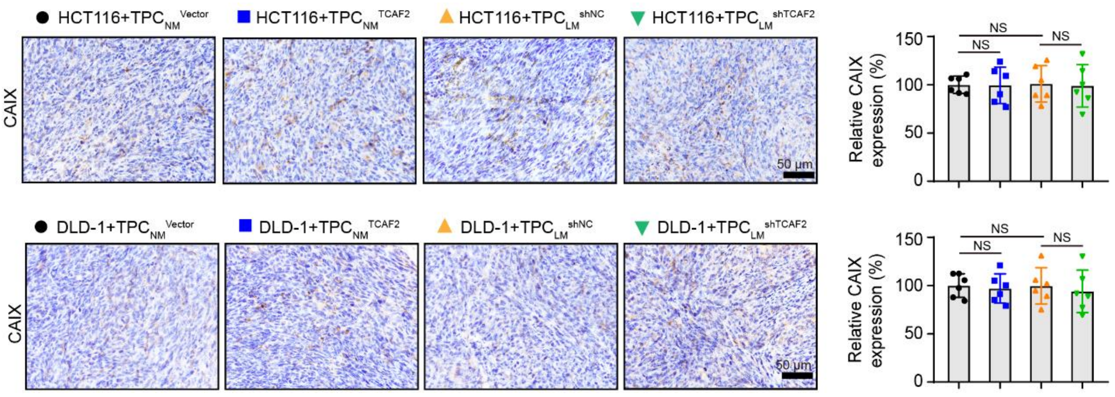

Figure S18. TCAF2 has negligible effects on tumor hypoxia. Immunohistochemical staining and quantification of CAIX in the primary tumor tissues derived from HCT116 and DLD-1 xenografts (n = 6). Scale bar, 50 μm. Data are presented as mean ± SEM. NS by one-way ANOVA followed by Tukey's post hoc test. CAIX, Carbonic

Anhydrase 9; LM, liver metastasis; NS, not significant; NM, non-metastasis; TPC, tumor pericyte.

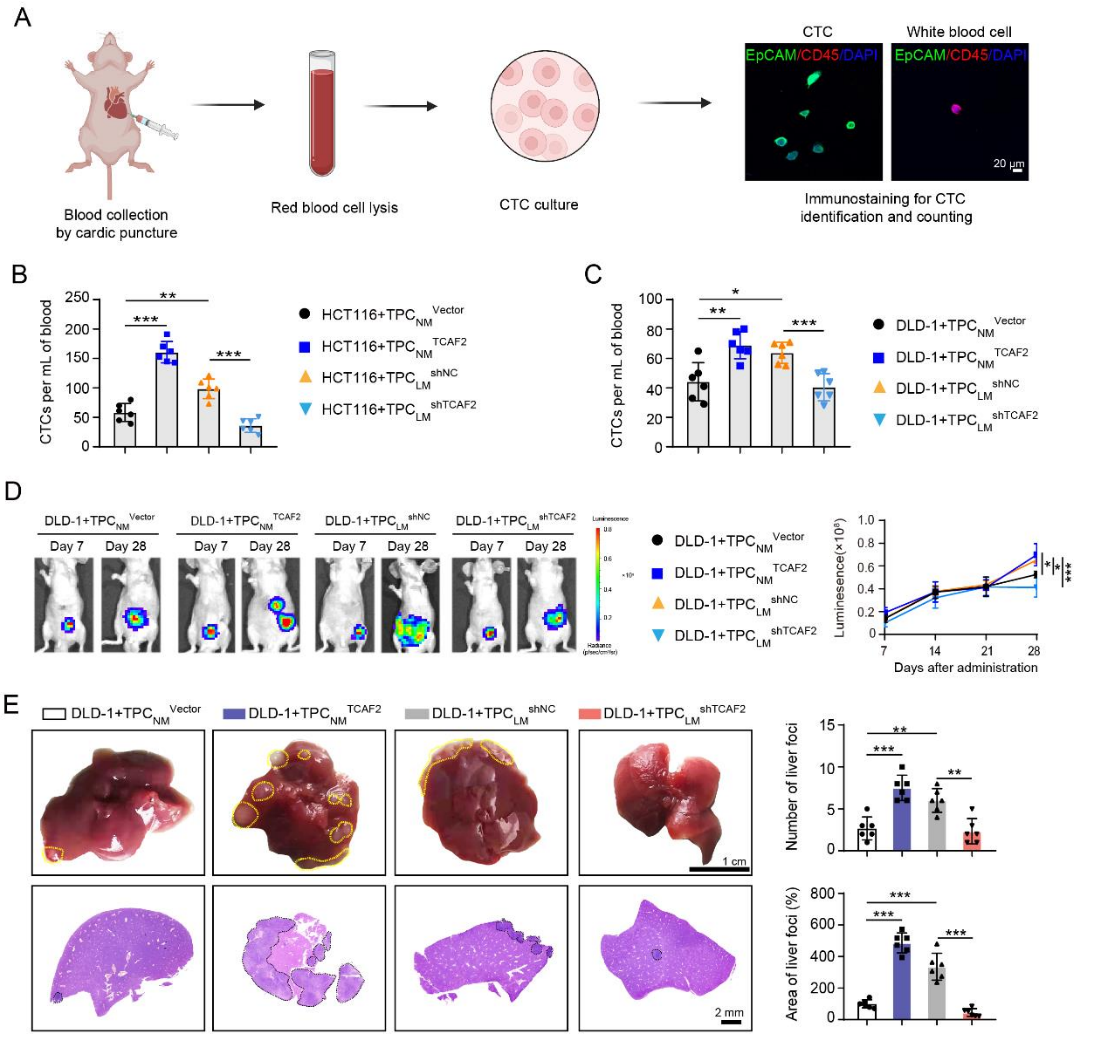

Figure S19. Effects of TCAF2 in TPCs on the liver metastasis of HCT116 and DLD-1 xenografts. **(A)** Schematic diagram of the isolation and identification of CTCs. Scale bar, 20 μm. **(B, C)** Quantification of CTCs derived from mice bearing HCT116 **(B)** and DLD-1 xenografts **(C)** (n = 6). **(D)** Bioluminescence analysis of mice co-injected with TPCs and DLD-1-luc cells (n = 6). **(E)** Representative images of liver metastases and the corresponding H&E staining. Mice were orthotopically transplanted with DLD-1 xenografts, and the primary tumor tissues and liver metastases were

harvested and photographed at the end of experiments. Scale bar, 1 cm (up); 2 mm (down). Quantification of the number and area of liver metastatic loci is shown (n = 6). Yellow and black dotted lines indicated liver metastatic foci. Data are presented as mean ± SEM. $^{*}P < 0.05$, $^{**}P < 0.01$, $^{***}P < 0.001$ by one-way ANOVA followed by Tukey's post hoc test. CTC, circulating tumor cell; LM, liver metastasis; NM, non-metastasis; TPC, tumor pericyte.

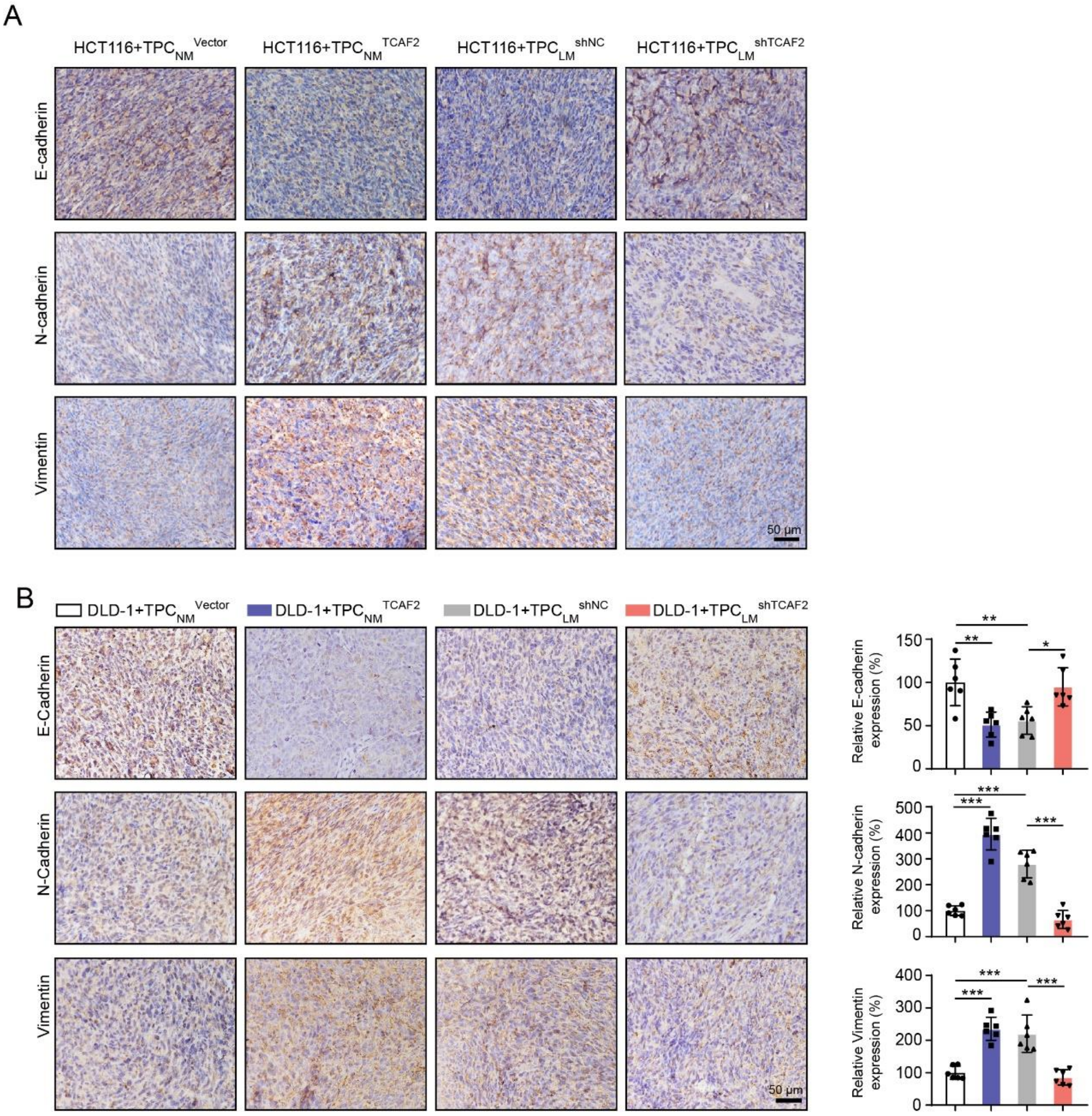

Figure S20. Effects of TCAF2 in TPCs on the EMT of HCT116 and DLD-1 xenografts. **(A)** Immunohistochemical staining of EMT markers in HCT116 orthotopic

tumor tissues (n = 6). Scale bar, 50 μm. **(B)** Immunohistochemical staining and quantification of E-cadherin, N-cadherin and Vimentin expression in DLD-1 orthotopic tumor tissues (n = 6). Scale bar, 50 μm. Data are presented as mean ± SEM. $^{*}P < 0.05$, $^{**}P < 0.01$, $^{***}P < 0.001$ by one-way ANOVA followed by Tukey's post hoc test. EMT, epithelial-mesenchymal transition; LM, liver metastasis; NM, non-metastasis; TPC, tumor pericyte.

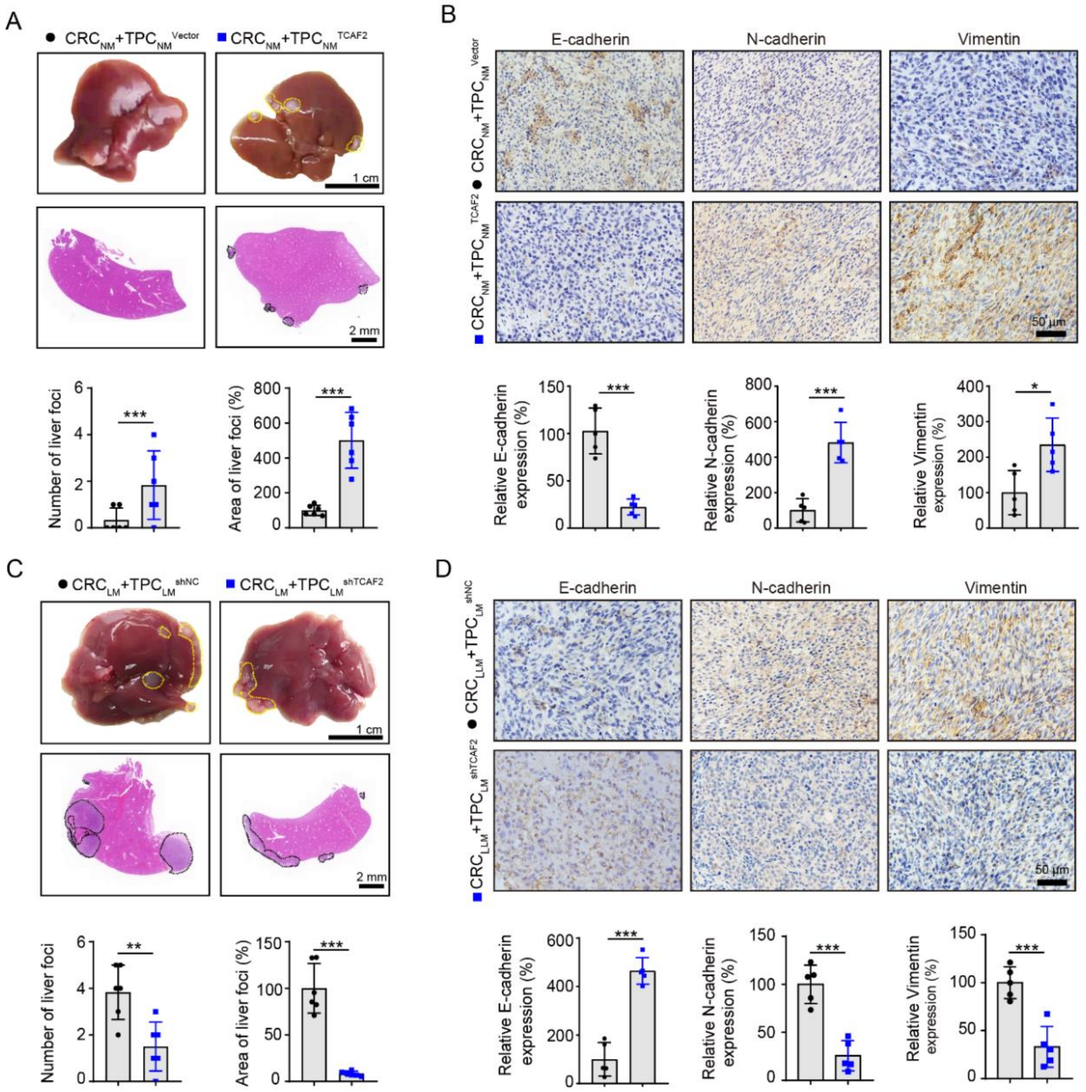

Figure S21. Effects of TCAF2 in TPCs on liver metastasis of CRC PDXs. (A) Representative images of liver metastases and the corresponding H&E staining. Mice

were orthotopically transplanted with non-metastatic CRC cells (CRC_{NM}) and TCAF2-overexpressing TPC_{NM}, and the primary tumor tissues and liver metastases were harvested and photographed at the endpoint of experiments. Scale bar, 1 cm (up); 2 mm (down). Quantification of the number and area of liver metastatic loci is shown (n = 6). Yellow and black dotted lines indicate liver metastatic foci. **(B)** Immunohistochemical staining and quantification of E-cadherin, N-cadherin and Vimentin expression in orthotopic tumor tissues (n = 5). Scale bar, 50 μm. **(C)** Representative images and the corresponding H&E staining of liver-metastatic foci. Mice were orthotopically transplanted with liver-metastatic CRC cells (CRC_{LM}) and TCAF2-knockdown TPC_{LM}. Scale bar, 1 cm (up); 2 mm (down). Quantification of the number and area of liver metastatic loci is shown (n = 6). Yellow and black dotted lines indicate liver metastatic foci. **(D)** Immunohistochemical analysis of E-cadherin, N-cadherin and Vimentin expression in primary tumor tissues (n = 5). Scale bar, 50 μm. Data are presented as mean ± SEM. $^{*}P < 0.05$, $^{**}P < 0.01$, $^{***}P < 0.001$ by two-tailed unpaired *t*-test. LM, liver metastasis; NM, non-metastasis; PDX, patient-derived xenograft.

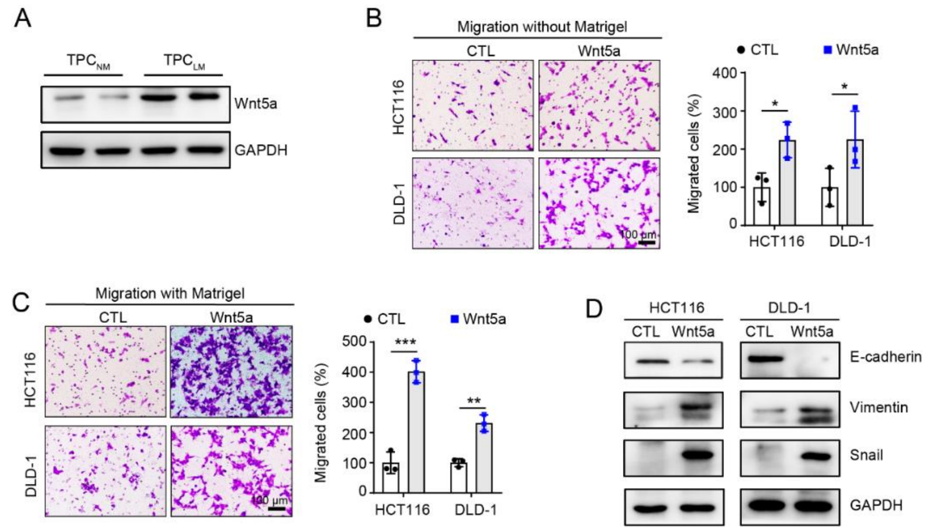

Figure S22. Wnt5a facilitates motility and EMT of CRC cells. (A) Western blot analysis of Wnt5a in TPC_{NM} and TPC_{LM}. **(B, C)** Transwell assay for the migration

without (**B**) or with Matrigel (**C**) of HCT116 and DLD-1 cells treated with Wnt5a (n = 3). Scale bar, 100 μm. **(D)** Western blot analysis of E-cadherin, Vimentin and Snail in HCT116 and DLD-1 cells treated with Wnt5a. Data are presented as mean ± SEM. $^{*}P < 0.05$, $^{**}P < 0.01$, $^{***}P < 0.001$ by two-tailed unpaired *t*-test. CTL, control.

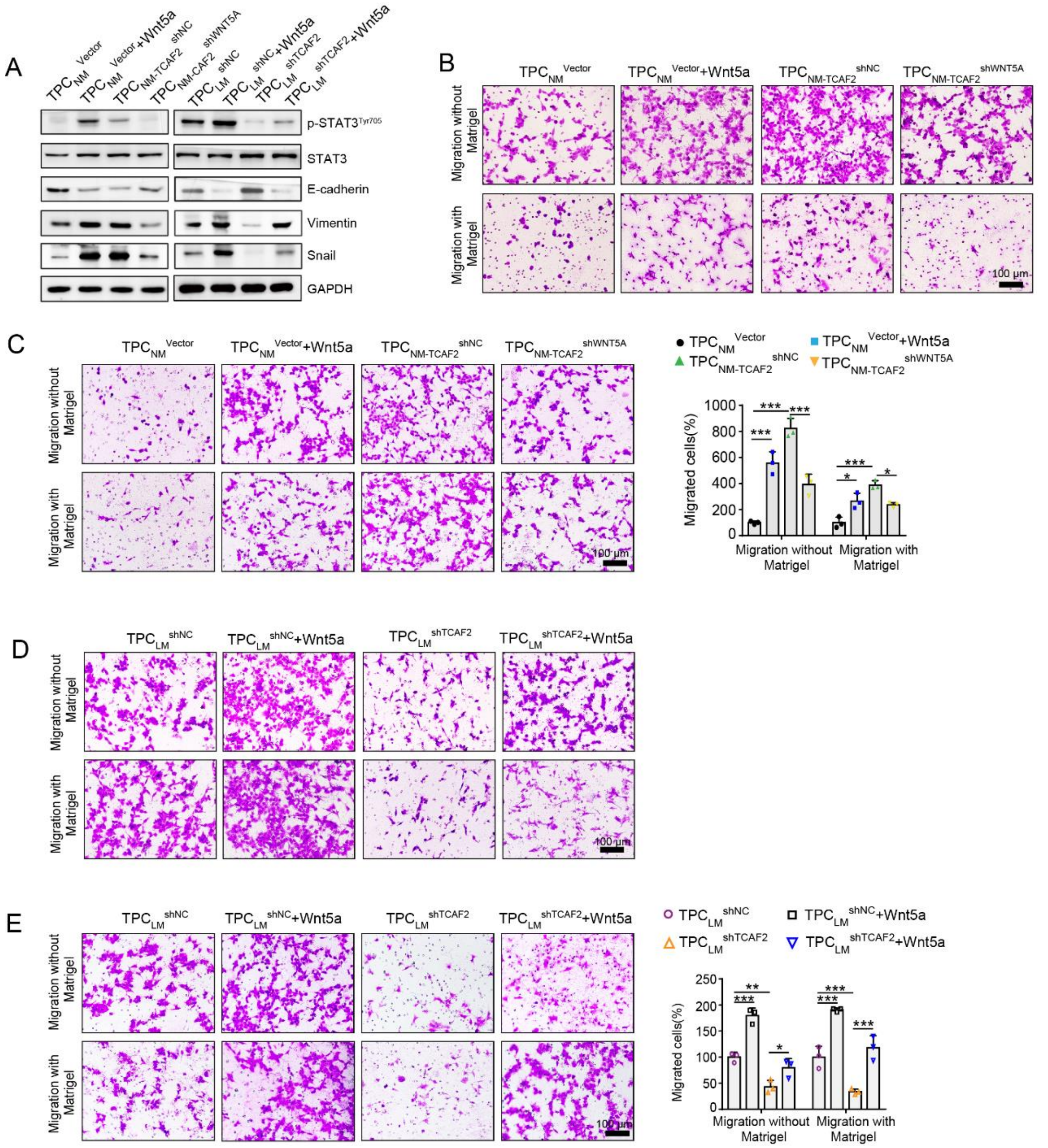

Figure S23. TCAF2 in TPCs enhances the motility and EMT of CRC cells via activating the Wnt5a/STAT3 signaling pathway. (A) Western blot analysis of p-$STAT3^{Tyr705}$, STAT3, and EMT markers in DLD-1 cells primed with the conditioned

medium from the indicated TPCs with or without Wnt5a. **(B, C)** Transwell assay for the migration with or without Matrigel of HCT116 (**B**) and DLD-1 cells (**C**) primed with the conditioned medium from the indicated TPCs treated with or without Wnt5a. Quantification of the migrated cells is shown (n = 3). Scale bar, 100 μm. **(D, E)** Transwell assay for the migration with or without Matrigel of HCT116 (**D**) and DLD-1 cells (**E**) primed with the conditioned medium from the indicated TPCs treated with or without Wnt5a. Quantification of the migrated cells is shown (n = 3). Scale bar, 100 μm. Data are presented as mean ± SEM. $^{*}P < 0.05$, $^{**}P < 0.01$, $^{***}P < 0.001$ by one-way ANOVA followed by Tukey's post hoc test. LM, liver metastasis; NM, non-metastasis; TPC, tumor pericyte.

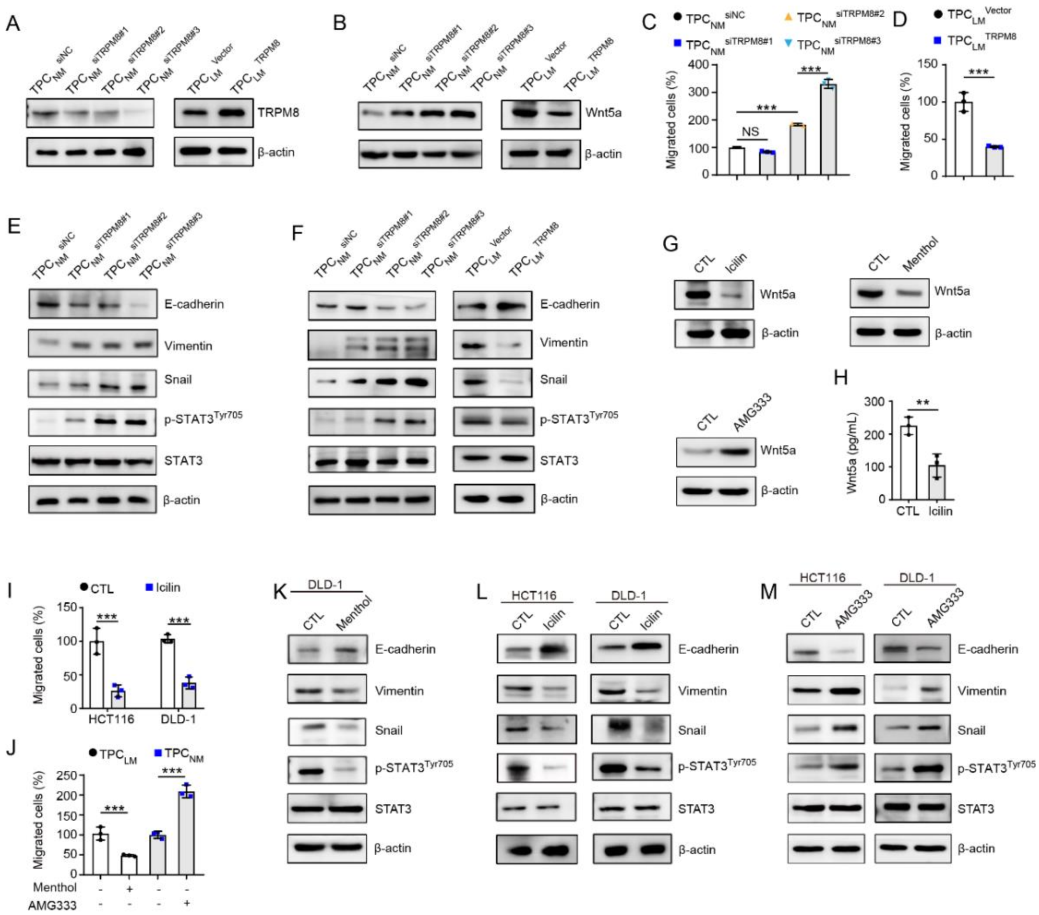

Figure S24. TCAF2 in TPCs induces Wnt5a/STAT3 signaling, EMT, and cell motility via TRPM8. (A, B) Western blot analysis of TRPM8 **(A)** and Wnt5a **(B)**

expression in TRPM8-knockdown or -overexpressing TPCs. **(C, D)** Transwell assay for migration of DLD-1 cells primed with the conditioned medium from TRPM8-knockdown **(C)** or -overexpressing TPCs **(D)**. Quantification of the migrated cells is shown (n = 3). Scale bar, 100 μm. **(E, F)** Western blot analysis for p-STAT3^{705}, STAT3, and EMT markers in HCT116 **(E)** and DLD-1 cells **(F)** primed with the conditioned medium from TRPM8-overexpressing or -knockdown TPCs. **(G)** Western blot analysis for Wnt5a expression in TPC_{LM} treated with icilin (30 μM) or menthol (100 μM), or TPC_{NM} treated with AMG333 (40 μM) for 6 h. **(H)** ELISA for Wnt5a secretion in the conditioned medium from TPCs treated with icilin (30 μM). **(I)** Transwell assay for the migration of HCT116 and DLD-1 cells primed with the conditioned medium from $TPC_{LM.}$ TPC_{LM} was pre-treated with or without icilin (30 μM) (n = 3). **(J)** Quantification of the migrated DLD-1 cells primed with the conditioned medium from TPCs pre-treated with or without menthol (100 μM) or AMG333 (40 μM) (n = 3). Scale bar, 100 μm. **(K-M)** Western blot analysis for p-STAT3^{Tyr705}, STAT3 and EMT markers in DLD-1 and HCT116 cells primed with the conditional medium from TPCs. TPC_{LM} were pre-treated with menthol (100 μM) or icilin (30 μM) for 6 h. TPC_{NM} were pre-treated with AMG333 (40 μM) for 6 h. Data are presented as mean ± SEM. NS, $^{**}P < 0.01$, $^{***}P < 0.001$ by one-way ANOVA followed by Tukey's post hoc test in **C**; by two-tailed unpaired *t*-test in **D**, **H**, **I**, **J**. CTL, control; LM, liver metastasis; NS, not significant; NM, non-metastasis; TPC, tumor pericyte.

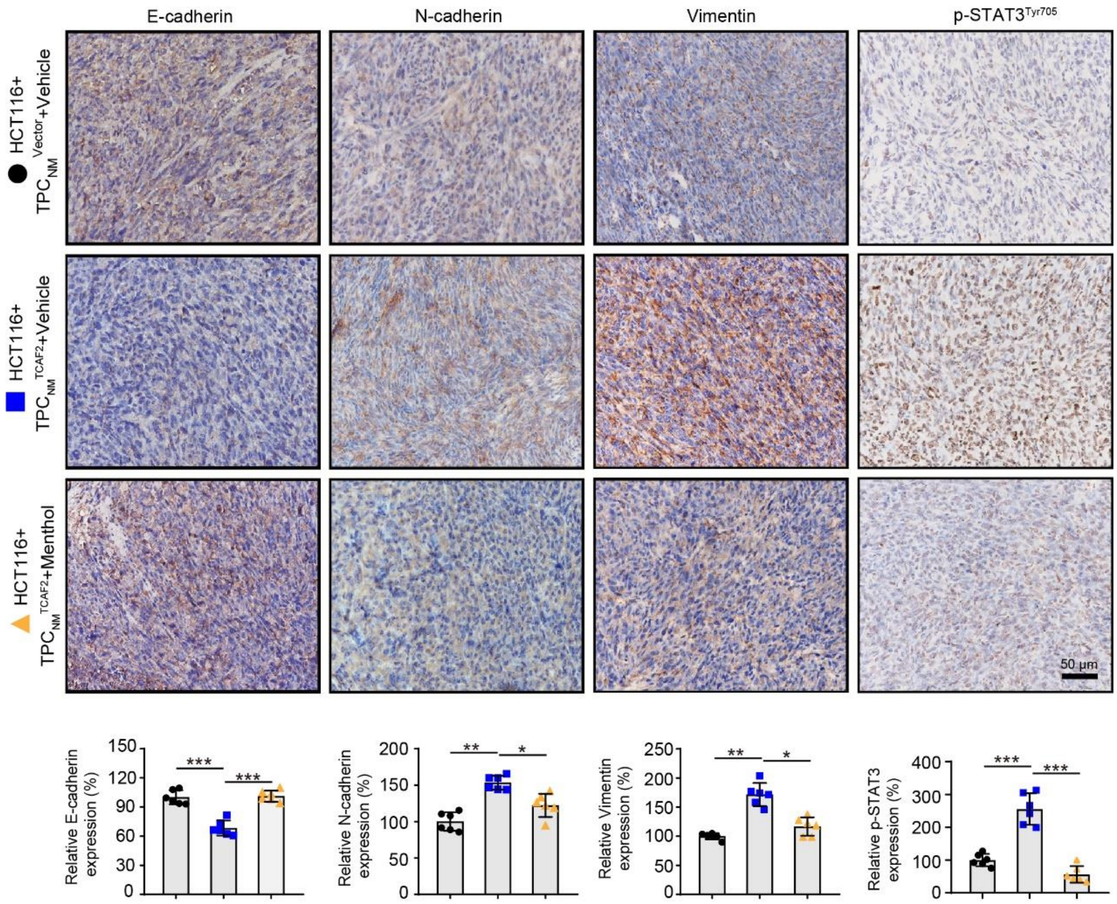

Figure S25. Menthol inhibits EMT and the expression of p-STAT3^{Tyr705} in the primary tumor tissues derived from HCT116 CRCLM xenografts. Immunohistochemical analysis of EMT makers and p-STAT3^{Tyr705} in orthotopic tumor tissues (n = 6). Scale bar, 50 μm. Data are presented as mean ± SEM. $^{*}P < 0.05$, $^{**}P < 0.01$, $^{***}P < 0.001$ by one-way ANOVA followed by Tukey's post hoc test. EMT, epithelial-mesenchymal transition; NM, non-metastasis; TPC, tumor pericyte.

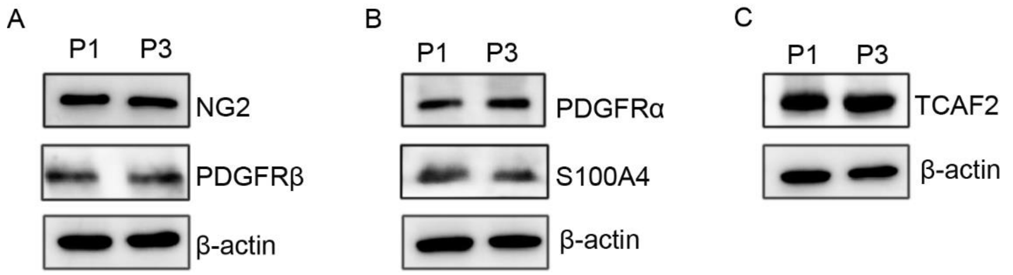

Figure S26. Comparisons of TPCs at P1 and P3. (A) Western blot analysis of NG2 and PDGFRβ in TPCs at P1 and P3. **(B)** Western blot analysis of PDGFRα and S100A4 in TPCs at P1 and P3. **(C)** Western blot analysis of TCAF2 in TPCs at P1 and P3. P1, passage 1; P3, passage 3.

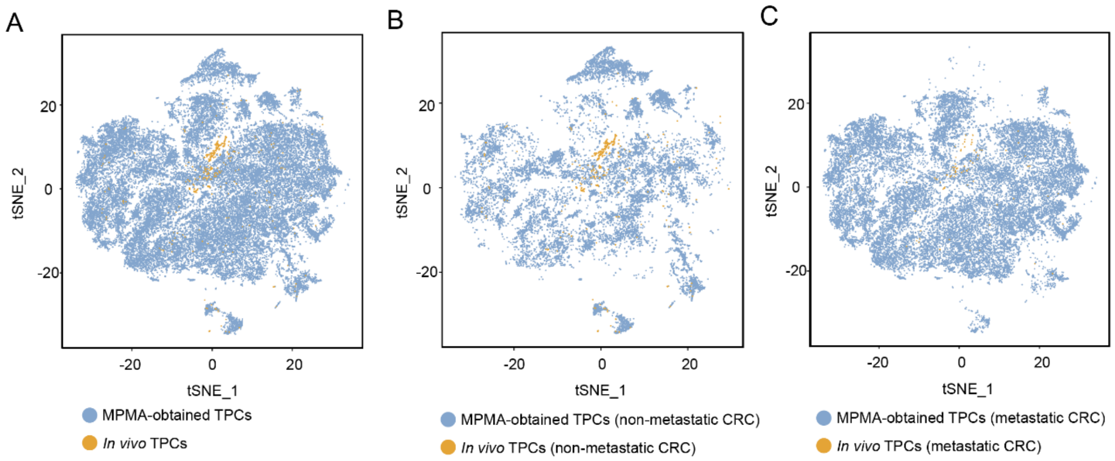

Figure S27. Comparation between the MPMA-obtained TPCs and the *in vivo* TPCs. **(A)** t-SNE visualization of the reported *in vivo* TPCs directly derived from primary tumor tissues of CRC patients and the MPMA-obtained TPCs. 56% of MPMA-obtained TPCs were overlapped with the reported *in vivo* TPCs **(B)** t-SNE visualization of the MPMA-obtained TPCs and the reported *in vivo* TPCs directly derived from non-metastatic CRC patients. 50% of the MPMA-obtained TPCs from non-metastatic CRC patients were overlapped with the reported *in vivo* TPCs directly derived from non-

metastatic CRC patients. **(C)** t-SNE visualization of the MPMA-obtained TPCs and the reported *in vivo* TPCs directly derived from liver metastatic CRC patients. 88% of the MPMA-obtained TPCs from metastatic CRC patients were overlapped with the reported *in vivo* TPCs derived from metastatic CRC patients. MPMA-obtained TPCs (P1) was analyzed by scRNA-seq in our previous study [6] (GEO accession: GSE199726) and the data of *in vivo* TPCs was derived from a published study on CRC [7] (GEO accession: GSE178341).

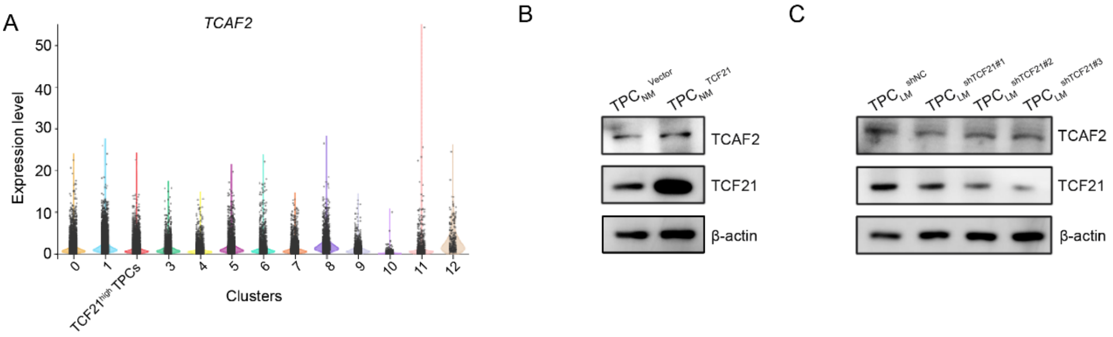

Figure S28. The correlation between TCAF2 and TCF21 in TPC. (A) Gene expression profiles of *TCAF2* in distinct subsets of TPCs. **(B, C)** Western blot analysis of TCAF2 in TCF21-overexpressing **(B)** or -knockdown TPCs **(C)**.

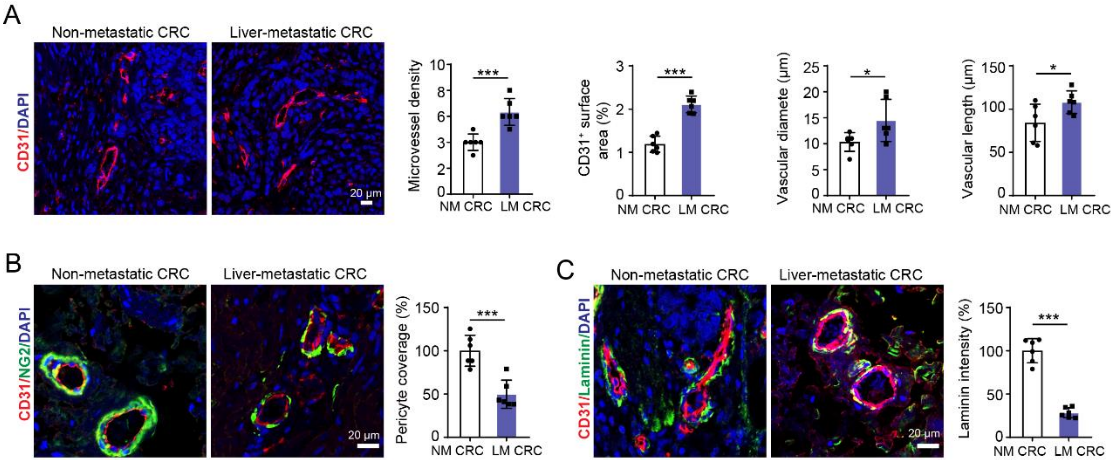

Figure S29. Vascular features in tumor tissues derived from CRC patients. (A) Immunofluorescence analysis and quantification of $CD31^+$ surface area, microvessel density, vascular diameter and vascular length in primary tumors derived from CRC patients with non-metastasis and liver metastasis (n = 6). Scale bar, 20 μm. **(B)** Immunofluorescence staining and quantification of $NG2^+$ (green) pericyte coverage of $CD31^+$ (red) blood vessels in primary tumors derived from CRC patients with non-metastasis and liver metastasis (n = 6). Scale bar, 20 μm. **(C)** Immunofluorescence staining and quantification of laminin (green) surrounding $CD31^+$ (red) blood vessels in primary tumor sections derived from CRC patients with or without liver metastasis (n = 6). Scale bar, 20 μm. Data are presented as mean ± SEM. $^{*}P < 0.05$, $^{***}P < 0.001$ by two-tailed unpaired *t*-test. CRC, colorectal cancer; NM CRC, non-metastatic colorectal cancer, LM CRC, liver-metastatic colorectal cancer.

References

[1] Q. Gao, H. Zhu, L. Dong, W. Shi, R. Chen, Z. Song, C. Huang, J. Li, X. Dong, Y. Zhou, Q. Liu, L. Ma, X. Wang, J. Zhou, Y. Liu, E. Boja, A. I. Robles, W. Ma, P. Wang, Y. Li, L. Ding, B. Wen, B. Zhang, H. Rodriguez, D. Gao, H. Zhou, J. Fan, *Cell* **2019**, *179*, 561.

[2] G. Yu, L. G. Wang, Y. Han, Q. Y. He, *OMICS* **2012**, *16*, 284.

[3] Y. M. Meng, X. Jiang, X. Zhao, Q. Meng, S. Wu, Y. Chen, X. Kong, X. Qiu, L. Su, C. Huang, M. Wang, C. Liu, P. P. Wong, *Nat. commun.* **2021**, *12*, 6011.

[4] B. M. Szczerba, F. Castro-Giner, M. Vetter, I. Krol, S. Gkountela, J. Landin, M. C. Scheidmann, C. Donato, R. Scherrer, J. Singer, C. Beisel, C. Kurzeder, V. Heinzelmann-Schwarz, C. Rochlitz, W. P. Weber, N. Beerenwinkel, N. Aceto, *Nature* 2019, **566**, 553.

[5] A. S. Harney, E. N. Arwert, D. Entenberg, Y. Wang, P. Guo, B. Z. Qian, M. H. Oktay, J. W. Pollard, J. G. Jones, J. S. Condeelis, *Cancer Discov* **2015**, *5*, 932.

[6] X. Li, J. Pan, T. Liu, W. Yin, Q. Miao, Z. Zhao, Y. Gao, W. Zheng, H. Li, R. Deng, D. Huang, S. Qiu, Y. Zhang, Q. Qi, L. Deng, M. Huang, P. M. Tang, Y. Cao, M. Chen, W. Ye, D. Zhang, *Gut* **2023**, *72*, 710.

[7] K. Pelka, M. Hofree, J. H. Chen, S. Sarkizova, J. D. Pirl, V. Jorgji, A. Bejnood, D. Dionne, W. H. Ge, K. H. Xu, S. X. Chao, D. R. Zollinger, D. J. Lieb, J. W. Reeves, C. A. Fuhrman, M. L. Hoang, T. Delorey, L. T. Nguyen, J. Waldman, M. Klapholz, I. Wakiro, O. Cohen, J. Albers, C. S. Smillie, M. S. Cuoco, J. Wu, M. J. Su, J. Yeung, B. Vijaykumar, A. M. Magnuson, N. Asinovski, T. Moll, M. N. Goder-Reiser, A. S.

Applebaum, L. K. Brais, L. K. Dellostritto, S. L. Denning, S. T. Phillips, E. K. Hill, J. K. Meehan, D. T. Frederick, T. Sharova, A. Kanodia, E. Z. Todres, J. Jané-Valbuena, M. Biton, B. Izar, C. D. Lambden, T. E. Clancy, R. Bleday, N. Melnitchouk, J. Irani, H. Kunitake, D. L. Berger, A. Srivastava, J. L. Hornick, S. Ogino, A. Rotem, S. Vigneau, B. E. Johnson, R. B. Corcoran, A. H. Sharpe, V. K. Kuchroo, K. Ng, M. Giannakis, L. T. Nieman, G. M. Boland, A. J. Aguirre, A. C. Anderson, O. Rozenblatt-Rosen, A. Regev, N. Hacohen, *Cell* **2021**, *184*, 4734.

Table S1. STR profiles of the MPMA-obtained TPCs.

Marker	Allele 1	Allele 2	Allele 3	Allele 4
D19S433	13	14		
D5S818	11	13		
D21S11	29	31.2		
D18S51	13	14		
D6S1043	12	13		
AMEL	X	Y		
D3S1358	16	17		
D13S317	10			
D7S820	8	12		
D16S539	9	11		
CSF1PO	12	14		
Penta D	9	12		
D2S441	10	11		
vWA	14	15		
D8S1179	10	14		
TPOX	8	10		
Penta E	11	12		
TH01	6	8		
D12S391	19	23		

D2S1338	22	23
FGA	20	23

Conclusion of cell identification: The results of STR typing showed that there were no multiple alleles at each locus. No cross contamination of human cells was found in TPCs.

Table S2. STR profiles of NG2$^+$ cells isolated by MACS.

**Marker**	**Allele 1**	**Allele 2**	**Allele 3**	**Allele 4**
D19S433	13	14.2	15	15.2
D5S818	10	11	12	
D21S11	30	31.2	32.2	
D18S51	13	15	18	
D6S1043	11	15	18	19
AMEL	X	Y		
D3S1358	15	16		
D13S317	8	10	11	
D7S820	8	11		
D1^{6}S539	9	11		
CSF1PO	10	11		
Penta D	9	12		
D2S441	9.1	10	14	
vWA	17	18		
D8S1179	10	12	14	15
TPOX	8	11		
Penta E	11	19	21	
TH01	7	8	9	
D12S391	15	19	21	24

D2S1338	19	20	23	25
FGA	21	22	24	25

Conclusion of cell identification: The cell STR typing results of this strain showed that D5S818, D21S11, D18S51, D13S317, D2S441, Penta E, TH01 showed the phenomenon of three isogenies, D19S433, D6S1043, D8S1179, D12S391, D2S1338, FGA, no multiple alleles at other loci. Cross-contamination of human cells was suspected.

Table S3. Correlation analysis between the TCAF2+ TPC ratio and the clinicopathologic data.

Character	TCAF2+ TPC ratio (≤30%)	TCAF2+ TPC ratio (>30%)	*P* value
**Gender**			
Female	21 (51.2%)	20 (48.8%)	0.168
Male	34 (65.4%)	18 (34.6%)	
**Age**			
＜60	15 (53.6%)	13 (46.4%)	0.551
≥60	40 (61.5%)	25 (38.5%)	
**TNM stage**			
I-II	35 (89.7%)	4 (10.3%)	< 0.001
IV	20 (37.0%)	34 (63.0%)	
**Liver metastasis**			
No	35 (89.7%)	4 (10.3%)	< 0.001
Yes	20 (37.0%)	34 (63.0%)	
KRAS mutation			
No	48 (57.1%)	36 (42.9%)	0.231
Yes	7 (77.8%)	2 (22.2%)	
BRAF mutation			

No	43 (59.7%)	29 (40.3%)	0.832
Yes	12 (57.1%)	9 (42.9%)	

Table S4. Clinical characteristics of non-metastatic CRC specimens

**Characteristics**	**Case 1**	**Case 2**	**Case 3**	**Case 4**	**Case 5**	**Case 6**
**Gender/Age (yr)**	Male (58)	Male (78)	Male (59)	Female (53)	Male (73)	Female (75)
**Date of diagnosis**	20190429	20191031	20200331	20210323	20210402	20210420
**Tumor type**	Colorectal Adenocarcinoma	Colorectal Adenocarcinoma	Colon Adenocarcinoma	Colon Adenocarcinoma	Colon Adenocarcinoma	Colon Adenocarcinoma
**Location**	Rectum	Sigmoid colon	Sigmoid colon	Sigmoid colon	Sigmoid colon	transverse colon
**Tumor size (maximum diameter)**	5 cm	4 cm	2 cm	4 cm	5 cm	5 cm
**Differentiation**	Moderate	Moderate	Moderate	Moderate	Moderate	Moderate
**TNM stage**	T2N0M0	T4aN0M0	T3N1bM0	T4aN1bM0	T3N0M0	T3N1aM0
**Clinical stage**	I	IIB	IIIB	IIIB	IIA	IIIB
**Clinical metastasis**	No metastasis	No metastasis	No metastasis	No metastasis	No metastasis	No metastasis
**Treatment status**	chemotherapy radiotherapy	No treatment before surgery	No treatment before surgery	No treatment before surgery	No treatment before surgery	No treatment before surgery

Table S5. Clinical characteristics of liver-metastatic CRC specimens

Characteristics	Case 1	Case 2	Case 3	Case 4	Case 5	Case 6
**Gender/Age (yr)**	Female (61)	Male (51)	Male (78)	Male (64)	Female (51)	Female (59)
**Date of diagnosis**	20210324	20190516	20191010	20190103	20190626	20200320
**Tumor type**	Rectal Adenocarcinoma	Colorectal Adenocarcinoma	Colorectal Adenocarcinoma	Colorectal Adenocarcinoma	Colorectal Adenocarcinoma	Colorectal Adenocarcinoma
**Location**	Rectum	Descending colon and ileum	Sigmoid colon	Sigmoid colon	Descending colon and ileum	Descending colon and ileum
**Tumor size (maximum diameter)**	6 cm	4 cm	6 cm	5 cm	6 cm	7 cm
**Differentiation**	Moderate	Moderate	Low	Low	Moderate	Low
**TNM stage**	T3N2M1a	T4bN1M1a	T3N2bM1a	T3N2M1a	T4bN1M1a	T4bN1aM1a
**Clinical stage**	Ⅳa	Ⅳa	Ⅳa	Ⅳa	Ⅳa	Ⅳa
**Clinical metastasis**	Liver metastasis	Liver metastasis	Liver metastasis	Liver metastasis	Liver metastasis	Liver metastasis
**Treatment status**	Chemotherapy radiotherapy	No treatment before surgery	No treatment before surgery	No treatment before surgery	No treatment before surgery	No treatment before surgery

Table S6. Clinical characteristics of CRC patient for PDX models

**Characteristics**	**Case 1**	**Case 2**
**Gender/Age (yr)**	Male (68)	Male (66)
**Date of diagnosis**	20220529	20220501
**Tumor type**	Colorectal Adenocarcinoma	Colorectal Adenocarcinoma
**Location**	Rectum	Sigmoid colon
**Tumor size (maximum diameter)**	5 cm	4 cm
**Differentiation**	Moderate	Moderate
**TNM stage**	T2N1M1a	T4aN0M0
**Clinical stage**	IVa	IIB
**Clinical metastasis**	metastasis	No metastasis
**Treatment status**	No treatment before surgery	No treatment before surgery

Table S7. Clinical characteristics of CRC specimens.

Character	Overall population
**Gender, number of patients (%)**	
Male	52 (55.9)
Female	41 (44.1)
**Age, median (range)**	67 (40 – 90)
**TNM stage, number of patients (%)**	
I-II	39 (41.9)
IV	54 (58.1)
**Liver metastasis (%)**	
No	39 (41.9)
Yes	54 (58.1)
KRAS mutation (%)	
No	84 (90.3)
Yes	9 (9.7)
BRAF mutation (%)	
No	72 (77.4)
Yes	21 (22.6)

Table S8. Clinical characteristics of breast cancer specimens.

Character	Overall population
**Gender, number of patients (%)**	
Male	0 (0)
Female	20 (100)
**Age, median (range)**	57 (30 –82)
**TNM stage, number of patients (%)**	
I-II	10 (50)
IV	10 (50)
**pulmonary metastasis (%)**	
No	10 (50)
Yes	10 (50)
**ER status** (%)	
Positive	15 (75)
Negative	5 (25)
**PR status** (%)	
Positive	14 (70)
Negative	6 (30)
**HER status** (%)	
Positive	11 (55)
Negative	9 (45)

Table S9. List of antibodies for flow cytometry.

Antibody	Catalog	Source
CD31-PE	Cat. 303106	Biolegend
CD326-PE	Cat. 369806	Biolegend
NG2-PE	Cat. 130-097-458	Miltenyi Biotec
CD146-PE	Cat. 130-097-939	Miltenyi Biotec
CD105-PE	Cat. 323205	Biolegend
CD140b-PE	Cat. 323606	Biolegend
CD140b-APC	Cat. 323608	Biolegend
CD44-PE	Cat. 397503	Biolegend

Table S10. List of antibodies for immunohistochemistry and immunofluorescence.

Antibody	Catalog	Source
E-Cadherin	Cat. 3195S	Cell Signaling Technology
N-Cadherin	Cat. ab18203	Abcam
Vimentin	Cat. 5741S	Cell Signaling Technology
Wnt5a	Cat. 55184-1-AP	Proteintech
p-STAT3^{Tyr705}	Cat. 9145S	Cell Signaling Technology
CAIX	Cat. ab15086	Abcam
TCAF2	Cat. HPA038756	Sigma
TRPM8	Cat. BM5383	BOSTER
Human CD31	Cat. GB11063	Servicebio
Mouse CD31	Cat. AF3628	R&D Systems
NG2	Cat. ab183929	Abcam
PDGFRβ	Cat. ab32570	Abcam
α-SMA	Cat. 48938	Cell Signaling Technology
FAPα	Cat. AF3715	R&D Systems
Laminin	Cat. ab11575	Abcam
anti-mouse IgG, HRP-linked Antibody	Cat. 7076s	Cell Signaling Technology
anti-rabbit IgG, HRP-linked Antibody	Cat. 7074s	Cell Signaling Technology

anti-goat IgG, HRP-linked Antibody	Cat. A0181	Beyotime
anti-sheep IgG, HRP-linked Antibody	Cat. ab6900	Abcam
iF488-Tyramide	Cat. G1231	Servicebio
iF555-Tyramide	Cat. G1233	Servicebio
iF647-Tyramide	Cat. G1232	Servicebio
Donkey anti-Goat IgG (H+L) Cross-Adsorbed Secondary Antibody, Alexa Fluor™ 647	Cat. A21447	Thermo Scientific

Table S11. List of antibodies for Western blot.

Antibody	Catalog	Source
E-Cadherin	Cat. 3195S	Cell Signaling Technology
N-Cadherin	Cat. ab18203	Abcam
Vimentin	Cat. 5741S	Cell Signaling Technology
Snail	Cat. 3879S	Cell Signaling Technology
TCAF2	Cat. HPA038756	Sigma
TRPM8	Cat. BM5383	BOSTER
STAT3	Cat. 12640S	Cell Signaling Technology
p-STAT3^{Tyr705}	Cat. 9145S	Cell Signaling Technology
HIF1α	Cat. 36169s	Cell Signal Technology;
PDGFRα	Cat. 3174S	Cell Signaling Technology
S100A4	Cat. 13018S	Cell Signaling Technology
NG2	Cat. ab183929	Abcam
PDGFRβ	Cat. ab32570	Abcam
COL1A2	Cat. ab308455	Abcam
COL3A1	Cat. AF6531	Beyotime
Decorin	Cat. ab277636	Abcam
GAPDH	Cat. 5174S	Cell Signaling Technology
β-actin	Cat. 4970S	Cell Signaling Technology

anti-mouse IgG, HRP-linked Antibody	Cat. 7076s	Cell Signaling Technology
anti-rabbit IgG, HRP-linked Antibody	Cat. 7074s	Cell Signaling Technology

Table S12. The primers used in this study.

**Genes**	**Forward （5’-3’）**	**Reverse （5’-3’）**
ACTB	GTTGCTATCCAGGCTGTGCTATCC	GGTGGCAGTGATGGCATGGAC
TCAF2	ACCACGAGAATGGGAACTTG	GAGCCTGTGCAGGGATATGT
FYTTD1	TGCCTGAGTCCCTCACAAC	ACAATCTCACCGATCCTCTGC
TMX4	ATCCCCGGCTTCTCTAACGA	AACCAAGGTGGCTATGACGA
SMA7A	TTCAGCCCGGACGAGAACT	GAACCGAGGGATCTTCCCAT
SP110	CTTCCTATGAACGGCAGAGC	GGCGACTCACTCAGGATCTC
GSTT1	TTCCTTACTGGTCCTCACATCTC	TCACCGGATCATGGCCAGCA
USP33	CTTGCTGCCTTCTTTGCCAG	TTTTTGTGCCTCTTCGCTGC
IGF2	AGACCCTTTGCGGTGGAGA	GGAAACATCTCGCTCGGACT
SLC16A6	GACACCGTCTGGTAGTGATGT	TGAGAAACCTCTTGTGAGAAGGA
NCAM1	AGCTCCGCGTCTACAAAGC	CTACACAGGTAGCGACCTCC
NBR1	TGGGCAAATGAGTGTGTGTG	TTGCCCCTGTCCAAGTTTTG
COPZ2	AGATAATGACGGGCGAAGGC	AAACAGGCAGGCAAGCACAGAC
FBLN2	ATCATGGCGGATGGTGTGTC	CCCATGAGGCACTCGTCTTG

PID1	GAGGATGTCTTTCCGGCCAA	AGACCCAGGCGAAGATGTTG
PLIN2	CACAACCGAGTGTGGTGACT	CACACCGTTCTCTGCCATCT
CDA	CCGTCTCAGAAGGGTACAAG	GACAATATACGTACCATCCG G
ACSS1	CCGATCAGGTCCTGGTAGTG A	CTCGGCCCATGACAATCTTC
PHYHD1	TGCCTGAGTCCCTCACAAC	ACAATCTCACCGATCCTCTG C
CRBN	AGCATGGTGCGGAACTTAAT C	ATCTCTGCTGTTGTCCCAAAC
SERPINF1	AAAAAGCTCTGTGCTGGCTG	TCCAATGCAGAGGAGTAGCA C

Table S13. Sequence of siRNAs**.**

**SiRNA**	**Sense （5’-3’）**	**Antisense （5’-3’）**
siTRPM 8-1	GCACUAUUCAAGAUUCCAATT	UUGGAAUCUUGAAUAGUGCTT
siTRPM 8-2	GGAGUCUGCUGACCUUCAATT	UUGAAGGUCAGCAGACUCCTT
siTRPM 8-3	CCUCACGUUUGUCUGGAAATT	UUUCCAGACAAACGUGAGGTT
siHIF1A	GCUGAUUUGUGAACCCAUUTT	AAUGGGUUCACAAAUCAGCTT

Uncropped Western blot bands:

Figure 2

Figure 2C E-cadherin (135 kDa, left)

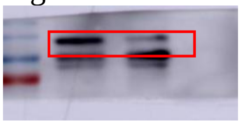

Figure 2C E-cadherin (135 kDa, right)

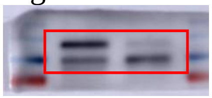

Figure 2C N-cadherin (100kDa, left)

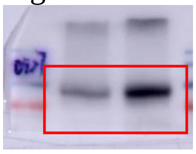

Figure 2C N-cadherin (100 kDa, right)

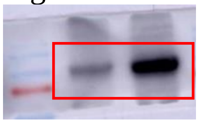

Figure 2C Vimentin (57 kDa, left)

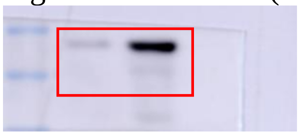

Figure 2C Vimentin (57 kDa, right)

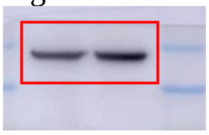

Figure 2C Snail (29 kDa, left)

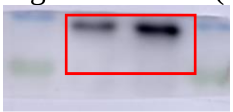

Figure 2C Snail (29 kDa, right)

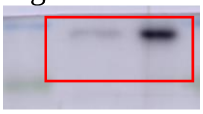

Figure 2C GAPDH (37 kDa, left)

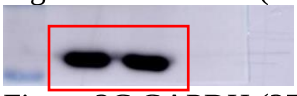

Figure 2C GAPDH (37 kDa, right)

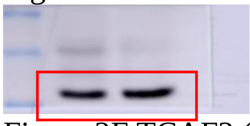

Figure 2F TCAF2 (100 kDa)

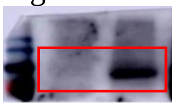

Figure 2F GAPDH (37 kDa)

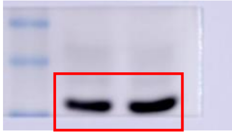

Figure 4

Figure 4B Wnt5a (42 kDa)

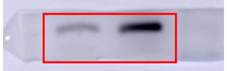

Figure 4B GAPDH (37 kDa)

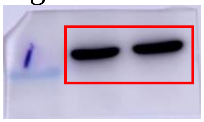

Figure 4D Wnt5a (42 kDa)

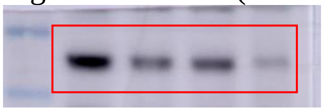

Figure 4D GAPDH (37 kDa)

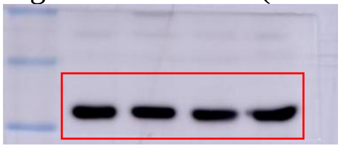

Figure 4L p-STAT3^{Ty705} (79 kDa, left)

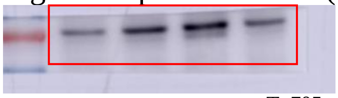

Figure 4L p-STAT3 ^{Ty705} (79 kDa, right)

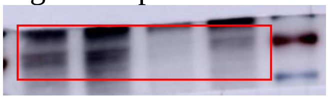

Figure 4L STAT3 (79 kDa, left)

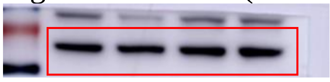

Figure 4L STAT3(79 kDa, right)

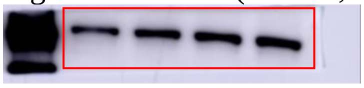

Figure 4L E-cadherin (135 kDa, left)

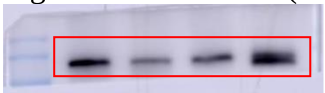

Figure 4L E-cadherin (135 kDa, right)

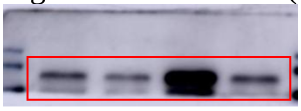

Figure 4L Vimentin (57 kDa, left)

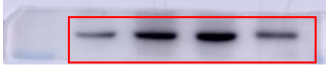

Figure 4L Vimentin (57 kDa, right)

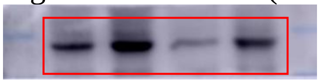

Figure 4L Snail (29 kDa, left)

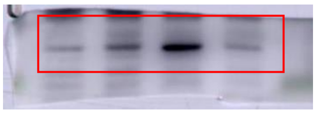

Figure 4L Snail (29 kDa, right)

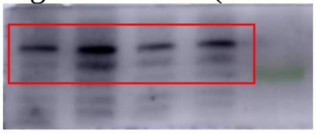

Figure 4L GAPDH (37 kDa, left)

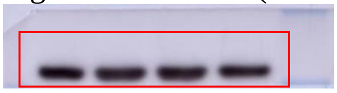

Figure 4L GAPDH (37 kDa, right)

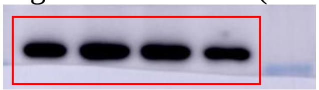

Figure 5

Figure 5D TRPM8 (127 kDa)

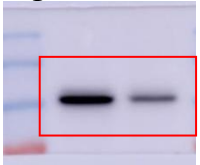

Figure 5D GAPDH (37 kDa)

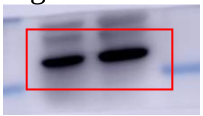

Figure 5E TRPM8 (127 kDa)

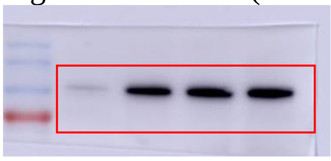

Figure 5E GAPDH (37 kDa)

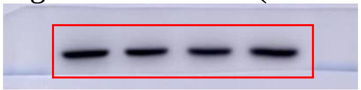

Figure 6

Figure 6C E-cadherin (135 kDa)

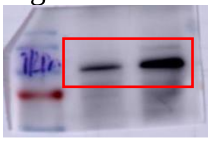

Figure 6C Vimentin (57 kDa)

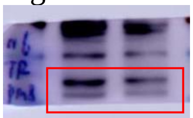

Figure 6C Snail (29 kDa)

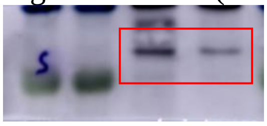

Figure 6C p-STAT3^{Tyr705} (79 kDa)

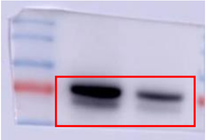

Figure 6C STAT3(79 kDa)

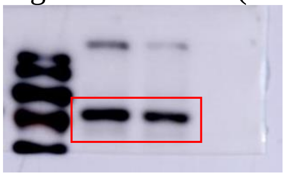

Figure 6C GAPDH (37 kDa)

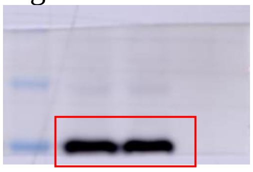

Figure 6F E-cadherin (135 kDa)

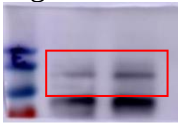

Figure 6F Vimentin (57 kDa)

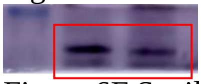

Figure 6F Snail (29 kDa)

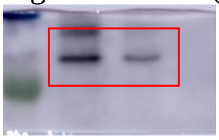

Figure 6F p-STAT3 ^{Tyr705} (79 kDa)

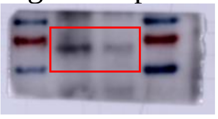

Figure 6F STAT3(79 kDa)

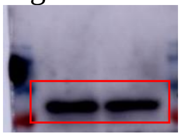

Figure 6F GAPDH (37 kDa)

-

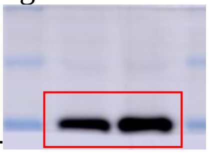

Figure S4

Figure S4 PDGFRα (190 kDa)

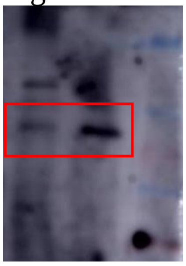

Figure S4 S100A4 (12kDa)

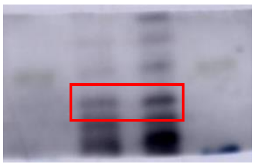

Figure S4 COL1A2 (220 kDa)

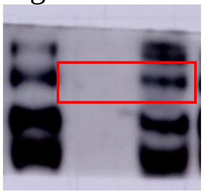

Figure S4 COL3A1 (200 kDa)

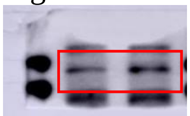

Figure S4 Decorin (40 kDa)

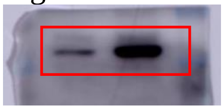

Figure S4 β-actin (42 kDa)

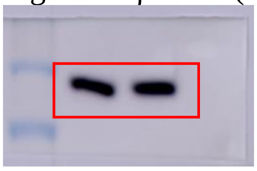

Figure S5

Figure S5H E-cadherin (135 kDa, left)

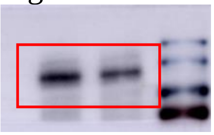

Figure S5H E-cadherin (135 kDa, right)

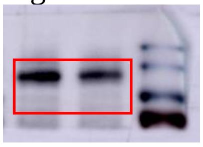

Figure S5H N-cadherin (100kDa, left)

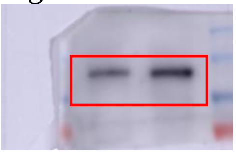

Figure S5H N-cadherin (100 kDa, right)

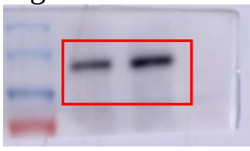

Figure S5H Vimentin (57 kDa, left)

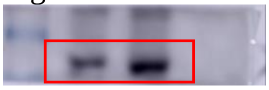

Figure S5H Vimentin (57 kDa, right)

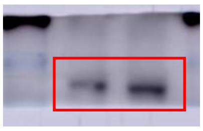

Figure S5H Slug (29 kDa, left)

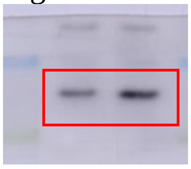

Figure S5H Slug (29 kDa, right)

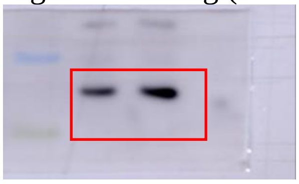

Figure S5H β-actin (42 kDa, left)

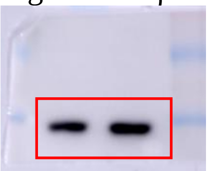

Figure S5H β-actin (42 kDa, right)

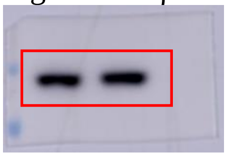

Figure S9

Figure S9 TCAF2 (100 kDa)

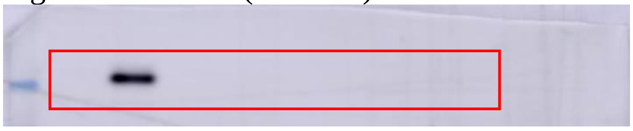

Figure S9 GAPDH (37 kDa)

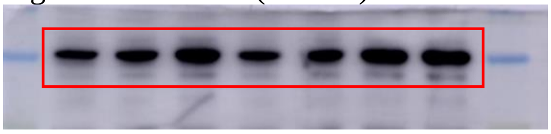

Figure S10

Figure S10D HIF-1α (120 kDa)

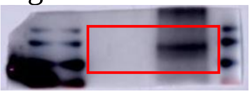

Figure S10D TCAF2 (100 kDa)

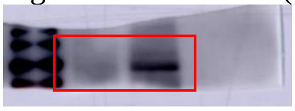

Figure S9D β-actin (42 kDa)

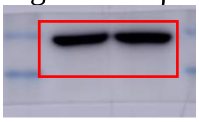

Figure S10E HIF-1α (120 kDa, left)

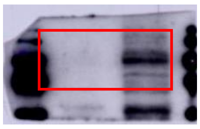

Figure S10E HIF-1α (120 kDa, right)

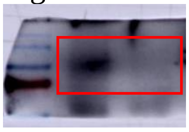

Figure S10E TCAF2 (100 kDa, left)

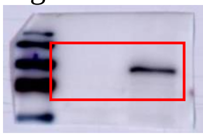

Figure S10E TCAF2 (100 kDa, right)

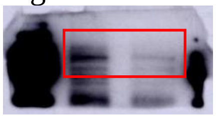

Figure S10E β-actin (42 kDa, left)

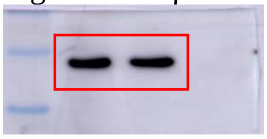

Figure S10E β-actin (42 kDa, right)

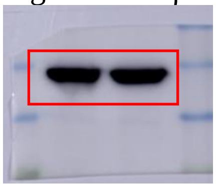

Figure S11

Figure S11B TCAF2 (100 kDa)

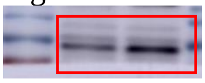

Figure S11B GAPDH (37 kDa)

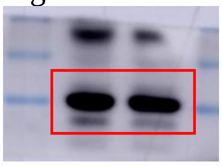

Figure S11D TCAF2 (100 kDa)

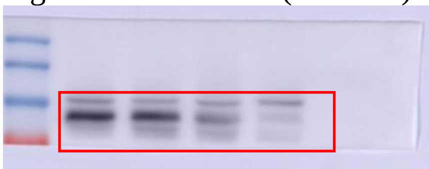

Figure S11D GAPDH (37 kDa)

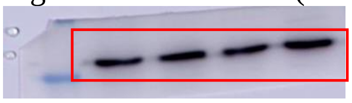

Figure S14

Figure S14E E-cadherin (135 kDa, left)

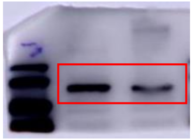

Figure S14E E-cadherin (135 kDa, right)

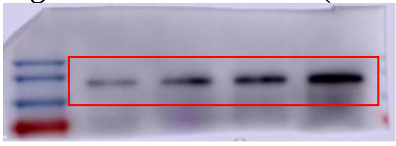

Figure S14E Vimentin (57 kDa, left)

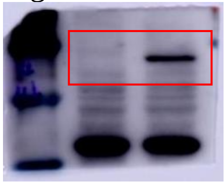

Figure S14E Vimentin (57 kDa, right)

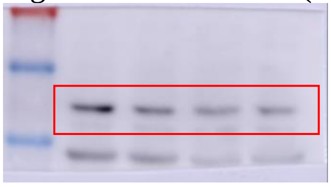

Figure S14E Snail (29 kDa, left)

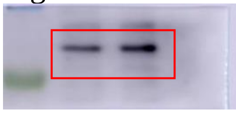

Figure S14E Snail (29 Da, right)

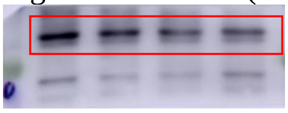

Figure S14E GAPDH (37 kDa, left)

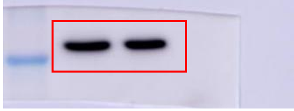

Figure S14E GAPDH (37 kDa, right)

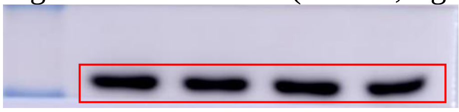

Figure S14F E-cadherin (135 kDa, left)

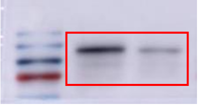

Figure S14F E-cadherin (135 kDa, right)

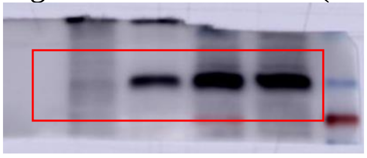

Figure S14F Vimentin (57 kDa, left)

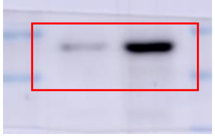

Figure S14F Vimentin (57 kDa, right)

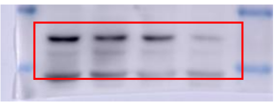

Figure S14F Snail (29 kDa, left)

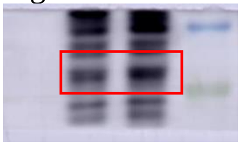

Figure S14F Snail (29 kDa, right)

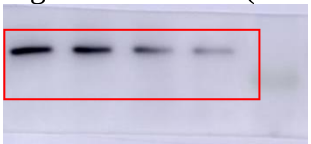

Figure S14F GAPDH (37 kDa, left)

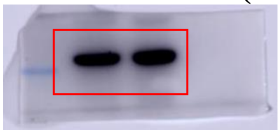

Figure S14F GAPDH (37 kDa, right)

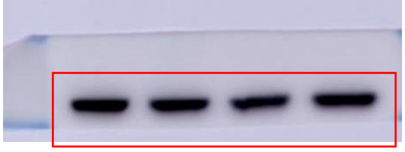

Figure S22

Figure S22A Wnt5a (42 kDa)

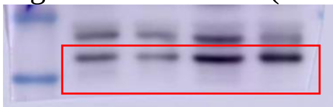

Figure S22A GAPDH (37 kDa)

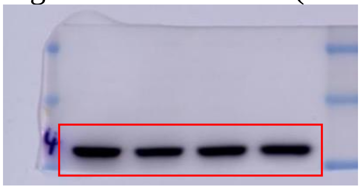

Figure S22D E-cadherin (135 kDa, left)

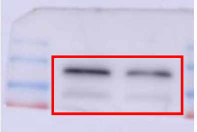

Figure S22D E-cadherin (135 kDa, right)

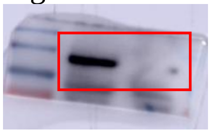

Figure S22D Vimentin (57 kDa, left)

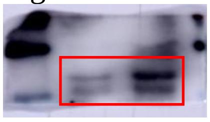

Figure S22D Vimentin (57 kDa, right)

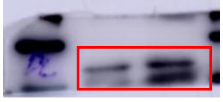

Figure S22D Snail (29 kDa, left)

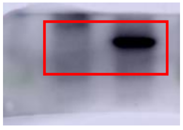

Figure S22D Snail (29 kDa, right)

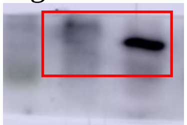

Figure S22D GAPDH (37 kDa, left)

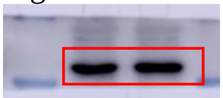

Figure S22D GAPDH (37 kDa, right)

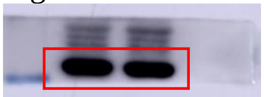

Figure S23

Figure S23A p-STAT3^{Tyr705} (79 kDa, left)

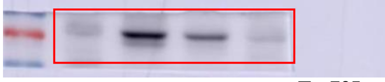

Figure S23A p-STAT3^{Tyr705} (79 kDa, right)

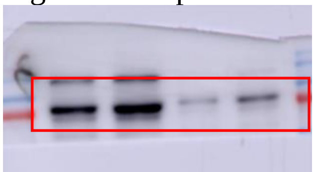

Figure S23A STAT3(79 kDa, left)

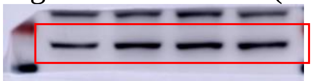

Figure S23A STAT3(79 kDa, right)

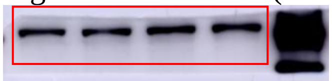

Figure S23A E-cadherin (135 kDa, left)

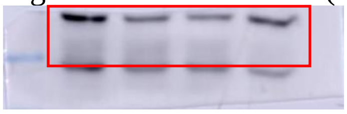

Figure S23A E-cadherin (135 kDa, right)

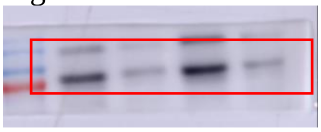

Figure S23A Vimentin (57 kDa, left)

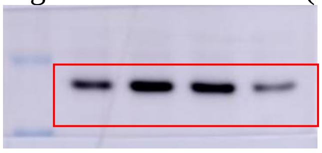

Figure S23A Vimentin (57 kDa, right)

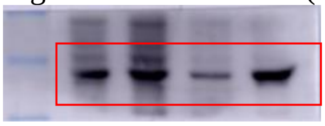

Figure S23A Snail (29 kDa, left)

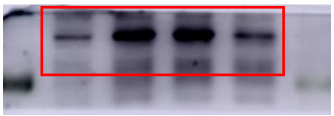

Figure S23A Snail (29 kDa, right)

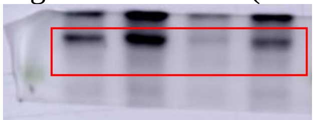

Figure S23A GAPDH 37 kDa, left)

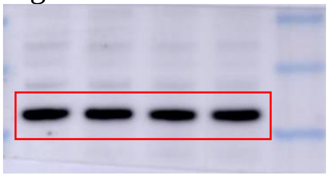

Figure S23A GAPDH 37 kDa, right)

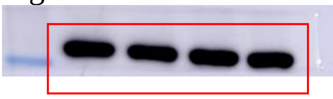

Figure S24

Figure S24A TRPM8 (127 kDa, left)

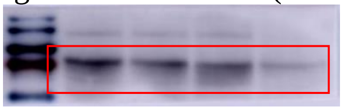

Figure S24A TRPM8 (127 kDa, right)

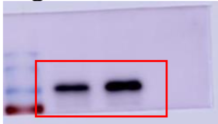

Figure S24A β-actin (42 kDa, left)

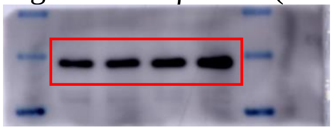

Figure S24A β-actin (42 kDa, right)

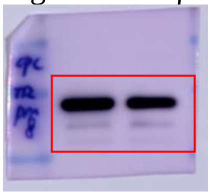

Figure S24B Wnt5a (42 kDa, left)

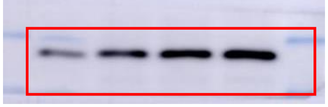

Figure S24B Wnt5a (42 kDa, right)

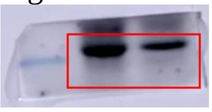

Figure S24B β-actin (42 kDa, left)

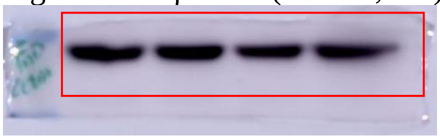

Figure S24B β-actin (42 kDa, right)

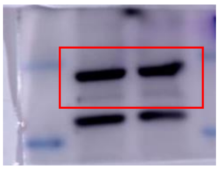

Figure S24E E-cadherin (135 kDa)

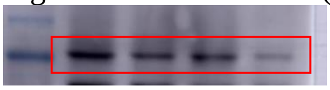

Figure S24E Vimentin (57 kDa)

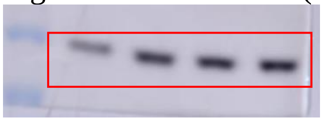

Figure S24E Snail (29 kDa)

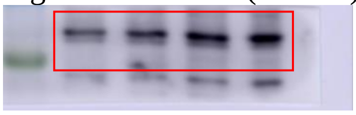

Figure S24E p-STAT3^{Tyr705} (79 kDa)

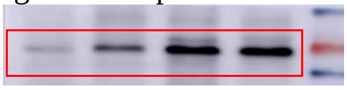

Figure S24E STAT3 (79 kDa)

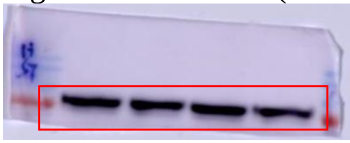

Figure S24E β-actin (42 kDa)

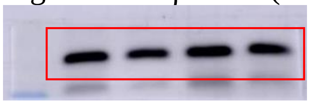

Figure S24F E-cadherin (135 kDa, left)

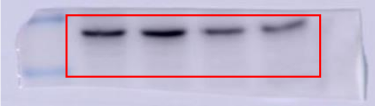

Figure S24F E-cadherin (135 kDa, right)

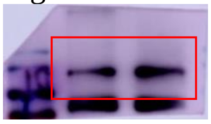

Figure S24F Vimentin (57 kDa, left)

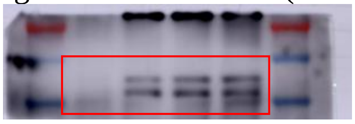

Figure S24F Vimentin (57 kDa, right)

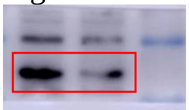

Figure S24F Snail (29 kDa, left)

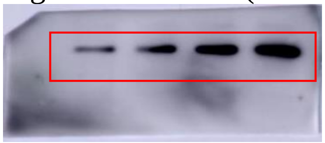

Figure S24F Snail (29 kDa, right)

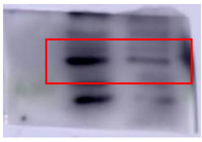

Figure S24F p-STAT3^{Tyr705} (79 kDa, left)

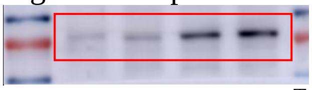

Figure S24F p-STAT3^{Tyr705} (79 kDa, right)

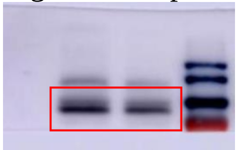

Figure S24F STAT3 (79 kDa, left)

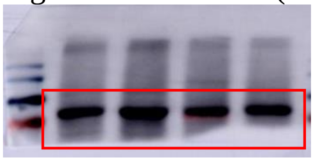

Figure S24F STAT3 (79 kDa, right)

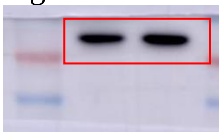

Figure S24F β-actin (42 kDa, left)

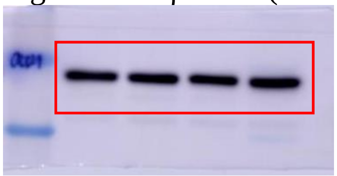

Figure S24F β-actin (42 kDa, right)

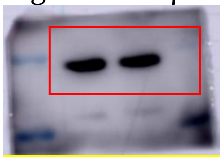

Figure S24G Wnt5a (42 kDa, left)

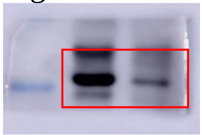

Figure S24G Wnt5a (42 kDa, right)

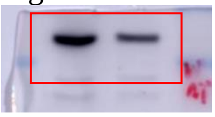

Figure S24G β-actin (42 kDa, left)

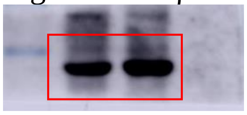

Figure S24G β-actin (42 kDa, right)

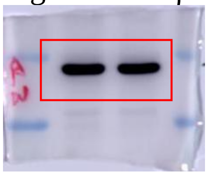

Figure S24G Wnt5a (42 kDa)

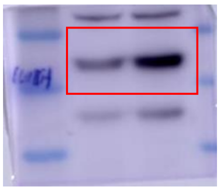

Figure S24G β-actin (42 kDa)

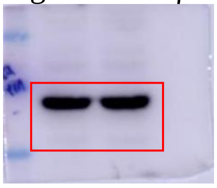

Figure S24K E-cadherin (135 kDa)

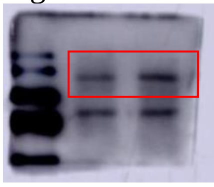

Figure S24K Vimentin (57 kDa)

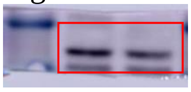

Figure S24K Snail (29 kDa)

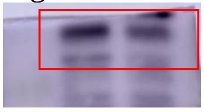

Figure S24K p-STAT3^{Tyr705} (79 kDa)

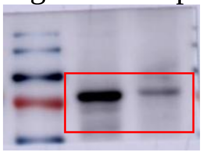

Figure S24K STAT3 (79 kDa)

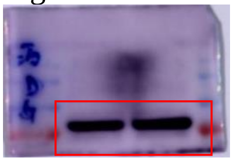

Figure S24K β-actin (42 kDa)

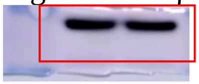

Figure S24L E-cadherin (135 kDa, left)

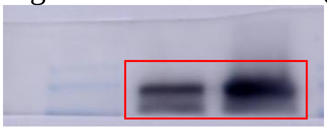

Figure S24L E-cadherin (135 kDa, right)

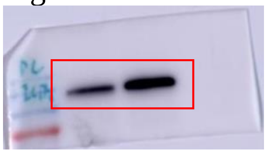

Figure S24L Vimentin (57 kDa, left)

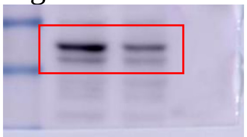

Figure S24L Vimentin (57 kDa, right)

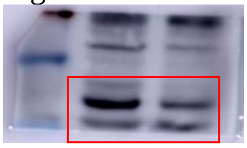

Figure S24L Snail (29 kDa, left)

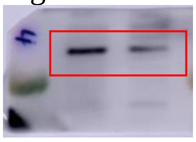

Figure S24L Snail (29 kDa, right)

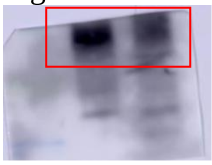

Figure S24L p-STAT3^{Tyr705} (79 kDa, left)

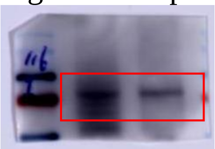

Figure S24L p-STAT3^{Tyr705} (79 kDa, right)

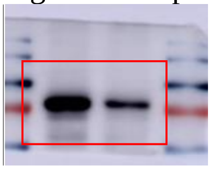

Figure S24L STAT3 (79 kDa, left)

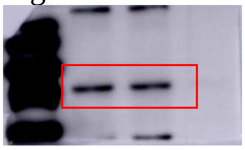

Figure S24L STAT3 (79 kDa, right)

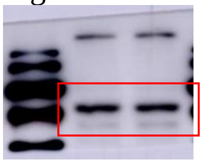

Figure S24L β-actin (42 kDa, left)

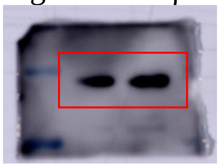

Figure S24L β-actin (42 kDa, right)

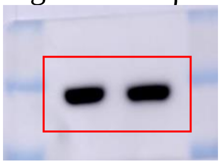

Figure S24M E-cadherin (135 kDa, left)

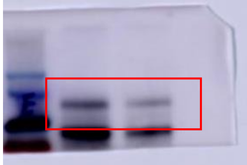

Figure S24M E-cadherin (135 kDa, right)

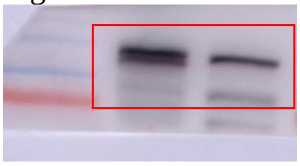

Figure S24M Vimentin (57 kDa, left)

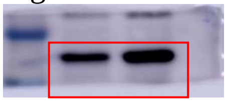

Figure S24M Vimentin (57 kDa, right)

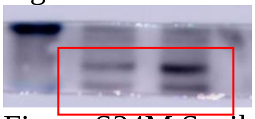

Figure S24M Snail (29 kDa, left)

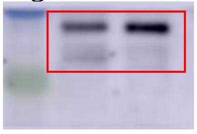

Figure S24M Snail (29 kDa, right)

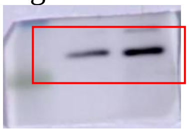

Figure S24M p-STAT3^{Tyr705} (79 kDa, left)

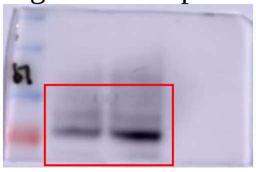

Figure S24M p-STAT3^{Tyr705} (79 kDa, right)

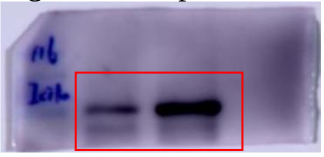

Figure S24M STAT3 (79 kDa, left)

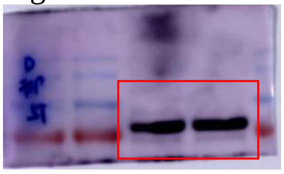

Figure S24M STAT3 (79 kDa, right)

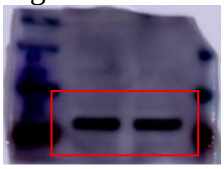

Figure S24M β-actin (42 kDa, left)

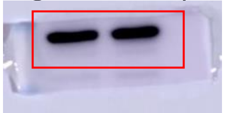

Figure S24M β-actin (42 kDa, right)

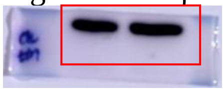

Figure S26

Figure S26A NG2 (250 kDa)

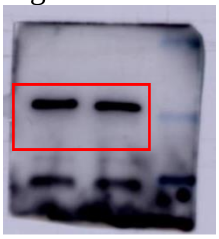

Figure S26A PDGFRβ (190 kDa)

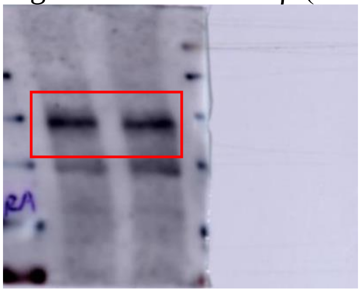

Figure S26A β-actin (42 kDa)

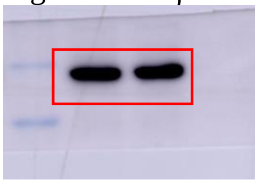

Figure S26B PDGFRα (190 kDa)

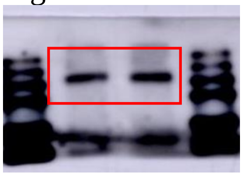

Figure S26B S100A4 (12 kDa)

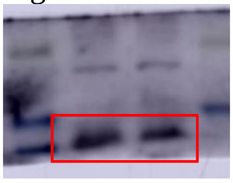

Figure S26B β-actin (42 kDa)

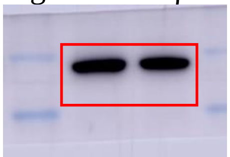

Figure S26C TCAF2 (100 kDa)

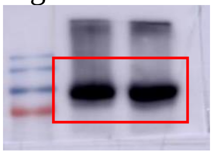

Figure S26B β-actin (42 kDa)

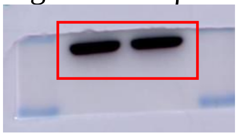

Figure S28

Figure S28B TCAF2 (100 kDa)

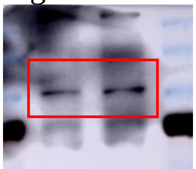

Figure S28B TCF21 (26 kDa)

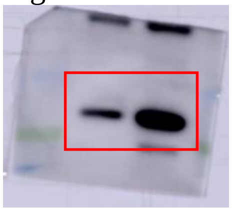

Figure S28B β-actin (42 kDa)

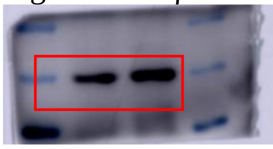

Figure S28C TCAF2 (100 kDa)

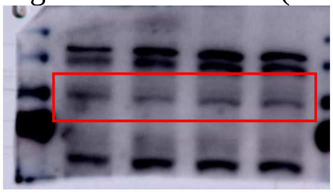

Figure S28C TCF21 (26 kDa)

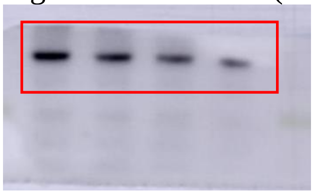

Figure S28C β-actin (42 kDa)

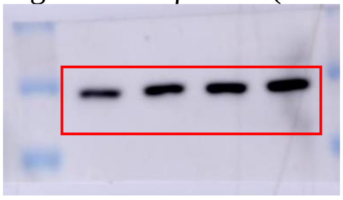

Supplement: Supplementary file 1 — Supporting Information [file ADVS-10-2302717-s001.pdf]
